# A new computational approach redefines the subtelomeric *vir* superfamily of *Plasmodium vivax*

**DOI:** 10.1186/1471-2164-14-8

**Published:** 2013-01-16

**Authors:** Francisco Javier Lopez, Maria Bernabeu, Carmen Fernandez-Becerra, Hernando A del Portillo

**Affiliations:** 1 Barcelona Centre for International Health Research, (CRESIB, Hospital Clínic-Universitat de Barcelona), Roselló 153, 1a planta (CEK building), 08036, Barcelona, Spain; 2 Institució Catalana de Recerca i Estudis Avançats (ICREA), Barcelona, Spain; 3 Present address: Andalusian Human Genome Sequencing Centre (CASEGH) Medical Genome Project (MGP) INSUR Building, Albert Einstein Street Cartuja 93 Scientific and Technology Park, 41092, Sevilla, Spain; 4 ICREA Barcelona Centre for International Health Research, (CRESIB, Hospital Clínic-Universitat de Barcelona), Barcelona, Spain

**Keywords:** Malaria, *Plasmodium vivax*, *vir* genes, VIR proteins, Subtelomeric multigene families, Sequence clustering, Similarity networks, Homology blocks

## Abstract

**Background:**

Subtelomeric multigene families of malaria parasites encode virulent determinants. The published genome sequence of *Plasmodium vivax* revealed the largest subtelomeric multigene family of human malaria parasites, the *vir* super-family, presently composed of 346 *vir* genes subdivided into 12 different subfamilies based on sequence homologies detected by BLAST.

**Results:**

A novel computational approach was used to redefine *vir* genes. First, a protein-weighted graph was built based on BLAST alignments. This graph was processed to ensure that edge weights are not exclusively based on the BLAST score between the two corresponding proteins, but strongly dependant on their graph neighbours and their associations. Then the Markov Clustering Algorithm was applied to the protein graph. Next, the Homology Block concept was used to further validate this clustering approach. Finally, proteome-wide analysis was carried out to predict new VIR members. Results showed that (i) three previous subfamilies cannot longer be classified as *vir* genes; (ii) most previously unclustered *vir* genes were clustered into *vir* subfamilies; (iii) 39 hypothetical proteins were predicted as VIR proteins; (iv) many of these findings are supported by a number of structural and functional evidences, sub-cellular localization studies, gene expression analysis and chromosome localization (v) this approach can be used to study other multigene families in malaria.

**Conclusions:**

This methodology, resource and new classification of *vir* genes will contribute to a new structural framing of this multigene family and other multigene families of malaria parasites, facilitating the design of experiments to understand their role in pathology, which in turn may help furthering vaccine development.

## Background

*Plasmodium vivax* is the most widely distributed human malaria parasite, with an at-risk population of 2.5 billion people [1]. The widely held misperception of *P. vivax* as being relatively infrequent, benign, and easily treated explains its nearly complete neglect across the range of biological and clinical research. However, recent reports provide abundant evidence challenging this paradigm (reviewed in [2,3]).

Antigenic variation is a regular feature of all *Plasmodium species,* enabling parasites to evade the immune system [4]. Genes putatively responsible for antigenic variation in *P. vivax*, termed *vir* (*P. vivax* variant genes), were initially identified by analyzing a chromosome end from a *P. vivax* wild isolate [5]. Later, the publication of the *P. vivax* Salvador I strain genome sequence allowed the redefinition of the *vir* gene repertoire revealing a total of 346 *vir* genes, including 80 fragments and/or pseudogenes, 12 different subfamilies (A-L) and 84 “unclustered genes” which were not associated to any subfamily [6]. In addition, their gene structure revealed a complex organization including genes with different numbers of exons (1–5) and different sizes (156 to 2316 bp). Of interest, this multigene family shares sequence homology with other *Plasmodium* species and is included within the variant gene superfamily (*Plasmodium* interspersed repeats, *pir*) together with *kir* in *P. knowlesi*, and the *cir*/*yir*/*bir* family in *P. chabaudi*, *P. yoelii* and *P. berghei*[7,8].

The function of the *vir* multigene family remains largely unknown. Analysis of the expressed *vir* repertoire in natural infections from individual parasites demonstrated that there is no allelic exclusion of *vir* genes and no clonal expression of VIR proteins at the surface of individually infected reticulocytes [9]. In addition, first-time infected *P. vivax* patients had naturally acquired antibodies capable of cross-reacting against different VIR proteins [9,10]. Moreover, only 160 deduced VIR proteins possess the PEXEL-like motif needed for exporting malarial proteins to the host cell surface [11]. Furthermore, subfamilies A and D share structural similarities, respectively, with the *P. falciparum surfin* and *Pfmc-2TM* multi-gene families [12]. This fact, together with the lack of PEXEL motifs in many of the VIR proteins, indicate that these proteins might have subcellular localizations other than the surface membrane of infected reticulocytes and different functions. This possibility, has been recently reported [13]. We thus reasoned that the original clustering of these 346 into a single superfamily might not be accurate and that some of these genes might belong to different multigene families.

In this work, a novel clustering procedure was applied to *vir* genes to re-analyze this subtelomeric multigene superfamily. Results presented here show that genes belonging to subfamilies A, D and H cannot longer be considered *vir* genes and that this computational approach facilitates grouping of unclustered genes, annotations of hypothetical proteins and studies of other multigene families. Procedures were implemented and integrated in web applications (http://bioinfold.fcrb.es/hb and http://bioinfold.fcrb.es/sequence_cluster).

## Results

### Contextual definitions

To facilitate the understanding of the developed method, we first introduce the definitions of several concepts: *E-*value threshold: the *E-value* parameter stands for the *Expect-value* threshold of the BLAST algorithm, which is used to calculate a similarity value for each two proteins [14].

The *Inflation* value is an input parameter of the Markov Clustering Algorithm [15]. It takes values in [1.1, 10.0] and determines the cluster granularity (the higher the *Inflation* value, the higher the granularity).

*Homology Block* concept is a key term regarding the search of conserved motifs. An homology block can be defined as a sequence profile determined from a multiple sequence alignment and modelled by a Hidden Markov Model (HMM).

Finally, note that we will name *unclustered* VIR proteins to those VIR proteins which were not previously associated to any subfamily, following the nomenclature by Carlton et al. [6], and *unclassified* VIR proteins to those predicted in this work that could not be associated to any multigene family.

### Novel computational methods show VIR subfamilies cluster as independent graph components

**Figure 1:**
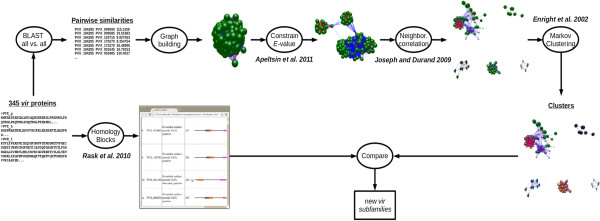
**Pipeline procedure to reclassify *vir *genes.** The procedure starts with the 345 VIR proteins annotated at PlasmoDB 7.2. Two graph pre-processing strategies [14,16] and the Markov Clustering Algorithm [15] are run to obtain sequence clusters. Then, the Homology Block concept [17] is used to further validate the clustering approach based on comparisons of conserved motifs. Graph figures were obtained using BioLayout [18].

**Figure 2:**
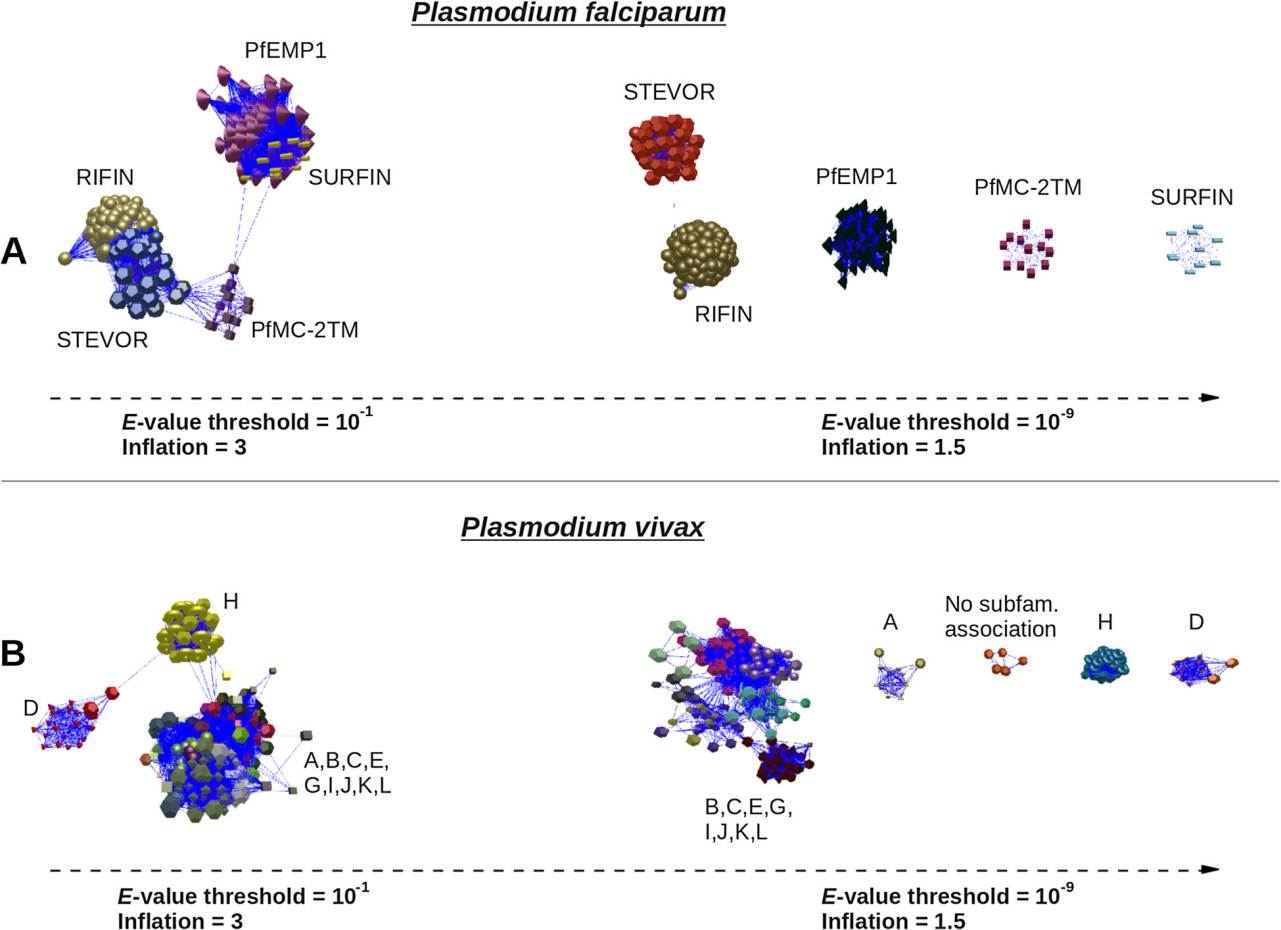
**Comparative clusterization of subtelomeric multigene families from *P. falciparum *and *P. vivax.*** Colors indicate cluster members. Different shapes indicate different (sub)families. **A**. Clustering result for the *P. falciparum* PfEMP1, PfMC-2TM, RIFIN, STEVOR and SURFIN subtelomeric families [19] at *E*-value = 10^-1^ and *Inflation* = 3 (left) and *E*-value = 10^-9^ and *Inflation* = 1.5 (right). **B**. Clustering result for the *P. vivax* VIR super-family [6] at *E*-value = 10^-1^ and *Inflation* = 3 (left) and *E*-value = 10^-9^ and *Inflation* = 1.5 (right). Graph figures were obtained using BioLayout [18].

The original annotation of *vir* genes was based on JIGSAW predictions, protein domains and sequence alignments [6]. Here, a new algorithm consisting of a pipeline having two recently reported pre-processing techniques [14,16] and the application of the Markov Clustering Algorithm [15] was implemented (Figure 1). In order to validate this clustering approach, we tested this algorithm using protein sequences from the RIFIN/STEVOR, Pfmc-2TM, fEMP1 and SURFIN subtelomeric multigene families of *P. falciparum*[19]. These multigene families have been demonstrated to have different sub-cellular localizations and functions [20-22]. We tested the clustering methodology by using distinct experimental set ups. As a starting point, we first determined the combination of *E*-value and *Inflation* (see “Methods”) that allowed this methodology to correctly classify these different subtelomeric multigene families of *P. falciparum*. Results showed that with the *E-*value threshold set at 10^-1^ and *Inflation* value equal to 3, excepting for a single *rifin* gene (PFC0045w) which clustered with *stevor* genes, the procedure correctly clustered the different families (Figure 2A). Using these same values, all previously annotated VIR subfamilies excepting subfamilies D and H constitute a group of strongly related proteins (Figure 2B).

The *E-*value was next progressively restricted until each *P. falciparum* subtelomeric multigene family formed an independent graph component, *E-*value threshold set at 10^-9^ and *Inflation* value equal to 1.5 (Figure 2A). Of note, PFC0045w is still included within the *stevor* group and a single link remains between PFI0070w (*rifin*) and MAL13P1.7 (*stevor*). Using these conditions, five independent graph components were observed for VIR proteins (Figure 2B). One of them represents the VIR core, containing most of the VIR proteins, another one is exclusively formed by proteins which are not associated to any subfamily (“unclassified proteins”), and the three remaining components represent subfamilies A, D, and H (Figure 2B). These results suggest that genes from subfamilies A, D, H and the group of “unclassified proteins” do not belong to *vir* genes but rather represent novel *P. vivax* multigene families.

### The VIR super-family and the new multigene families

**Figure 3:**
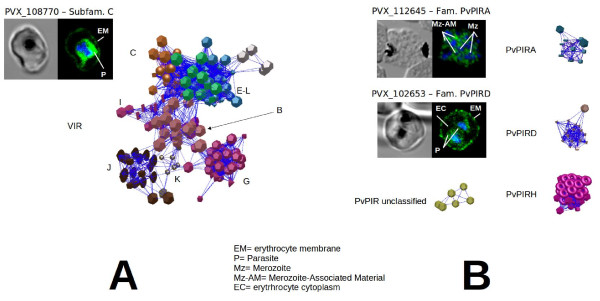
**New clustering of the VIR superfamily and subcellular localization of VIR-C, PvPIRA, and PvPIRD.** Clustering result for the *P. vivax* VIR super-family [6] at *E*-value = 10^-11^ and *Inflation* = 1.3. Colors indicate cluster members. Different shapes indicate different (sub)families. Graph figures were obtained using BioLayout [18]. Phase contrast and confocal immunofluorescence images of transgenic lines of *P. falciparum* 3D7 expressing VIR-C (PVX_108770), family PvPIRD (PVX_102635) and family PvPIRA (PVX_112645) proteins. Parasites were labelled with anti-HA (green) and DAPI for nuclear staining (blue).

To obtain a classification, as accurate as possible, of VIR proteins using this methodology, the combination of *E-*value and inflation was optimized for the VIR set (*E*-value 10^-11^ and *Inflation* = 1.3, see “Methods”). Using these values, a large cluster corresponding to the previously defined subfamilies B, C, E, G, I, J, K, remained inter-connected (Figure 3, Additional file 1). Moreover, proteins of subfamily L appeared strongly related with proteins of subfamily E and were thus clustered all together as subfamily E. Also, all but 16 of the previous 84 unclustered *vir* genes appear now integrated within these subfamilies. It is unlikely that the remaining 16 singletons form a new subfamily since they are too divergent to group together and 12 are annotated as pseudogenes or truncated proteins. Yet, 19 new unclustered VIR proteins were observed (Additional file 1). In total, 295 VIR proteins belonging to ten subfamilies and 19 unclustered *vir* genes are included into this new classification (Additional file 1). As expected, independent graph components corresponding to subfamilies A, D and H were also observed under these *E*-value and inflation parameters (Figure 3, Additional file 2). To further support predictions from the graph components, we used heterologous transfections of *P. falciparum* to express three individual genes encoding VIR-C (PVX_108770), family D (PVX_102635) and family A (PVX_112645) proteins. As shown in Figure 3 and as recently published [13], members of these multigene families have different sub-cellular localizations suggesting different functions.

### Proteome-wide analysis predicts 39 hypothetical proteins as belonging to variant proteins

To determine if this method could predict new VIR proteins, the algorithm was applied to the entire *P. vivax* proteome. 39 additional proteins appeared within the graph components of VIR core proteins, three with subfamily H and six with the independent graph component of “unclassified” proteins. Strikingly, all but one (PVX_123205, “CAF1 ribonuclease domain containing protein” contained in the non-clustered graph component), are annotated as “hypothetical proteins” (Additional file 1). In order to get additional information of the 47 hypothetical proteins (48 - PVX_123205), the genomic location of the genes was investigated (Additional file 3): (i) 21 are located in subtelomeric regions. Out of the 21, 14 co-localize with members of their subfamilies in assembled chromosomes. Seven are subtelomeric but did not cluster with members of their own subfamilies due to the fact that most members of those subfamilies were not assembled. (ii) 3 genes are internally located, the three of them contained in the independent graph component of “unclassified” proteins (PVX_119620, PVX_092630 and PVX_123205). Of note, no hits were found between genes and PFL0030c (VAR2CSA) using PlasmoDB 7.2 sequences, NCBI BLAST 2.2.27 and E-value threshold = 0.01. (iii). The remaining 23 genes could not be assigned to any particular chromosomes since most chromosome ends from the genome of the *P. vivax* SalI strain remain unassembled [6].

### Comparisons of conserved motifs among the newly defined VIR and non-VIR proteins reinforce this new classification

The Homology Block (HB) concept [17] was used to further validate this clustering algorithm based on comparisons of conserved motifs (see “Methods”). The prediction being that HBs from subfamilies A, D and H should have little or no intersections with the remaining subfamilies clustering as VIR proteins. First, we demonstrated that HBs capture previously defined VIR conserved motifs [6] (Additional file 4). Next, we aimed to determine whether there were cluster-specific HBs. Hence, for each cluster, we counted the number of HBs which were unique to that particular cluster and the number of those shared with one each other (Additional file 5). Noticeably, the proportion of specific/shared is either well-balanced or a considerably greater number of shared HBs appear in the VIR subfamilies. In contrast, an outstanding proportion of specific HBs were observed for families D and H (100% and 91% respectively). Note that subfamily A also formed an independent graph component when setting the BLAST *E-*value threshold at 10^-9^ (Figure 2). However, unlike subfamilies D and H, subfamily A shares 41% of HBs with the rest of VIR proteins. As expected, the analysis of the HB composition of proteins belonging to the same cluster revealed that the HB architecture is quite well conserved between members of the same subfamily. Moreover, this HB structure is also conserved in many of the hypothetical proteins predicted to be VIR members. Finally, InterproScan predictions were also obtained in order to provide a more general view of putative domains. It is worth noting that no matches were obtained by InterproScan for members of the newly defined subfamily H. (Additional file 6).

All together, these results validate the new classification of *vir* genes and exclude members from subfamilies A, D and H as VIR proteins. To avoid confoundings with their previous nomenclature as well as with the gene families (*Pv-fam-a-e* and *Pv-fam-g-i*) identified in the genome issue [6], we propose to term them PvPIRA, PvPIRD, and PvPIRH as they clearly fall into the PIR proteins super-family [8].

### High expression levels of a subset of *vir* genes and most *pvpirH* genes in patients with symptomatic *P. vivax* infections

**Figure 4:**
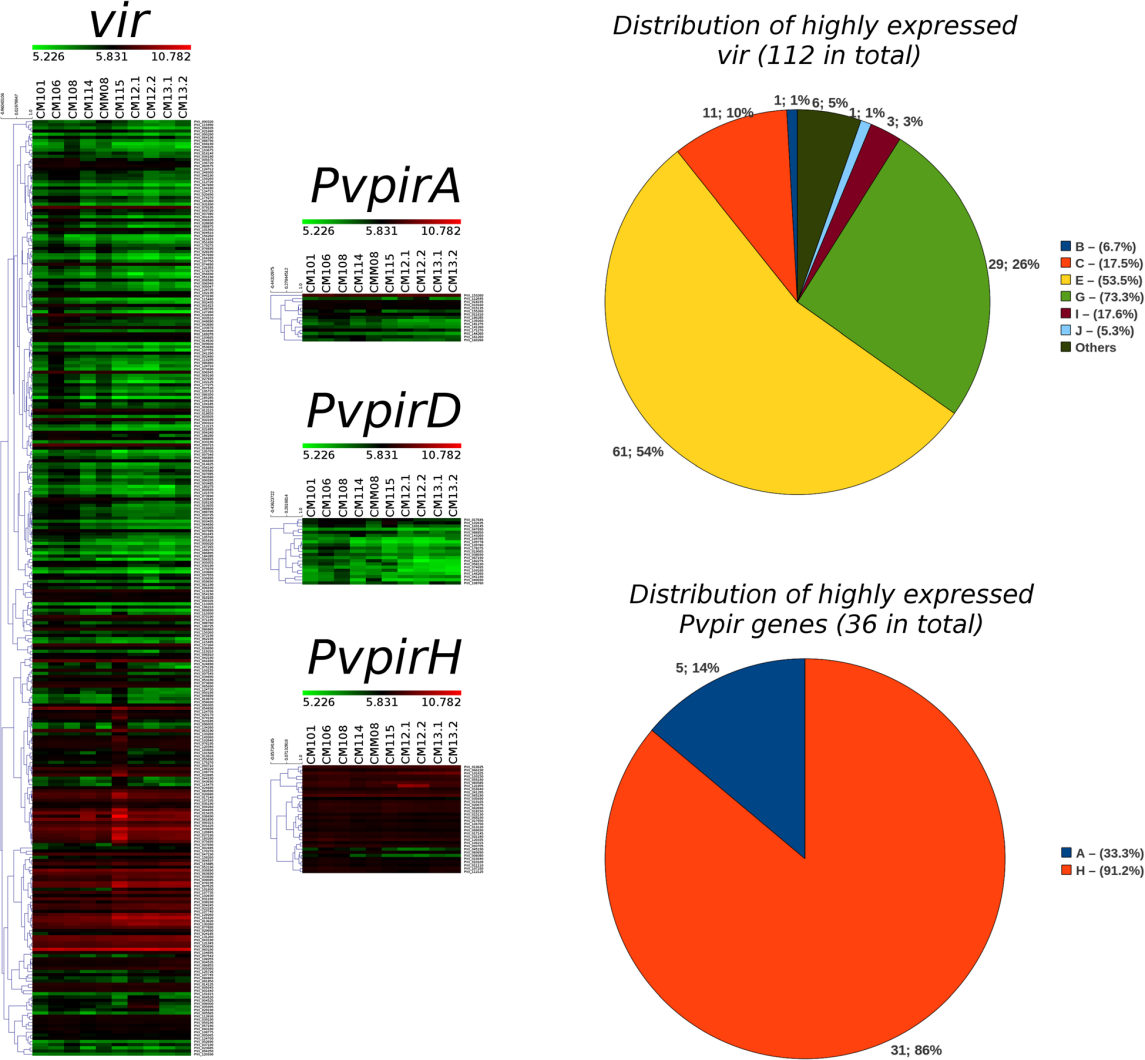
**Gene expression profiles of *vir *and *pvpirA, pvpirD, *and *pvpirH *genes in symptomatic *P. vivax *patients.** Left panel. Heatmaps drawn for the expression data of ten *P. vivax* patients [23]. Only *vir* and the newly defined *pvvirA, pvpirD and pvpirH* are shown. Color scale spans from 5.226 to 10.782 (upper bar), which represent the minimum and maximum expression values observed for this set of genes and samples (median value = 5.831). Dendrograms on the left of heatmaps show the result of average-linkage hierarchical algorithm. Right panel. Pie charts showing the distribution of expressed genes with expression values ≥ 5.831 for all samples. Percentages next to each (sub)family label indicate the proportion of genes within the corresponding (sub)family.

To illustrate the value of this new classification, we determined the expression pattern of *vir* genes and the newly defined *pvpirA, pvpirD* and *pvpirH* genes in parasites obtained from *P. vivax* patients [23]. This dataset contains the expression level of 5435 *P. vivax* genes in 10 blood samples of patients with typical symptoms of malaria. Interestingly, unlike members of the newly defined *pvpirA* and *pvpirD* genes, a subset of *vir* genes (38%) and most genes (91%) of the *pvpirH* family present high expression values in samples from all patients (Figure 4). Analysis of the set of *vir* genes expressed in all the samples (rows in red and dark red in Figure 4), showed no correlation with any one particular subfamily. These results thus illustrate the value of this new classification as they identified a subset of *vir* genes and *pvpirH* genes likely associated with clinical symptoms.

## Discussion

The current definition and classification of *vir* genes was based on JIGSAW predictions, protein domains and sequence alignments [6]. Our approach also used homology-based BLAST analysis to build a protein graph; yet, the graph pre-processing strategies ensure that the weight of an edge is not exclusively based on the sequence similarity between the two proteins, but strongly dependant on the relations of their neighbours. In other words, the appearance of a protein within a group of nodes is a reliable indicator that there exists more than a simple sequence similarity relation between them. We found the application of the two pre-processing strategies to be essential for a good performance of the MCL algorithm over the VIR set. Using this methodology, our results corroborated the original classification of *vir* genes into different subfamilies. Moreover, results correlate well with OrthoMCL groups which also supports the good functioning of the procedure, but presents some significant differences mainly for subfamilies C, K, G and some of the groups of previously unclustered genes (Additional file 7). In addition, the whole clustering pipeline and results visualization allowed us to assess the strength and evolution of sequence similarities. Thus three of the subfamilies (A, D and H) formed independent graph components at an *E*-value and *Inflation* value where different multigene families of *P. falciparum* were observed as independent graph components. Hence, subfamilies A, D and H could be considered members of different families belonging to the PIR super-family and different data from subcellular and chromosomal locations and homology blocks fully supported this consideration. Accordingly, we propose to term these families PvPIRA, PvPIRD and PvPIRH to avoid confoundings with VIR proteins and with members of the *Pv-fam-a-e* and *Pv-fam-g-i* families described in the genome issue [6].

### The Homology blocks concept reinforces the new classification of *vir* genes

To support this clustering approach, we applied the homology block (HB) concept originally coined by Smith and co-workers [24], and used to analyze the *P. falciparum* Erythrocyte Member Protein 1 (PfEMP1) as an iterative procedure for mining HBs from a set of PfEMP1 protein sequences [17]. The use of HBs facilitated a better classification and structural framing of *var* genes allowing recently the discovery of unique domain cassette-encoding *var* genes associated with severe disease in children [25]. Our results revealed that all of the HBs found in proteins of the PvPIRD proteins were family-specific. Likewise, all but two of the conserved motifs found in PvPIRH proteins were also family-specific. In contrast, motif-specific enrichment was not observed in proteins from the PvPIRA family which presented a balanced proportion of specific/shared HBs with VIR proteins. PvPIRA family still remains connected with VIR proteins at less stringent *E*-value and Inflation parameters partly explaining this result. Yet, it forms a completely independent graph component at E-value and inflation parameters where all known *P. falciparum* multigene families are independent. In addition, a member of this family has a different sub-cellular location as that of another member from the VIR family. We thereby consider PvPIRA an independent new subtelomeric family of *P. vivax*.

### The clustering procedure facilitates the annotation of hypothetical proteins and evolutionary relatedness of malaria multigene families

Current annotation of the *P. vivax* proteome represents a great challenge as close to 60% of it remains annotated as hypothetical proteins. Remarkably, our approach predicted 39 hypothetical proteins as VIR proteins (Additional file 1). Moreover, data on location for those that could be assigned to assembled chromosomes revealed that they are located within subtelomeric regions. In addition, there were many occurrences of HBs related to VIR proteins reinforcing the predictions that these hypothetical proteins indeed represent VIR proteins (Additional file 6). Three of these proteins were associated with the “unclassified” group and are located in internal regions. VAR2CSA is an internal *var* gene directly involved in pregnancy-associated pathology in *P. falciparum*[26]. Whether any of these putative internal variant genes are related to pregnancy-associated pathology in *P. vivax* is presently unknown.

**Figure 5:**
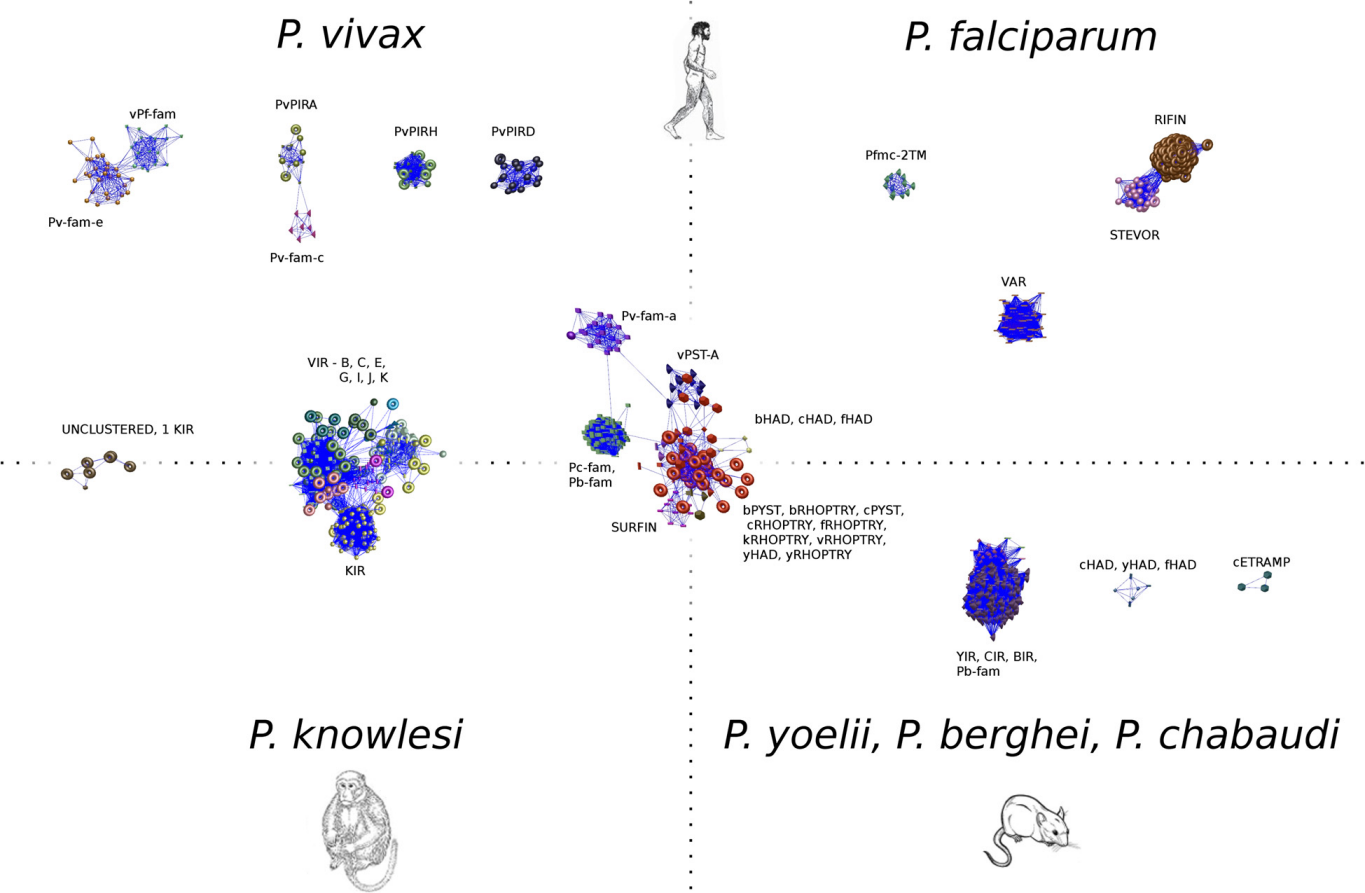
**Protein similarity relations among several *Plasmodium *subtelomeric families.** Results obtained when running the clustering procedure over the subtelomeric families of *P. vivax* (Pv or v): VIR, PvPIRA-D-H, Pv-fam, v-Pf-fam, vRHOPTRY, v-PST-A; *P. falciparum* (Pf or f): VAR, RIFIN, STEVOR, Pfmc-2TM, SURFIN, fRHOPTRY, fHAD; *P. knowlesi* (k): KIR, kRHOPTRY; *P. chabaudi* (Pc or c): CIR, Pc-fam, cRHOPTRY, cHAD, cPYIST, cETRAMP; *P. berghei* (Pb or b): BIR, Pb-fam, bRHOPTRY, bHAD, bPYIST, and *P. yoelii* (Py or y): YIR, yRHOPTRY, yHAD using an E-value 10^-11^ and Inflation of 1.3. The position of the clusters with respect to the axes is just a qualitative representation. Axes do not represent any metric. Graph figures were obtained using BioLayout [27].

To determine if this clustering algorithm can facilitate annotation of proteins other than VIR and further our understanding of other malaria subtelomeric families, we ran the pipeline over the entire proteomes of *P. falciparum*, *P. knowlesi*, and the rodent malarias *P. yoelii, P. chabaudi* and *P. berghei* (Figure 5). Several inter-connected graph components belonging to different families of different species could be readily observed (Figure 5). For instance, the SURFIN family of *P. falciparum* shares relations with different families from all species suggesting a common origin. These results, however, need to be taken with caution as excepting for *P. falciparum*, remaining genomes are highly unassembled at chromosome ends where most of these families reside. Yet, as better coverage and assembling of these regions is achieved, this tool should facilitate the design of experiments to better understand the evolution and function of malaria subtelomeric families.

### VIR proteins and pathology

The function of VIR proteins and other multigene families of this species remain largely unknown. Yet, recent evidences have demonstrated that a VIR protein belonging to subfamily C was exported and exposed at the surface of infected erythrocytes and that it mediated specific binding to the ICAM-1 endothelial receptor under flow physiological conditions [13]. This result, together with other evidence of in vitro cytoadherence of *P. vivax*-infected reticulocytes [28,29], indicates that this species, similar to *P. falciparum*, can cytoadhere. We thus computationally searched for other putative adhesins within members of VIR, PIRA, PIRD, and PIRH proteins using the MAAP predictor [30] (see “Methods”). Interestingly, only members representing subfamilies C and E as well as H proteins presented positive predictions. Thus, 39% (12/31) of H proteins present positive predictions, while only 7% (3/42) and 6% (5/88) of C and E VIR proteins, respectively, yielded a positive score. It is worth noting here that MAAP was trained with *P. falciparum* data, which implies that its performance may not be optimal when applied over *P. vivax* proteins. Yet, these data reinforce the view that variant proteins of *P. vivax* are involved in cytoadherence and pathology. In the absence of further experimental evidence this remains to be fully demonstrated.

## Conclusions

A new computational approach was applied to revisit the original classification of *vir* genes, the largest subtelomeric multigene superfamily of human malaria parasites. Applying this pipeline, the *vir* gene super-family was redefined by excluding members of subfamilies A, D and H, by including unclusterd genes, and by facilitating the genome-wide annotation of 39 hypothetical proteins as new VIR proteins. In addition, analysis of gene expression data from febrile *P. vivax* patients illustrated the value of this new classification as it showed high expression levels of most genes belonging to the PvPIRH multigene family. Last, the clustering approach was extended to other subtelomeric multigene families of malaria parasites. It will thus improve the design of experiments to determine the role of subtelomeric multigene families in pathology, which in turn may help furthering vaccine development against malaria.

## Methods

### Data

Sequence information of the *P. vivax*, *P. falciparum*, *P. chabaudi*, *P. knowlesi* and *P. yoelii* predicted proteomes was obtained from PlasmoDB release 7.2 (May 2011) [31]. *P. berghei* sequences were obtained from PlasmoDB release 7.1 (Nov 2010). In addition, release A_25.0 of Pfam (March 2011) was downloaded from the HHpred ftp server [32] (ftp://toolkit.lmb.uni-muenchen.de/HHsearch/databases/).

### Protein sequence clustering

The clustering procedure is based on a graph representation of the set of proteins, where nodes are the actual proteins and the weighted edges indicate the similarity relations between them. In our particular case, the weights of the edges are calculated as a function of the BLAST score obtained for each two proteins. A pipeline consisting of three different steps is followed to get the final set of clusters: 1) Estimating an appropriate BLAST *E-*value threshold, 2) Similarity value calculation and 3) Running the Markov Clustering Algorithm (MCL). The complete procedure was implemented and integrated in a web application (http://bioinfold.fcrb.es/sequence_cluster).

#### Estimating an appropriate BLAST *E-*value threshold

The *E-value* threshold stands for the *Expect-value* threshold of the BLAST algorithm, and describes the number of hits one can “expect” to see just by chance when searching a database of a particular size. Sequence hits with an *E*-value greater than the threshold are discarded. Thus, an appropriate *E-*value threshold is essential to remove superfluous edges from the graph which introduce noise and disturb the clustering process. The heuristic reported by Apeltsin *et al.*[14] was followed. Briefly, it consists of an iterative procedure which starts running a BLAST all vs. all with a high *E-*value threshold (i.e. 1) [33]. An initial graph is thus built. Then, a parameter called *Nsv* by the authors is calculated as a function of the number of connected nodes and the number of edges in the graph:

(1) $$N_{\mathrm{sv}}=\frac{\;\mathrm{Edges}}{\;\mathrm{Connected}\_\_\mathrm{nodes}}$$

Next, the *E-*value threshold is divided by 10 and the algorithm starts again by rebuilding the graph (unconnected nodes are removed). The procedure stops when the *Nsv* no longer decreases and begins to increase. The underlying reasoning is that those edges removed at the initial iterations connect proteins from different families, and therefore do not cause node removals. However, if the *Nsv* trend varies, is due to the removal of edges which link proteins within the same family. This, in turn, causes the isolation of nodes which are therefore deleted. Further details can be found in [14].

#### Calculating similarity values

Once the *E*-value threshold is set, the final weigths of the edges are calculated as a function of the similarity between each two of proteins. For each pair of sequences *x* and *y*, initial similarity values are calculated based on the High-scoring Segment Pairs (HSPs) returned by BLAST. The combination of non-overlapping HSPs which yields the highest score is obtained, and the corresponding bit score used as the initial similarity value, *S*_*init*_*(x,y)*. Then, the strategy proposed by Joseph *et al.*[16] is applied to weight these similarity values. Let *w*_*x*_ be the vector of similarity values between *x* and the rest of proteins. For each pair of nodes *x* and *y*, the Neighbourhood Correlation score, *NC*_*xy*_, is calculated as the Pearson Correlation Coefficient between *w*_*x*_ and *w*_*y*_. Thus, the final similarity value between *x* and *y* and therefore the final weight of the edge than links *x* and *y* in the graph, is calculated as:

(2) $$S\left(x,y\right)=S_{\mathrm{init}}\left(x,y\right)\;.NC_{\mathrm{xy}}$$

Hence, it is important to emphasize that the strength of the association between two given proteins is not exclusively dependant on the similarity score, but also strongly conditioned on its neighbours and their associations. This step resulted to be of great importance to reducing noise and avoiding problems such as the domain chaining. Further details can be found in [16].

#### Markov clustering algorithm (MCL)

The Markov Clustering Algorithm (MCL) is a well-known procedure which has been extensively used for protein clustering [15]. Moreover, large scale projects such as the construction of the OrthoMCL [34] database made use of this algorithm for their purposes. It is a robust and efficient algorithm for graph clustering which uses an internal matrix representation. The underlying idea of the procedure is to simulate random walks within the graph, assuming that the number of longer paths between two arbitrary nodes in a given cluster is high. On the other hand, random walks on the graph will infrequently go from one cluster to another. Thus, the algorithm iteratively modifies the probabilities of random walks through the graph, by alternating two matrix operators called *expansion* and *inflation* until convergence. An important advantage of this algorithm is that it only requires to provide one parameter, the *Inflation*, which takes values in [1.1, 10.0] and determines the cluster granularity (the higher the *Inflation* value, the higher the granularity). Please, refer to the work reference in [15] for further details.

#### Selecting clustering parameters

Two are the main parameters that had to be set for family classification: *E-*value threshold and *Inflation* value. First, the heuristic reported by Apeltsin *et al.* was followed as described above. The procedure estimated a minimum *E-*value threshold of 10^-44^. Setting the *Inflation* value to 1.3, good clustering results were obtained which correlated well with the previous classification. Moreover, subfamily C was clearly divided in two different groups and two distinct subtypes of proteins could be unveiled among E members. Nevertheless, two facts evidenced that this *E-*value threshold was extremely restrictive. First, the HB architecture of the some subfamilies such as J or D, clearly indicated that highly similar proteins were being distributed in distinct clusters. Second, due to the highly restrictive threshold, up to 167 proteins lost all of their similarity associations with the rest of VIR members, and therefore were not included in the final clustering step. This meant that a lot of information was being missed and that the procedure was overestimating the *E-*value threshold. Recall here that the strategy is based on a heuristic and therefore may not provide the optimal solution. Thus, we looked for an *E-*value threshold as close as possible to the 10^-44^ which did not cause such a loss of data. It was observed that with the *E-*value threshold set at 10^-11^ just 16 proteins were discarded. Moreover, all of them were labelled as “Not clustered“ by previous authors when the original VIR subfamilies were defined.

Once the *E-*value threshold was set, an appropriate *Inflation* value was estimated. A range of values were tested. Clustering results were visualized using BioLayout [18] and compared with the original classification, as well as with the HB architecture of the sequences. After manual inspection of the results, an *Inflation* value of 1.3 was chosen, since clusters were able to appropriately capture the structure of the graph, correlated well with the previous subfamilies and were in agreement with the HB architecture of the sequences.

### Homology blocks

#### Mining homology blocks

An homology block represents a sequence pattern. Sequence patterns represented by Homology Blocks are usually observed after the alignment of multiple sequences which share a given motif. This motif (sequence pattern) is then modelled by a particular type of probabilistic models, called Hidden Markov Models (HMM). Thus, an homology block can be defined as a sequence profile determined from a multiple sequence alignment and modelled by a Hidden Markov Model (HMM). The procedure proposed by Rask *et al.*[17] was followed. The algorithm consists of an iterative process which repeats three main steps: 1) Selecting 100 promising *seeds* from the sequence database, 2) Building an HB for each seed and 3) Selecting the best HB and removing its occurrences. Since the original procedure is comprehensively described in the paper referenced in [17], we here describe just the three significant modifications of the original algorithm that were included in steps 1) and 2): *1) Selecting promising seeds.* A seed can be defined as a promising short sequence extracted from the database. A score value is calculated for each seed which is, roughly, based on the number of homologies that the corresponding seed presents in the database [17]. After sorting the set of seeds according to their punctuation, it was observed that in many cases the score of the seed ranked at position 100, was the same than that of the seeds at position 101, 102 and so on. Therefore, it makes no sense to select only 100 seeds. Thus, some flexibility was allowed in the selection procedure, and all those seeds which score equals that of seed 100 were included in the selected set.

*2) Saving HBs for posterior selection.* Each time an HB is built from a certain seed, the original algorithm saves it for posterior selection in step 3. However, it was observed, that many low conserved motifs representing unsignificant sequence profiles were generated. In other words, many HBs with an empty logo appeared [27]. Hence, for each HB, the conservation level at each position was calculated [27]. Small sample correction was also incorporated. Those HBs which did not contain at least one significantly conserved position (conservation level > 0) were discarded.

*3) Exhausting the seed list.* If certain iteration returns no homology blocks, the next 200 seeds in the list are processed. The procedure is repeated until the seed list is empty. This ensures that all the seeds are processed and that no HB is lost.

#### Comparing HBs

The comparison between homology blocks was done by comparing their corresponding HMMs. Söding proposed a dynamic programming algorithm for aligning two given HMMs and developed the package HHpred [32]. The software HHsearch (included in HHpred 1.5.0) was used for comparing the set of HBs with the Pfam database. For each pair of HMMs, HHsearch returns the probability that both models overlap (range [0, 100]). Likewise, HHsearch was used for comparing the HBs with the previously defined MEME motifs. In this case, HMMs for the MEME motifs were built by using HMMER 3.0 [35].

Finally, a clustering algorithm was applied over the set of HBs to identify redundancies. The average-linkage hierarchical algorithm was used, setting distance threshold at 70. The distance measure was calculated as 100-*HHsearch_probability* (Additional file 4).

### Transgenic lines generation and indirect immunofluorescence assays

*P. falciparum* culture, parasite transfection and indirect immunofluorescence assays were done as described in [13].

### Adhesins prediction

Computational prediction of putative adhesins was carried out by applying MAAP [30]. The score threshold was set to 0.7 as suggested by the MAAP authors for the *P. vivax* proteome.

## Competing interests

All authors declare that they have no competing interests.

## Authors’ contributions

FJL suggested, implemented and performed computational analyses. MB and CFB suggested and performed biological experiments. HAP idealized and coordinated the study. FJL and HAP drafted the manuscript. All authors read and approved the final manuscript.

## Supplementary Material

Additional file 1 **New VIR subfamilies.** Excel sheet containing the new classification proposal of the VIR proteins. The list of subfamilies is shown along with their members. Rows in red indicate that the HB architecture of the corresponding protein does not clearly match with the rest of members in the subfamily. Click here for file

Additional file 2 **New subtelomeric PvPIRA, PvPIRD and PvPIRH families.** Excel sheet containing the new subtelomeric families derived from previous VIR members. Rows in red indicate that the HB architecture of the corresponding protein does not clearly match with the rest of members in the subfa. Click here for file

Additional file 3 **Genome-wide distribution of newly predicted *vir*genes.** Figure showing the chromosomal location of newly annotated hypothetical proteins as VIR proteins. For each chromosome, two rows of colored boxes are shown: one row illustrates the genomic location of the *vir* members (red) while the other illustrates the location of the putative *vir* (green) in that chromosome. Only genes annotated at specific chromosomes are shown. Click here for file

Additional file 4 **HB search results.** Excel table with the list of Homology Blocks, their similarities with previously defined conserved motifs and PFAM annotations. Click here for file

Additional file 5 **Motif distribution across clusters.** Two tables in an Excel data sheet showing the distribution of the conserved HBs across clusters: *i)* for each cluster, the number (and proportion) of HBs shared with other clusters is shown, as well as the number (and proportion) of cluster-specific HBs; *ii)* rows represent HBs, columns represent sequence clusters (subfamilies and families). The first row in the table (after the header) contains the size of each cluster. First column shows HB identifiers. Each cell in the table contains the number of sequences in a given cluster that contain the corresponding HB (and the proportion in brackets). Click here for file

Additional file 6 **Conserved motifs composition.** Composition of clusters and the conserved motif structure of each of the proteins in the original VIR set, as well as the hypothetical proteins grouped with them. An illustration of the most representative homology blocks in each family is also included as well as InterproScan predictions for newly defined (sub)families. Click here for file

Additional file 7 **Comparison with OrthoMCL5. Results of the comparison between *vir *, *Pvpir *(sub)families and OrthoMCL5 groups.** Click here for file

## References

[B1] Guerra CA Howes RE Patil AP Gething PW Van Boeckel TP Temperley WH Kabaria CW Tatem AJ Manh BH Elyazar IR The international limits and population at risk of *Plasmodium vivax* transmission in 2009 PLoS Negl Trop Dis 2010 4 e774 20689816 10.1371/journal.pntd.0000774 PMC2914753

[B2] Baird JK Neglect of *Plasmodium vivax* malaria Trends Parasitol 2007 23 533 539 17933585 10.1016/j.pt.2007.08.011

[B3] Mueller I Galinski MR Baird JK Carlton JM Kochar DK Alonso PL del Portillo HA Key gaps in the knowledge of *Plasmodium vivax*, a neglected human malaria parasite Lancet Infect Dis 2009 9 555 566 19695492 10.1016/S1473-3099(09)70177-X

[B4] Scherf A Lopez-Rubio JJ Riviere L Antigenic Variation in *Plasmodium falciparum* Annu Rev Microbiol 2008 62 445 470 18785843 10.1146/annurev.micro.61.080706.093134

[B5] del Portillo HA Fernandez-Becerra C Bowman S Oliver K Preuss M Sanchez CP Schneider NK Villalobos JM Rajandream MA Harris D A superfamily of variant genes encoded in the subtelomeric region of *Plasmodium vivax* Nature 2001 410 839 842 11298455 10.1038/35071118

[B6] Carlton JM Adams JH Silva JC Bidwell SL Lorenzi H Caler E Crabtree J Angiuoli SV Merino EF Amedeo P Comparative genomics of the neglected human malaria parasite *Plasmodium vivax* Nature 2008 455 757 763 18843361 10.1038/nature07327 PMC2651158

[B7] Janssen CS Barrett MP Turner CM Phillips RS A large gene family for putative variant antigens shared by human and rodent malaria parasites Proc Biol Sci 2002 269 431 436 11886633 10.1098/rspb.2001.1903 PMC1690903

[B8] Cunningham D Lawton J Jarra W Preiser P Langhorne J The pir multigene family of *Plasmodium*: antigenic variation and beyond Mol Biochem Parasitol 2010 170 65 73 20045030 10.1016/j.molbiopara.2009.12.010

[B9] Fernandez-Becerra C Pein O de Oliveira TR Yamamoto MM Cassola AC Rocha C Soares IS de Braganca Pereira CA del Portillo HA Variant proteins of *Plasmodium vivax* are not clonally expressed in natural infections Mol Microbiol 2005 58 648 658 16238616 10.1111/j.1365-2958.2005.04850.x

[B10] Oliveira TR Fernandez-Becerra C Jimenez MC Del Portillo HA Soares IS Evaluation of the acquired immune responses to *Plasmodium vivax* VIR variant antigens in individuals living in malaria-endemic areas of Brazil Malar J 2006 5 83 17026752 10.1186/1475-2875-5-83 PMC1626480

[B11] Marti M Good RT Rug M Knuepfer E Cowman AF Targeting malaria virulence and remodeling proteins to the host erythrocyte Science 2004 306 1930 1933 15591202 10.1126/science.1102452

[B12] Merino EF Fernandez-Becerra C Durham AM Ferreira JE Tumilasci VF D'Arc-Neves J Da Silva-Nunes M Ferreira MU Wickramarachchi T Udagama-Randeniya P Multi-character population study of the vir subtelomeric multigene superfamily of *Plasmodium vivax*, a major human malaria parasite Mol Biochem Parasitol 2006 149 10 16 16730808 10.1016/j.molbiopara.2006.04.002

[B13] Bernabeu M Lopez FJ Ferrer M Martin-Jaular L Razaname A Corradin G Maier AG Del Portillo HA Fernandez-Becerra C Functional analysis of *Plasmodium vivax* VIR proteins reveals different subcellular localizations and cytoadherence to the ICAM-1 endothelial receptor Cell Microbiol 2011 14 386 400 22103402 10.1111/j.1462-5822.2011.01726.x

[B14] Apeltsin L Morris JH Babbitt PC Ferrin TE Improving the quality of protein similarity network clustering algorithms using the network edge weight distribution Bioinformatics 2011 27 326 333 21118823 10.1093/bioinformatics/btq655 PMC3031030

[B15] Enright AJ Van Dongen S Ouzounis CA An efficient algorithm for large-scale detection of protein families Nucleic Acids Res 2002 30 1575 1584 11917018 10.1093/nar/30.7.1575 PMC101833

[B16] Joseph JM Durand D Family classification without domain chaining Bioinformatics 2009 25 i45 i53 19478015 10.1093/bioinformatics/btp207 PMC2687961

[B17] Rask TS Hansen DA Theander TG Gorm Pedersen A Lavstsen T *Plasmodium falciparum* erythrocyte membrane protein 1 diversity in seven genomes--divide and conquer PLoS Comput Biol 2010 6 10.1371/journal.pcbi.1000933 PMC2940729 20862303

[B18] Theocharidis A van Dongen S Enright AJ Freeman TC Network visualization and analysis of gene expression data using BioLayout Express(3D) Nat Protoc 2009 4 1535 1550 19798086 10.1038/nprot.2009.177

[B19] Gardner MJ Hall N Fung E White O Berriman M Hyman RW Carlton JM Pain A Nelson KE Bowman S Genome sequence of the human malaria parasite *Plasmodium falciparum* Nature 2002 419 498 511 12368864 10.1038/nature01097 PMC3836256

[B20] Joannin N Abhiman S Sonnhammer EL Wahlgren M Sub-grouping and sub-functionalization of the RIFIN multi-copy protein family BMC Genomics 2008 9 19 18197962 10.1186/1471-2164-9-19 PMC2257938

[B21] Winter G Kawai S Haeggstrom M Kaneko O von Euler A Kawazu S Palm D Fernandez V Wahlgren M SURFIN is a polymorphic antigen expressed on *Plasmodium falciparum* merozoites and infected erythrocytes J Exp Med 2005 201 1853 1863 15939796 10.1084/jem.20041392 PMC2213267

[B22] Niang M Yan Yam X Preiser PR The *Plasmodium falciparum* STEVOR multigene family mediates antigenic variation of the infected erythrocyte PLoS Pathog 2009 5 e1000307 19229319 10.1371/journal.ppat.1000307 PMC2637975

[B23] Westenberger SJ McClean CM Chattopadhyay R Dharia NV Carlton JM Barnwell JW Collins WE Hoffman SL Zhou Y Vinetz JM Winzeler EA A systems-based analysis of *Plasmodium vivax* lifecycle transcription from human to mosquito PLoS Negl Trop Dis 2010 4 e653 20386602 10.1371/journal.pntd.0000653 PMC2850316

[B24] Smith JD Subramanian G Gamain B Baruch DI Miller LH Classification of adhesive domains in the *Plasmodium falciparum* erythrocyte membrane protein 1 family Mol Biochem Parasitol 2000 110 293 310 11071284 10.1016/s0166-6851(00)00279-6

[B25] Lavstsen T Turner L Saguti F Magistrado P Rask TS Jespersen JS Wang CW Berger SS Baraka V Marquard AM *Plasmodium falciparum* erythrocyte membrane protein 1 domain cassettes 8 and 13 are associated with severe malaria in children Proc Natl Acad Sci U S A 2012 109 E1791 1800 22619319 10.1073/pnas.1120455109 PMC3387094

[B26] Rogerson SJ Hviid L Duffy PE Leke RF Taylor DW Malaria in pregnancy: pathogenesis and immunity Lancet Infect Dis 2007 7 105 117 17251081 10.1016/S1473-3099(07)70022-1

[B27] Crooks GE Hon G Chandonia JM Brenner SE WebLogo: A Sequence Logo Generator Genome Res 2004 14 1180 1190 10.1101/gr.849004 PMC419797 15173120

[B28] Carvalho BO Lopes SC Nogueira PA Orlandi PP Bargieri DY Blanco YC Mamoni R Leite JA Rodrigues MM Soares IS On the cytoadhesion of *Plasmodium vivax*-infected erythrocytes J Infect Dis 2010 202 638 647 20617923 10.1086/654815

[B29] Chotivanich K Udomsangpetch R Suwanarusk R Pukrittayakamee S Wilairatana P Beeson JG Day NP White NJ *Plasmodium vivax* adherence to placental glycosaminoglycans PLoS One 2012 7 e34509 22529919 10.1371/journal.pone.0034509 PMC3328474

[B30] Ansari FA Kumar N Bala Subramanyam M Gnanamani M Ramachandran S MAAP: malarial adhesins and adhesin-like proteins predictor Proteins 2008 70 659 666 17879344 10.1002/prot.21568

[B31] Aurrecoechea C Brestelli J Brunk BP Dommer J Fischer S Gajria B Gao X Gingle A Grant G Harb OS PlasmoDB: a functional genomic database for malaria parasites Nucleic Acids Res 2009 37 D539 543 18957442 10.1093/nar/gkn814 PMC2686598

[B32] Soding J Biegert A Lupas AN The HHpred interactive server for protein homology detection and structure prediction Nucleic Acids Res 2005 33 W244 248 15980461 10.1093/nar/gki408 PMC1160169

[B33] Altschul SF Gish W Miller W Myers EW Lipman DJ Basic local alignment search tool J Mol Biol 1990 215 403 410 2231712 10.1016/S0022-2836(05)80360-2

[B34] Li L Stoeckert CJ Jr Roos DS OrthoMCL: identification of ortholog groups for eukaryotic genomes Genome Res 2003 13 2178 2189 12952885 10.1101/gr.1224503 PMC403725

[B35] Eddy SR A Probabilistic Model of Local Sequence Alignment That Simplifies Statistical Significance Estimation PLoS Comput Biol 2008 4 e1000069 18516236 10.1371/journal.pcbi.1000069 PMC2396288

