# Supplementary material for: A new computational approach redefines the subtelomeric vir superfamily of Plasmodium vivax
Source: BMC Genomics. 2013 Jan 16;14:8. doi: 10.1186/1471-2164-14-8 (PMC3566924; doi:10.1186/1471-2164-14-8)
Supplement: Additional file 3 — Genome-wide distribution of newly predicted virgenes. Figure showing the chromosomal location of newly annotated hypothetical proteins as VIR proteins. For each chromosome, two rows of colored boxes are shown: one row illustrates the genomic location of the vir members (red) while the other illustrates the location of the putative vir (green) in that chromosome. Only genes annotated at specific chromosomes are shown. [file 1471-2164-14-8-S3.pdf]

vir chromosomal location

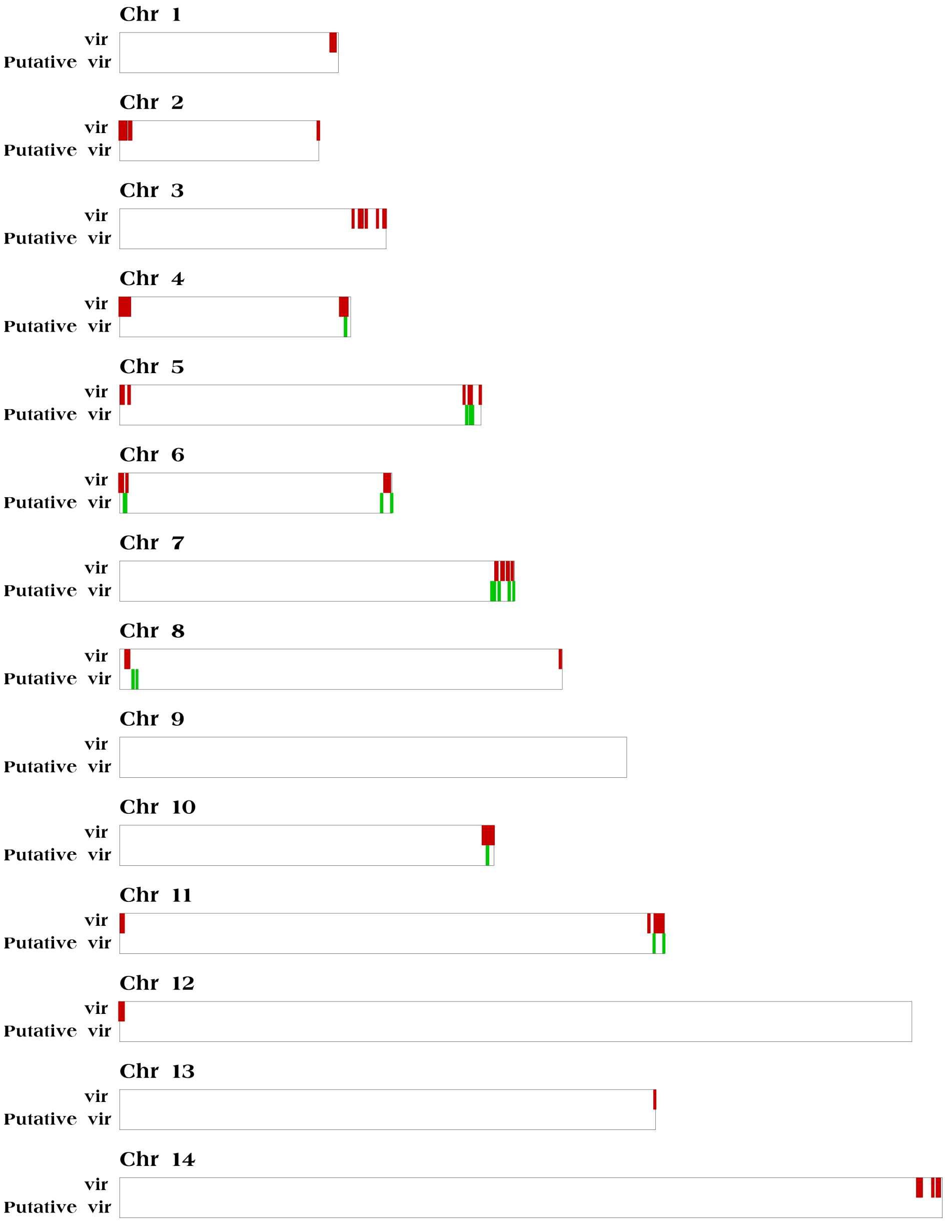

Description: For each chromosome, two rows of colored boxes are shown: one row illustrates the genomic location of the *vir* members (red) while the other illustrates the location of the putative *vir* (green) in that chromosome. Only genes annotated at specific chromosomes are shown.
