# Supplementary material for: A new computational approach redefines the subtelomeric vir superfamily of Plasmodium vivax
Source: BMC Genomics. 2013 Jan 16;14:8. doi: 10.1186/1471-2164-14-8 (PMC3566924; doi:10.1186/1471-2164-14-8)
Supplement: Additional file 6 — Conserved motifs composition. Composition of clusters and the conserved motif structure of each of the proteins in the original VIR set, as well as the hypothetical proteins grouped with them. An illustration of the most representative homology blocks in each family is also included as well as InterproScan predictions for newly defined (sub)families. [file 1471-2164-14-8-S6.pdf]

Conserved motifs composition

1 InterproScan predictions

Description: tables below provide InterproScan predictions for each of the different (sub)families.

1.1 B

#	Gene ID	Length	Match ID	Match Name	DB Name	Start	End	Score
1	PVX_033190	357	PF05795	Plasmodium_Vir	PFAM	23	353	9.4E-25
2	PVX_115480	276	PF05795	Plasmodium_Vir	PFAM	24	237	6.6e-23
			coil	coiled-coil	COIL	53	74	NA
3	PVX_025690	351	PF05795	Plasmodium_Vir	PFAM	24	348	2.8E-24
4	PVX_058690	299	PF05795	Plasmodium_Vir	PFAM	27	295	2.1E-22
5	PVX_062190	326	PF05795	Plasmodium_Vir	PFAM	1	322	8.6-17
6	PVX_164265	362	PF05795	Plasmodium_Vir	PFAM	23	358	1.6E-21
7	PVX_180275	236	PF05795	Plasmodium_Vir	PFAM	35	231	4.1E-17
8	PVX_103680	361	PF05795	Plasmodium_Vir	PFAM	24	357	8.2-27
9	PVX_108775	62	PF05795	Plasmodium_Vir	PFAM	1	57	9.5E-7
10	PVX_110305	267	PF05795	Plasmodium_Vir	PFAM	24	239	1.0E-24
11	PVX_088790	467	PF05795	Plasmodium_Vir	PFAM	28	206	9.6E-18
			PF05795	Plasmodium_Vir	PFAM	236	452	1.3E-23
12	PVX_096915	295	PF05795	Plasmodium_Vir	PFAM	62	268	5.5E-22
13	PVX_075195	302	PF05795	Plasmodium_Vir	PFAM	24	242	5.1E-16
14	PVX_086350	397	PF05795	Plasmodium_Vir	PFAM	25	396	3.7E-42
15	PVX_059690							

1.2 C

#	Gene ID	Length	Match ID	Match Name	DB Name	Start	End	Score
1	PVX_004520	363	PF05795	Plasmodium_Vir	PFAM	21	362	1.3E-65
2	PVX_043690	85	PF05795	Plasmodium_Vir	PFAM	6	85	1.2E-10
3	PVX_088800	358	PF05795	Plasmodium_Vir	PFAM	11	355	3.2E-67
4	PVX_169270	346	PF05795	Plasmodium_Vir	PFAM	21	346	2.2E-58
5	PVX_003485	329	PF05795	Plasmodium_Vir	PFAM	37	317	6.7E-54
6	PVX_004510	353	PF05795	Plasmodium_Vir	PFAM	9	326	2.7E-57
7	PVX_044190	61	PF05795	Plasmodium_Vir	PFAM	1	61	7.8E-8
8	PVX_045690	140	PF05795	Plasmodium_Vir	PFAM	21	137	9.1E-17
9	PVX_175270	332	PF05795	Plasmodium_Vir	PFAM	6	332	1.1E-60
10	PVX_179275	238	PF05795	Plasmodium_Vir	PFAM	15	215	4.6e-39
			SignalP-NN(euk)	signal-peptide	SIGNALP	1	12	NA
11	PVX_184285	348	PF05795	Plasmodium_Vir	PFAM	22	348	7.2E-71
12	PVX_105700	340	PF05795	Plasmodium_Vir	PFAM	21	340	2.5E-63
13	PVX_108770	409	PF05795	Plasmodium_Vir	PFAM	33	405	4.9E-48
14	PVX_113230	440	PF05795	Plasmodium_Vir	PFAM	10	375	3E-49
15	PVX_061190	342	PF05795	Plasmodium_Vir	PFAM	21	342	1.6E-60
16	PVX_066690	352	PF05795	Plasmodium_Vir	PFAM	121	351	1.6e-20
			seg	seg	SEG	286	300	NA
17	PVX_072690	324	PF05795	Plasmodium_Vir	PFAM	3	324	8.9E-48
18	PVX_029190	335	PF05795	Plasmodium_Vir	PFAM	21	335	7.9E-61
19	PVX_030190	325	PF05795	Plasmodium_Vir	PFAM	2	325	2E-58
20	PVX_034190	403	PF05795	Plasmodium_Vir	PFAM	34	400	1.8E-58
21	PVX_037190	404	PF05795	Plasmodium_Vir	PFAM	34	400	6.2E-55
22	PVX_021680	355	PF05795	Plasmodium_Vir	PFAM	23	355	7.3E-71
23	PVX_024690	343	PF05795	Plasmodium_Vir	PFAM	21	340	5.6E-43
24	PVX_026190	353	PF05795	Plasmodium_Vir	PFAM	20	353	2.8E-55
25	PVX_010610	315	PF05795	Plasmodium_Vir	PFAM	24	315	5.3E-50
26	PVX_016140	324	PF05795	Plasmodium_Vir	PFAM	24	324	1.2E-53
27	PVX_019670	172	PF05795	Plasmodium_Vir	PFAM	34	172	3.4E-21
28	PVX_127260	210	PF05795	Plasmodium_Vir	PFAM	21	204	6.8E-35
29	PVX_149260	270	PF05795	Plasmodium_Vir	PFAM	2	269	2.4E-54
30	PVX_159260	527	PF05795	Plasmodium_Vir	PFAM	160	527	8.1e-56
			seg	seg	SEG	69	81	NA
			SignalP-NN(euk)	signal-peptide	SIGNALP	1	22	NA
			tmhmm	transmembrane_regions	TMHMM	24	44	NA
			tmhmm	transmembrane_regions	TMHMM	71	89	NA
			tmhmm	transmembrane_regions	TMHMM	116	136	NA
31	PVX_115475	229	PF05795	Plasmodium_Vir	PFAM	14	212	2E-34
32	PVX_120330	418	PF05795	Plasmodium_Vir	PFAM	16	417	3.1E-56
33	PVX_124700	255	PF05795	Plasmodium_Vir	PFAM	24	219	3.2E-26
34	PVX_124710	313	PF05795	Plasmodium_Vir	PFAM	9	304	3.7E-51

35	PVX_103665	290	PF05795	Plasmodium_Vir	PFAM	8	289	9.5E-38
36	PVX_104185	288	PF05795	Plasmodium_Vir	PFAM	2	287	7.8E-24
37	PVX_101615	361	PF05795	Plasmodium_Vir	PFAM	9	319	1.7E-54
38	PVX_102125	153	PF05795	Plasmodium_Vir	PFAM	6	152	4.5E-16
39	PVX_103660	360	PF05795	Plasmodium_Vir	PFAM	34	348	3.4E-41
40	PVX_096945	314	PF05795	Plasmodium_Vir	PFAM	20	310	1.8E-31
41	PVX_101560	302	PF05795	Plasmodium_Vir	PFAM	6	201	3.5E-25
42	PVX_101600	372	PF05795	Plasmodium_Vir	PFAM	22	369	9.4E-48
43	PVX_095995	315	PF05795	Plasmodium_Vir	PFAM	18	310	2.7E-21
44	PVX_096000	353	PF05795	Plasmodium_Vir	PFAM	22	352	1.1E-58
45	PVX_027690	315	PF05795	Plasmodium_Vir	PFAM	15	315	2.3E-49
46	PVX_028190	284	PF05795	Plasmodium_Vir	PFAM	2	284	1.0E-48
47	PVX_004525	353	PF05795	Plasmodium_Vir	PFAM	20	352	1.4E-41
48	PVX_004535	330	PF05795	Plasmodium_Vir	PFAM	24	327	3.2E-31
49	PVX_006580	351	PF05795	Plasmodium_Vir	PFAM	23	351	1.0E-64
50	PVX_008085	418	PF05795	Plasmodium_Vir	PFAM	33	392	2.6E-54
51	PVX_009090	337	PF05795	Plasmodium_Vir	PFAM	10	337	8.4E-60
52	PVX_051690	334	PF05795	Plasmodium_Vir	PFAM	21	331	5.5E-60
53	PVX_052190	360	PF05795	Plasmodium_Vir	PFAM	146	288	3.5e-18
			seg	seg	SEG	1	12	NA
			tmhmm	transmembrane_regions	TMHMM	4	24	NA
			tmhmm	transmembrane_regions	TMHMM	258	278	NA
			tmhmm	transmembrane_regions	TMHMM	297	315	NA
54	PVX_052690	279	PF05795	Plasmodium_Vir	PFAM	2	276	5.3E-39
55	PVX_076695	325	PF05795	Plasmodium_Vir	PFAM	11	325	4.7E-46
56	PVX_086875	292	PF05795	Plasmodium_Vir	PFAM	13	286	1.2E-29
57	PVX_046190	320	PF05795	Plasmodium_Vir	PFAM	118	237	1.8e-13
			seg	seg	SEG	19	33	NA
			seg	seg	SEG	173	189	NA
			seg	seg	SEG	292	305	NA
			tmhmm	transmembrane_regions	TMHMM	213	233	NA
			tmhmm	transmembrane_regions	TMHMM	248	268	NA
			tmhmm	transmembrane_regions	TMHMM	291	309	NA
58	PVX_050190	340	PF05795	Plasmodium_Vir	PFAM	9	340	1.5E-71
59	PVX_005065	198	PF05795	Plasmodium_Vir	PFAM	18	175	2.2E-20
60	PVX_005580	297	PF05795	Plasmodium_Vir	PFAM	6	280	5.7E-53
61	PVX_090320	212	PF05795	Plasmodium_Vir	PFAM	21	210	3.1E-33
62	PVX_094260	198	PF05795	Plasmodium_Vir	PFAM	4	196	4.5E-20
63	PVX_005575							

1.3 E

#	Gene ID	Length	Match ID	Match Name	DB Name	Start	End	Score
1	PVX_001615	366	PF05795	Plasmodium_Vir	PFAM	18	361	1.2E-61
2	PVX_001645	529	PF05795	Plasmodium_Vir	PFAM	22	518	3.5E-46
3	PVX_003510	453	PF05795	Plasmodium_Vir	PFAM	18	356	3.6E-52
4	PVX_094240	443	PF05795	Plasmodium_Vir	PFAM	31	430	1.0E-75
5	PVX_101620	594	PF05795	Plasmodium_Vir	PFAM	22	588	6.7E-64
6	PVX_102640	441	PF05795	Plasmodium_Vir	PFAM	16	435	1.3E-95
7	PVX_107235	582	PF05795	Plasmodium_Vir	PFAM	16	284	7.4E-71
			PF05795	Plasmodium_Vir	PFAM	410	576	3.6E-28
8	PVX_107735	362	PF05795	Plasmodium_Vir	PFAM	6	332	9.0E-49
9	PVX_107740	304	PF05795	Plasmodium_Vir	PFAM	12	292	1.1E-48
10	PVX_093710	488	PF05795	Plasmodium_Vir	PFAM	21	448	2.5E-62
11	PVX_093720	496	PF05795	Plasmodium_Vir	PFAM	22	354	7.0E-61
			PF05795	Plasmodium_Vir	PFAM	418	494	1.5E-8
12	PVX_086860	447	PF05795	Plasmodium_Vir	PFAM	14	444	5.9E-64
13	PVX_086865	576	PF05795	Plasmodium_Vir	PFAM	67	308	1.2E-19
			PF05795	Plasmodium_Vir	PFAM	314	545	2.1E-49
14	PVX_086895	553	PF05795	Plasmodium_Vir	PFAM	21	246	1.7E-10
			PF05795	Plasmodium_Vir	PFAM	278	549	1.1E-46
15	PVX_088780	446	PF05795	Plasmodium_Vir	PFAM	20	437	2.5E-49
16	PVX_077695	545	PF05795	Plasmodium_Vir	PFAM	20	539	2.5E-87
17	PVX_078195	569	PF05795	Plasmodium_Vir	PFAM	16	229	3.1E-58
			PF05795	Plasmodium_Vir	PFAM	397	563	9.2E-27
18	PVX_073690	433	PF05795	Plasmodium_Vir	PFAM	22	430	9.2E-80
19	PVX_074190	323	PF05795	Plasmodium_Vir	PFAM	11	318	4.7E-53
20	PVX_074690	427	PF05795	Plasmodium_Vir	PFAM	22	421	1.9E-77
21	PVX_018660	493	PF05795	Plasmodium_Vir	PFAM	16	488	3.5E-60
22	PVX_020170	333	PF05795	Plasmodium_Vir	PFAM	1	328	2.9E-57
23	PVX_020680	581	PF05795	Plasmodium_Vir	PFAM	16	276	1.6E-69
			PF05795	Plasmodium_Vir	PFAM	409	575	3.8E-27
24	PVX_009595	392	PF05795	Plasmodium_Vir	PFAM	20	390	9.7E-72
25	PVX_010105	421	PF05795	Plasmodium_Vir	PFAM	16	416	3.6E-88
26	PVX_013620	537	PF05795	Plasmodium_Vir	PFAM	16	531	2.0E-86
27	PVX_002480	616	PF05795	Plasmodium_Vir	PFAM	26	231	2.2E-11
			PF05795	Plasmodium_Vir	PFAM	254	615	2.8E-82
28	PVX_002485	276	PF05795	Plasmodium_Vir	PFAM	17	234	4.0E-42
29	PVX_158260	287	PF05795	Plasmodium_Vir	PFAM	16	263	2.7E-65
30	PVX_170270	332	PF05795	Plasmodium_Vir	PFAM	16	276	3.2E-64
31	PVX_124712	400	PF05795	Plasmodium_Vir	PFAM	82	386	1.4E-20
32	PVX_124715	528	PF05795	Plasmodium_Vir	PFAM	248	461	5.6E-49
33	PVX_119210	540	PF05795	Plasmodium_Vir	PFAM	11	282	7.4E-14
34	PVX_120340	452	PF05795	Plasmodium_Vir	PFAM	17	446	2.6E-94
35	PVX_120845	587	PF05795	Plasmodium_Vir	PFAM	28	582	1.8E-67
36	PVX_112630	650	PF05795	Plasmodium_Vir	PFAM	34	649	8.1E-47
37	PVX_112720	505	PF05795	Plasmodium_Vir	PFAM	15	246	1.2E-37
38	PVX_115485	302	PF05795	Plasmodium_Vir	PFAM	21	300	1.8E-61
39	PVX_115985	574	PF05795	Plasmodium_Vir	PFAM	29	338	1.8E-52
			PF05795	Plasmodium_Vir	PFAM	399	568	6.9E-23
40	PVX_110295	553	PF05795	Plasmodium_Vir	PFAM	17	552	9.0E-55
41	PVX_110300	359	PF05795	Plasmodium_Vir	PFAM	26	359	5.4E-75
42	PVX_104190	426	PF05795	Plasmodium_Vir	PFAM	16	426	1.9E-67
43	PVX_106220	481	PF05795	Plasmodium_Vir	PFAM	21	281	5.0E-70
			PF05795	Plasmodium_Vir	PFAM	385	474	1.2E-8
44	PVX_097540	668	PF05795	Plasmodium_Vir	PFAM	321	635	2.1E-77
45	PVX_097542	560	PF05795	Plasmodium_Vir	PFAM	244	558	2.6E-60
46	PVX_097545	451	PF05795	Plasmodium_Vir	PFAM	7	207	2.4E-30
47	PVX_097555	516	PF05795	Plasmodium_Vir	PFAM	16	225	9.3E-38
48	PVX_097525	508	PF05795	Plasmodium_Vir	PFAM	16	502	3.3E-92
49	PVX_097530	524	PF05795	Plasmodium_Vir	PFAM	36	352	1.6E-30
50	PVX_090315	487	PF05795	Plasmodium_Vir	PFAM	51	481	5.01E-82
51	PVX_090335	435	PF05795	Plasmodium_Vir	PFAM	19	427	9.1E-77

52	PVX_023185	476	PF05795	Plasmodium_Vir	PFAM	19	471	1.7E-104
53	PVX_024685	419	PF05795	Plasmodium_Vir	PFAM	2	413	3.8E-82
54	PVX_027190	200	PF05795	Plasmodium_Vir	PFAM	19	194	3.9E-30
55	PVX_004495	449	PF05795	Plasmodium_Vir	PFAM	6	443	5.3E-46
56	PVX_004537	466	PF05795	Plasmodium_Vir	PFAM	16	371	1.3E-54
57	PVX_005045	445	PF05795	Plasmodium_Vir	PFAM	22	249	3.5E-54
58	PVX_003495	635	PF05795	Plasmodium_Vir	PFAM	7	229	8.0E-11
			PF05795	Plasmodium_Vir	PFAM	253	609	2.9E-87
59	PVX_003505	414	PF05795	Plasmodium_Vir	PFAM	140	358	1.1E-51
60	PVX_081850	608	PF05795	Plasmodium_Vir	PFAM	29	262	6.6E-14
			PF05795	Plasmodium_Vir	PFAM	288	608	6.0E-55
61	PVX_083575	510	PF05795	Plasmodium_Vir	PFAM	11	462	1.5E-64
62	PVX_083580	575	PF05795	Plasmodium_Vir	PFAM	23	235	2.0E-9
			PF05795	Plasmodium_Vir	PFAM	281	568	1.1E-63
63	PVX_083590	452	PF05795	Plasmodium_Vir	PFAM	23	452	2.1E-97
64	PVX_042190	450	PF05795	Plasmodium_Vir	PFAM	2	445	2.8E-92
65	PVX_049690	366	PF05795	Plasmodium_Vir	PFAM	124	360	2.6E-17
66	PVX_054190	396	PF05795	Plasmodium_Vir	PFAM	4	391	1.2E-86
67	PVX_061690	273	PF05795	Plasmodium_Vir	PFAM	28	268	3.3E-33
68	PVX_015635	529	PF05795	Plasmodium_Vir	PFAM	16	277	3.7E-71
			PF05795	Plasmodium_Vir	PFAM	411	528	9.5E-9
69	PVX_017140	568	PF05795	Plasmodium_Vir	PFAM	16	292	2.8E-69
			PF05795	Plasmodium_Vir	PFAM	386	562	2.4E-28
70	PVX_101565	473	PF05795	Plasmodium_Vir	PFAM	22	419	1.7E-57
71	PVX_106725	469	PF05795	Plasmodium_Vir	PFAM	18	257	5.2E-46
72	PVX_124705	449	PF05795	Plasmodium_Vir	PFAM	16	443	3.4E-89
73	PVX_103155	442	PF05795	Plasmodium_Vir	PFAM	22	436	2.1E-80
74	PVX_104180	386	PF05795	Plasmodium_Vir	PFAM	27	243	1.8E-29
75	PVX_094245	584	PF05795	Plasmodium_Vir	PFAM	18	578	3.5E-70
76	PVX_094250	525	PF05795	Plasmodium_Vir	PFAM	10	274	9.0E-16
77	PVX_090290	288	PF05795	Plasmodium_Vir	PFAM	2	280	2.2E-52
78	PVX_090295	542	PF05795	Plasmodium_Vir	PFAM	24	239	3.8E-11
			PF05795	Plasmodium_Vir	PFAM	269	536	6.4E-52
79	PVX_090305	462	PF05795	Plasmodium_Vir	PFAM	16	457	1.6E-63
80	PVX_090310	449	PF05795	Plasmodium_Vir	PFAM	2	210	6.42E-34
81	PVX_172270	378	PF05795	Plasmodium_Vir	PFAM	10	375	1.7E-64
82	PVX_183280	443	PF05795	Plasmodium_Vir	PFAM	2	437	2.9E-43
83	PVX_241290	771	PF05795	Plasmodium_Vir	PFAM	9	185	5.4E-6
			PF05795	Plasmodium_Vir	PFAM	203	319	5.0E-21
			PF05795	Plasmodium_Vir	PFAM	433	625	1.6E-23
84	PVX_133260	268	PF05795	Plasmodium_Vir	PFAM	17	259	3.5E-62
85	PVX_134260	104	PF05795	Plasmodium_Vir	PFAM	17	103	9.0E-21
86	PVX_157260	315	PF05795	Plasmodium_Vir	PFAM	3	173	1.5E-43
87	PVX_096910	506	PF05795	Plasmodium_Vir	PFAM	21	163	4.4E-11
			PF05795	Plasmodium_Vir	PFAM	174	464	7.4E-63
88	PVX_096920	491	PF05795	Plasmodium_Vir	PFAM	16	308	7.4E-32
89	PVX_088785	314	PF05795	Plasmodium_Vir	PFAM	6	312	1.6E-57
90	PVX_088805	358	PF05795	Plasmodium_Vir	PFAM	17	358	8.45E-76
91	PVX_107745	351	PF05795	Plasmodium_Vir	PFAM	19	351	2.2E-58
92	PVX_107755	657	PF05795	Plasmodium_Vir	PFAM	68	279	2.6E-6
			PF05795	Plasmodium_Vir	PFAM	290	656	4.0E-94
93	PVX_063190	483	PF05795	Plasmodium_Vir	PFAM	22	478	2.6E-103
94	PVX_065690	394	PF05795	Plasmodium_Vir	PFAM	10	389	1.1E-64
95	PVX_037690	67	PF05795	Plasmodium_Vir	PFAM	1	62	1.1E-17
96	PVX_038190	58	PF05795	Plasmodium_Vir	PFAM	4	53	3.8E-11
97	PVX_041690	379	PF05795	Plasmodium_Vir	PFAM	7	372	1.6E-60
98	PVX_034690	404	PF05795	Plasmodium_Vir	PFAM	5	63	1.4E-9
			PF05795	Plasmodium_Vir	PFAM	75	399	1.2E-53
99	PVX_036690	98	PF05795	Plasmodium_Vir	PFAM	7	93	1.8E-20
100	PVX_022185	352	PF05795	Plasmodium_Vir	PFAM	13	348	4.6E-55
101	PVX_022685	630	PF05795	Plasmodium_Vir	PFAM	16	624	1.7E-83
102	PVX_005055	534	PF05795	Plasmodium_Vir	PFAM	25	522	3.5E-44
103	PVX_005060	451	PF05795	Plasmodium_Vir	PFAM	16	391	4.0E-79
104	PVX_007085	321	PF05795	Plasmodium_Vir	PFAM	3	320	2.2E-68
105	PVX_002490	464	PF05795	Plasmodium_Vir	PFAM	13	452	8.9E-38
106	PVX_003490	612	PF05795	Plasmodium_Vir	PFAM	24	217	7.2E-14
			PF05795	Plasmodium_Vir	PFAM	410	598	1.6E-38
107	PVX_001625	460	PF05795	Plasmodium_Vir	PFAM	16	452	5.2E-97
108	PVX_001635	634	PF05795	Plasmodium_Vir	PFAM	24	267	2.5E-28
			PF05795	Plasmodium_Vir	PFAM	278	623	1.0E-58
109	PVX_001640	227	PF05795	Plasmodium_Vir	PFAM	16	178	6.2E-37
110	PVX_124720	545	PF05795	Plasmodium_Vir	PFAM	19	241	6.5E-43
111	PVX_124725	220	PF05795	Plasmodium_Vir	PFAM	1	182	2.5E-34
112	PVX_125726	364	PF05795	Plasmodium_Vir	PFAM	2	359	6.8E-31
113	PVX_075695	436	PF05795	Plasmodium_Vir	PFAM	22	431	3.2E-78
114	PVX_076195	459	PF05795	Plasmodium_Vir	PFAM	17	446	1.3E-94

1.4 G

#	Gene ID	Length	Match ID	Match Name	DB Name	Start	End	Score
1	PVX_024185	427	PF05795	Plasmodium_Vir	PFAM	18	289	3.6E-14
2	PVX_035190	410	PF05795	Plasmodium_Vir	PFAM	7	179	6.1E-7
3	PVX_079195	427	PF05795	Plasmodium_Vir	PFAM	35	390	9.5E-14
4	PVX_007080	451	PF05795	Plasmodium_Vir	PFAM	13	451	3.4E-16
5	PVX_039190	438	PF05795	Plasmodium_Vir	PFAM	13	422	2.1E-19
6	PVX_048690	408	PF05795	Plasmodium_Vir	PFAM	17	408	3.3E-41
7	PVX_007585	447	PF05795	Plasmodium_Vir	PFAM	32	446	4.0E-13
8	PVX_012115	506	PF05795	Plasmodium_Vir	PFAM	32	501	1.9E-13
9	PVX_014125	498	coil	coiled-coil	COIL	259	307	NA
			seg	seg	SEG	76	86	NA
			seg	seg	SEG	92	106	NA
			seg	seg	SEG	217	234	NA
			seg	seg	SEG	253	305	NA
			seg	seg	SEG	333	344	NA
			seg	seg	SEG	411	423	NA
			tmhmm	transmembrane_regions	TMHMM	21	39	NA
			tmhmm	transmembrane_regions	TMHMM	45	67	NA
			tmhmm	transmembrane_regions	TMHMM	101	121	NA
			tmhmm	transmembrane_regions	TMHMM	436	456	NA
10	PVX_102130	456	PF05795	Plasmodium_Vir	PFAM	60	253	3.0E-10
11	PVX_103670	459	PF05795	Plasmodium_Vir	PFAM	48	449	1.4E-6
12	PVX_106210	434	PF05795	Plasmodium_Vir	PFAM	12	215	7.8E-7
13	PVX_108255	442	PF05795	Plasmodium_Vir	PFAM	18	409	1.7E-14

14	PVX_059190	438	PF05795	Plasmodium_Vir	PFAM	13	408	1.1E-16
15	PVX_063690	456	PF05795	Plasmodium_Vir	PFAM	34	440	7.1E-19
16	PVX_028690	464	PF05795	Plasmodium_Vir	PFAM	53	431	3.5E-8
17	PVX_032190	497	PF05795	Plasmodium_Vir	PFAM	76	492	2.3E-22
18	PVX_014630	464	PF05795	Plasmodium_Vir	PFAM	20	430	6.3E-8
19	PVX_018655	515	PF05795	Plasmodium_Vir	PFAM	34	510	5.0E-19
20	PVX_112115	320	PF05795	Plasmodium_Vir	PFAM	20	313	2.2E-9
21	PVX_121345	449	PF05795	Plasmodium_Vir	PFAM	15	443	1.0E-14
22	PVX_174270	458	seg	seg	SEG	149	165	NA
			seg	seg	SEG	219	230	NA
			seg	seg	SEG	384	399	NA
			seg	seg	SEG	406	417	NA
			tmhmm	transmembrane_regions	TMHMM	155	175	NA
			tmhmm	transmembrane_regions	TMHMM	189	207	NA
			tmhmm	transmembrane_regions	TMHMM	353	371	NA
			tmhmm	transmembrane_regions	TMHMM	375	395	NA
23	PVX_050690							
24	PVX_047190							
25	PVX_071190							
26	PVX_026690							
27	PVX_033690							
28	PVX_073190							
29	PVX_031190							
30	PVX_030690							
31	PVX_029690							
32	PVX_070190							
33	PVX_104695							
34	PVX_057190							
35	PVX_056190							
36	PVX_040190							
37	PVX_060190							
38	PVX_102645							

1.5 I

#	Gene ID	Length	Match ID	Match Name	DB Name	Start	End	Score
1	7077_PVX_014625	375	PF05795	Plasmodium_Vir	PFAM	13	362	2.7E-40
2	7077_PVX_064190	364	PF05795	Plasmodium_Vir	PFAM	13	363	4.6E-42
3	7077_PVX_002495	324	PF05795	Plasmodium_Vir	PFAM	18	310	1.5E-40
4	7077_PVX_023685	292	PF05795	Plasmodium_Vir	PFAM	2	291	4.2e-31
5	7077_PVX_032690	156	PF05795	Plasmodium_Vir	PFAM	20	155	8.1E-12
6	7077_PVX_072190	362	PF05795	Plasmodium_Vir	PFAM	13	361	2.5E-41
7	7077_PVX_102630	345	PF05795	Plasmodium_Vir	PFAM	44	343	3.6e-23
			SignalP-NN(euk)	signal-peptide	SIGNALP	1	22	NA
8	7077_PVX_064690	363	PF05795	Plasmodium_Vir	PFAM	13	361	6.0E-40
9	7077_PVX_069190	343	PF05795	Plasmodium_Vir	PFAM	31	343	1.4E-29
10	7077_PVX_048190	117	PF05795	Plasmodium_Vir	PFAM	13	111	6.1E-10
11	7077_PVX_053190	344	PF05795	Plasmodium_Vir	PFAM	16	342	2.5E-35
12	7077_PVX_055690	192	PF05795	Plasmodium_Vir	PFAM	3	190	1.7E-8
13	7077_PVX_150260	361	PF05795	Plasmodium_Vir	PFAM	17	360	2.4E-37
14	7077_PVX_177275	328	PF05795	Plasmodium_Vir	PFAM	11	326	4.2E-31
15	7077_PVX_248300	396	PF05795	Plasmodium_Vir	PFAM	16	396	1.4e-40
			seg	seg	SEG	284	300	NA
			seg	seg	SEG	333	344	NA
			tmhmm	transmembrane_regions	TMHMM	308	328	NA
			tmhmm	transmembrane_regions	TMHMM	333	353	NA
16	PVX_054690							
17	PVX_039690							

1.6 J

#	Gene ID	Length	Match ID	Match Name	DB Name	Start	End	Score
1	PVX_010605	286	PF05795	Plasmodium_Vir	PFAM	16	187	1.1E-8
			PF05795	Plasmodium_Vir	PFAM	200	283	1.2E-8
2	PVX_107750	301	PF05795	Plasmodium_Vir	PFAM	19	210	6.6E-22
			PF05795	Plasmodium_Vir	PFAM	214	297	2.0E-8
3	PVX_000020	234	PF05795	Plasmodium_Vir	PFAM	22	182	1.3E-10
4	PVX_057690	301	PF05795	Plasmodium_Vir	PFAM	22	201	6.9E-7
5	PVX_067690	266	PF05795	Plasmodium_Vir	PFAM	28	202	1.3E-7
6	PVX_109795	301	PF05795	Plasmodium_Vir	PFAM	19	199	6.6E-10
			PF05795	Plasmodium_Vir	PFAM	224	298	2.7E-6
7	PVX_167265	278	PF05795	Plasmodium_Vir	PFAM	10	193	2.1E-11
			PF05795	Plasmodium_Vir	PFAM	201	277	4.9E-8
8	PVX_168270	334	PF05795	Plasmodium_Vir	PFAM	16	330	3.3e-08
			seg	seg	SEG	277	288	NA
			tmhmm	transmembrane_regions	TMHMM	240	260	NA
			tmhmm	transmembrane_regions	TMHMM	270	288	NA
9	PVX_185285	343	PF05795	Plasmodium_Vir	PFAM	58	224	1.3e-06
			seg	seg	SEG	2	12	NA
			seg	seg	SEG	231	251	NA
			seg	seg	SEG	255	264	NA
			SignalP-NN(euk)	signal-peptide	SIGNALP	1	18	NA
			tmhmm	transmembrane_regions	TMHMM	4	24	NA
			tmhmm	transmembrane_regions	TMHMM	232	252	NA
			tmhmm	transmembrane_regions	TMHMM	280	300	NA
10	PVX_096935	304	PF05795	Plasmodium_Vir	PFAM	38	302	5.7E-19
11	PVX_105705	191	PF05795	Plasmodium_Vir	PFAM	23	185	3.4E-13
12	PVX_105710	245	PF05795	Plasmodium_Vir	PFAM	28	202	1.1E-11
13	PVX_070690	260	PF05795	Plasmodium_Vir	PFAM	3	165	7.4E-10
14	PVX_096005	367	PF05795	Plasmodium_Vir	PFAM	56	333	8.5E-9
15	PVX_096925	287	seg	seg	SEG	194	217	NA
			tmhmm	transmembrane_regions	TMHMM	196	218	NA
			tmhmm	transmembrane_regions	TMHMM	247	267	NA
16	PVX_096930	248	PF05795	Plasmodium_Vir	PFAM	18	199	5.5E-8
17	PVX_121355							
18	PVX_106720							
19	PVX_186290							

1.7 K

#	Gene ID	Length	Match ID	Match Name	DB Name	Start	End	Score
1	PVX_001610	338	PF05795	Plasmodium_Vir	PFAM	10	336	6.6E-16
2	PVX_042690	337	PF05795	Plasmodium_Vir	PFAM	35	336	2.6E-10
3	PVX_086885	266	PF05795	Plasmodium_Vir	PFAM	11	220	2.3E-8
4	PVX_115990	271	PF05795	Plasmodium_Vir	PFAM	42	243	1.5E-11
5	PVX_145260	305	PF05795	Plasmodium_Vir	PFAM	23	304	4.6E-6
6	PVX_163265	341	seg	seg	SEG	246	258	NA
7	PVX_056690							
8	PVX_053690							
9	PVX_031690							
10	PVX_005565							

1.8 PvPIRA

#	Gene ID	Length	Match ID	Match Name	DB Name	Start	End	Score
1	PVX_011610	362	PF05795	Plasmodium_Vir	PFAM	26	359	1.6E-9
2	PVX_141260	385	PF05795	Plasmodium_Vir	PFAM	11	382	7.7E-9
3	PVX_144260	403	seg	seg	SEG	27	48	NA
			seg	seg	SEG	165	177	NA
			seg	seg	SEG	318	336	NA
			SignalP-NN(euk)	signal-peptide	SIGNALP	1	38	NA
			tmhmm	transmembrane_regions	TMHMM	19	41	NA
			tmhmm	transmembrane_regions	TMHMM	130	150	NA
			tmhmm	transmembrane_regions	TMHMM	181	201	NA
			tmhmm	transmembrane_regions	TMHMM	302	320	NA
			tmhmm	transmembrane_regions	TMHMM	339	359	NA
4	PVX_155260	359	seg	seg	SEG	265	276	NA
			seg	seg	SEG	283	296	NA
			seg	seg	SEG	299	310	NA
			seg	seg	SEG	338	350	NA
5	PVX_015135	383	PF05795	Plasmodium_Vir	PFAM	20	380	4.2E-15
6	PVX_019160	394	PF05795	Plasmodium_Vir	PFAM	2	385	1.5E-15
7	PVX_139260	359	seg	seg	SEG	281	292	NA
			seg	seg	SEG	298	311	NA
			tmhmm	transmembrane_regions	TMHMM	253	271	NA
			tmhmm	transmembrane_regions	TMHMM	281	303	NA
8	PVX_186285	305	seg	seg	SEG	178	189	NA
			seg	seg	SEG	286	298	NA
			SignalP-NN(euk)	signal-peptide	SIGNALP	1	17	NA
			tmhmm	transmembrane_regions	TMHMM	279	297	NA
9	PVX_171270	244	seg	seg	SEG	211	218	NA
10	PVX_181275	470	seg	seg	SEG	17	35	NA
			seg	seg	SEG	430	449	NA
11	PVX_161260							
12	PVX_160260							
13	PVX_112645							
14	PVX_018155							
15	PVX_153260							

1.9 PvPIRD

#	Gene ID	Length	Match ID	Match Name	DB Name	Start	End	Score
1	PVX_038690	199	PF12420	DUF3671	PFAM	99	171	1.4E-18
2	PVX_047690	267	PF12420	DUF3671	PFAM	128	242	9.5E-35
3	PVX_074695	257	PF12420	DUF3671	PFAM	115	237	1.3E-29
4	PVX_102635	198	PF12420	DUF3671	PFAM	79	196	1.5E-24
5	PVX_103160	254	PF12420	DUF3671	PFAM	116	230	4.3E-29
6	PVX_017645	230	PF12420	DUF3671	PFAM	94	208	1.9E-28
7	PVX_019665	282	PF12420	DUF3671	PFAM	123	260	1.8E-37
8	PVX_108760	261	PF12420	DUF3671	PFAM	114	253	1.0E-34
9	PVX_109778	240	PF12420	DUF3671	PFAM	123	232	9.1E-19
10	PVX_109780	266	PF12420	DUF3671	PFAM	106	244	5.6E-37
11	PVX_146260	239	PF12420	DUF3671	PFAM	122	190	4.5E-26
12	PVX_178275	267	PF12420	DUF3671	PFAM	122	240	1.7E-32
13	PVX_182275	151	PF12420	DUF3671	PFAM	10	129	3.4E-29
14	PVX_103145	168	PF12420	DUF3671	PFAM	94	143	9.3E-11
15	PVX_086850	245	PF12420	DUF3671	PFAM	105	220	2.3E-33
16	PVX_058190	123	PF12420	DUF3671	PFAM	24	99	2E-20
17	PVX_067190	235	PF12420	DUF3671	PFAM	78	213	1.2E-34
18	PVX_040690	166	PF12420	DUF3671	PFAM	115	165	2.5E-13
19	PVX_041190	144	PF12420	DUF3671	PFAM	6	119	3.3E-37
20	PVX_109785	261	PF12420	DUF3671	PFAM	123	236	2.8E-38
21	PVX_143260	248	PF12420	DUF3671	PFAM	112	226	5.6E-30

1.10 PvPIRH

NO MATCHES

2 Homology Blocks summary

Description: illustrative summary of the representative HBs in each (sub)family. In order to create this figure, *representative* HBs were considered to be those present in at least the 50% of the sequences in the (sub)family. In other words, for each (sub)family, those HBs that appear in at least the 50% of sequences were selected and printed in the figure. Note that this does **not** mean that these *representative* HBs are specific of the (sub)family. In addition, PFAM domains which yielded the highest similarity to these HBs are indicated. Most of them correspond to a "*Plasmodium* Vir domain" entry in PFAM and domains of unknown function. Finally, note that this is just an illustration which intends to reflect the main similarities/differences among the (sub)families. Boxes representing the same motif appear aligned and filled with the same color. Further details can be found in Additional files 4, 5 and in the table below.

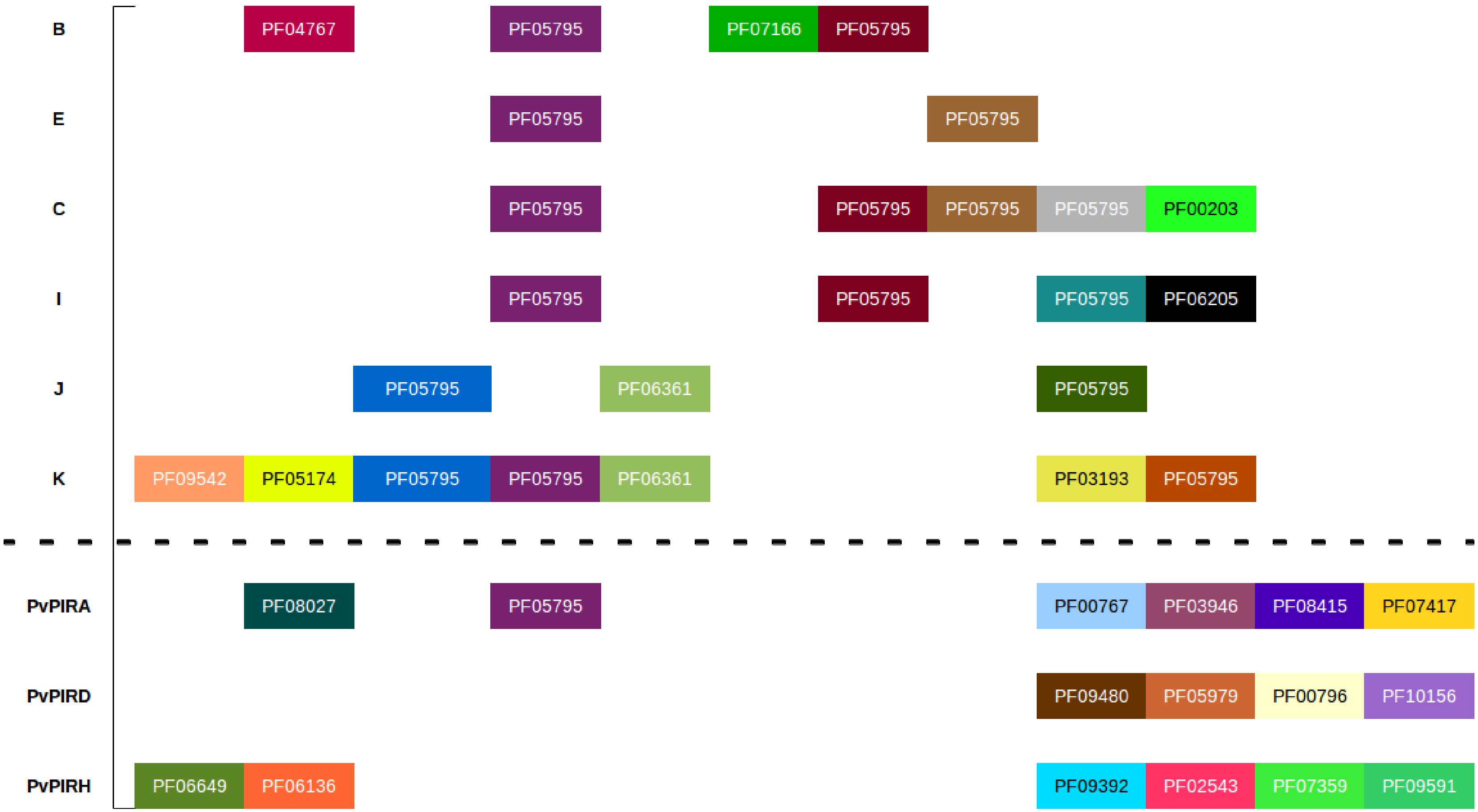
B
PF04767
PF05795
PF07166
PF05795
E
PF05795
PF05795
C
PF05795
PF05795
PF05795
PF05795
PF00203
I
PF05795
PF05795
PF05795
PF06205
J
PF05795
PF06361
PF05795
K
PF09542
PF05174
PF05795
PF05795
PF06361
PF03193
PF05795
PvPIRA
PF08027
PF05795
PF00767
PF03946
PF08415
PF07417
PvPIRD
PF09480
PF05979
PF00796
PF10156
PvPIRH
PF06649
PF06136
PF09392
PF02543
PF07359
PF09591

3 Homology Blocks detailed view

Description: composition of clusters and the conserved motif structure of each of the proteins in the original VIR set, as well as the hypothetical proteins grouped with them. A table is shown with the following columns:

- #: counter. Colors in this column indicate the membership to a certain cluster.
- Subfam.: indicates the original subfamily to which the corresponding gene was associated.
- Gene ID: PlasmoDB identifier.
- Name: annotation obtained from PlasmoDB 7.2.
- Length: number of aa.
- Graphic: represents the HB structure of the protein. Colored boxes indicate the presence of an HB. The thicker the box, the higher is the number of proteins found containing the corresponding HB.
- HB color: each row in this column corresponds to one of the HBs painted in the "Graphic" column.
- Starts, Ends: coordinates (in aa) that indicate the exact position of the HB in the protein. Note that HBs may overlap. Moreover, some of them may seem to be repeated, but actually represent HBs that present slight differences among them.
- Score: the number of proteins found to contain the corresponding HB.

#	Subfam.	Gene ID	Name	Length	Graphic	HB color	Starts	Ends	Score
1	E	PVX_074690	variable surface protein Vir4-related	427	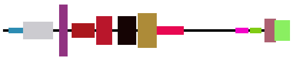		140	162	39
							10	29	7
							405	427	30
							389	403	32
							389	404	32
							347	365	5
							30	74	21
							389	403	32
							389	403	32
							405	427	30
							171	197	40
							175	197	40
							389	403	32
							389	403	32
							348	365	5
							202	224	46
							227	267	9
							202	228	46
							369	384	5
							84	95	72
							103	135	18
							84	94	72
2	E	PVX_073690	variable surface protein Vir12-related	433	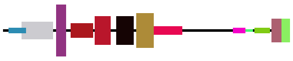		140	162	39
							10	29	7
							422	433	30
							406	420	32
							406	421	32
							347	365	5
							83	95	72
							35	74	21
							30	74	21
							406	420	32
							406	420	32
							422	433	30
							172	197	40
							175	197	40
							406	420	32
							406	420	32
							348	365	5
							204	224	46
							366	375	4
							227	270	9
							16	34	7

								202	226	46
								381	401	5
								82	95	72
								103	136	18
								84	94	72
	3	E	PVX_103155	variable surface protein Vir12-related	442	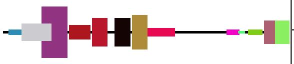		139	161	39
								10	29	7
								420	442	30
								404	418	32
								404	419	32
								345	363	5
								83	98	72
								60	98	72
								30	75	21
								404	418	32
								404	418	32
								420	442	30
								174	195	40
								404	418	32
								404	418	32
								346	363	5
								201	222	46
								364	373	4
								225	264	9
								82	97	72
								379	399	5
								83	98	72
								102	134	18
								84	95	72
	4	E	PVX_090335	variable surface protein Vir12-like	435	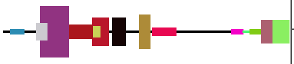		137	159	39
								119	146	13
								411	435	30
								167	187	40
								393	408	32
								393	408	32
								346	364	5
								79	95	72
								57	98	72
								393	408	32
								393	408	32
								411	435	30
								52	66	21
								393	408	32
								393	408	32
								348	364	5

						207	224	46
						365	374	4
						227	262	9
						81	94	72
						13	32	7
						376	390	5
						78	95	72
						101	135	18
						81	92	72
						173	186	40
5	L	PVX_024685	variable surface protein Vir12-related	419	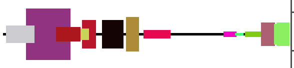	116	135	39
						91	125	13
						397	419	30
						379	395	32
						379	396	32
						323	340	5
						59	74	72
						181	197	46
						181	197	46
						35	98	72
						5	46	21
						379	395	32
						379	395	32
						397	419	30
						146	174	40
						30	43	21
						379	395	32
						401	419	30
						379	395	32
						324	340	5
						341	350	4
						206	245	9
						58	73	72
						356	376	5
						57	74	72
						79	112	18
						60	71	72
6	C	PVX_124705	variable surface protein Vir12-related	449	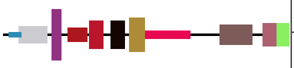	173	189	40
						135	156	39
						339	390	30
						407	426	32
						407	428	32
						25	69	21
						407	426	32
						408	426	32

							51	59	21
							409	426	32
							430	449	30
							409	426	32
							198	222	46
							223	293	9
							10	28	7
							78	92	72
							103	131	18
							78	89	72
							170	189	40
7	E	PVX_090315	variable surface protein Vir12-like	487	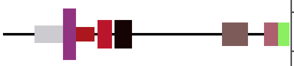		163	184	39
							373	416	30
							190	213	40
							445	464	32
							445	466	32
							106	122	72
							122	155	18
							56	101	21
							445	464	32
							446	464	32
							196	218	40
							198	218	40
							447	464	32
							468	487	30
							447	464	32
							108	121	72
							104	122	72
							127	155	18
							108	119	72
8		PVX_038190	hypothetical protein	58	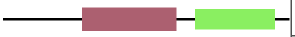		16	35	32
							16	35	32
							16	35	32
							17	35	32
							18	35	32
							39	55	30
							18	35	32
9	E	PVX_005060	variable surface protein Vir12-like	451	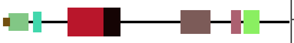		163	183	40
							135	152	39
							49	60	28
							280	326	30
							10	25	23
							13	40	23
							102	163	39
							359	373	32

							359	373	32
							359	373	32
							359	373	32
							159	182	40
							359	373	32
							380	393	30
							395	402	30
							359	373	32
							1	9	9
							1	11	9
							164	184	40
10	E	PVX_042190	variable surface protein Vir12-related, truncated	450	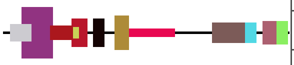		382	396	27
							382	396	27
							382	396	27
							110	132	39
							329	379	30
							382	397	27
							86	119	13
							382	396	27
							382	397	27
							143	159	40
							408	427	32
							408	429	32
							31	78	72
							13	44	21
							408	427	32
							409	427	32
							382	396	27
							26	39	21
							410	427	32
							431	447	30
							410	427	32
							382	396	27
							382	397	27
							199	269	9
							55	67	72
							177	197	46
							55	67	72
							75	108	18
							55	66	72
11	E	PVX_023185	variable surface protein Vir12-related	476	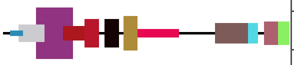		408	422	27
							408	422	27
							408	422	27
							136	158	39
							354	405	30

							408	423	27
							408	422	27
							408	423	27
							169	186	40
							434	453	32
							434	455	32
							202	217	46
							201	217	46
							57	116	72
							27	68	21
							434	453	32
							435	453	32
							408	422	27
							52	66	21
							436	453	32
							457	476	30
							436	453	32
							408	422	27
							408	423	27
							224	292	9
							81	93	72
							13	32	7
							202	222	46
							78	94	72
							100	134	18
							81	92	72
							172	191	40
12	L	PVX_063190	variable surface protein Vir12-related	483	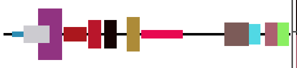		415	429	27
							415	429	27
							415	429	27
							143	163	39
							374	412	30
							415	430	27
							415	429	27
							415	430	27
							171	190	40
							441	460	32
							85	92	72
							441	462	32
							83	96	72
							103	127	18
							208	225	46
							60	98	72
							36	75	21
							441	460	32

							442	460	32
							415	429	27
							443	460	32
							463	480	30
							443	460	32
							415	429	27
							415	430	27
							232	301	9
							85	97	72
							16	33	7
							209	229	46
							82	98	72
							105	139	18
							85	96	72
13	L	PVX_010105	variable surface protein Vir12-related	421	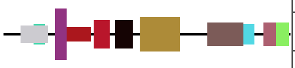		353	367	27
							353	367	27
							353	367	27
							78	92	72
							133	155	39
							300	350	30
							353	367	27
							353	367	27
							353	367	27
							46	59	28
							382	398	32
							382	398	32
							92	125	18
							26	65	21
							382	398	32
							382	398	32
							353	367	27
							166	188	40
							382	398	32
							402	418	30
							382	398	32
							353	367	27
							353	367	27
							77	90	72
							200	258	46
							202	222	46
							77	92	72
							98	127	18
							78	89	72
14	L	PVX_054190	variable surface protein Vir14-	396	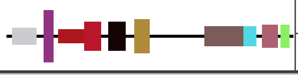		328	342	27
							328	342	27

			related, truncated				328	342	27
							108	130	39
							275	325	30
							328	343	27
							328	342	27
							328	343	27
							354	373	32
							354	373	32
							52	64	72
							8	40	21
							354	373	32
							355	373	32
							328	342	27
							141	163	40
							356	373	32
							379	390	30
							356	373	32
							328	342	27
							181	197	46
							328	343	27
							177	197	46
							52	65	72
							72	106	18
							53	64	72
15	E	PVX_120340	variable surface protein Vir12-related	452	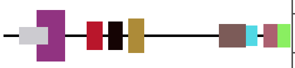		384	398	27
							384	398	27
							384	398	27
							133	155	39
							341	381	30
							384	399	27
							384	398	27
							384	399	27
							410	429	32
							410	431	32
							75	91	72
							53	97	72
							26	68	21
							410	429	32
							411	429	32
							384	398	27
							167	187	40
							50	62	21
							412	429	32
							433	452	30
							412	429	32

						384	398	27
						197	221	46
						384	399	27
						74	91	72
						77	88	72
16	E	PVX_076195	variable surface protein Vir12-related	459	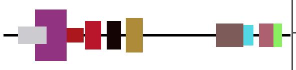	384	398	27
						384	398	27
						384	398	27
						133	155	39
						341	381	30
						384	393	27
						384	398	27
						384	393	27
						410	429	32
						410	431	32
						75	90	72
						52	100	72
						25	67	21
						410	429	32
						411	429	32
						384	398	27
						167	187	40
						49	61	21
						412	429	32
						433	445	30
						412	429	32
						384	398	27
						197	221	46
						384	393	27
						74	89	72
						75	90	72
						101	128	18
						76	87	72
17	E	PVX_075695	variable surface protein Vir12/22/24-related	436	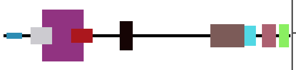	368	382	27
						368	382	27
						368	382	27
						6	28	7
						316	365	30
						368	383	27
						368	382	27
						368	383	27
						394	413	32
						394	413	32
						59	122	72
						43	74	21

							394	413	32
							395	413	32
							368	382	27
							177	196	40
							56	68	21
							396	413	32
							419	433	30
							396	413	32
							368	382	27
							368	383	27
							82	97	72
							83	98	72
							104	133	18
							84	95	72
18	L	PVX_083590	variable surface protein Vir12, putative	452	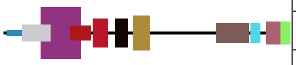		389	403	27
							389	403	27
							389	403	27
							141	163	39
							6	28	7
							335	386	30
							389	404	27
							389	403	27
							389	404	27
							415	434	32
							415	434	32
							59	121	72
							30	73	21
							415	434	32
							416	434	32
							389	403	27
							54	68	21
							417	434	32
							438	451	30
							417	434	32
							389	403	27
							205	227	46
							389	404	27
							205	230	46
							104	136	18
							84	95	72
							177	196	40
19		PVX_074190	hypothetical protein	323	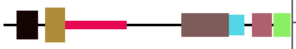		22	36	40
							255	269	27
							255	269	27
							255	269	27

							201	252	30
							255	270	27
							255	269	27
							255	270	27
							16	33	40
							281	300	32
							281	302	32
							49	64	46
							48	64	46
							281	300	32
							282	300	32
							255	269	27
							16	38	40
							283	300	32
							304	323	30
							283	300	32
							255	269	27
							255	270	27
							71	138	9
							49	69	46
							19	38	40
20		PVX_020170	hypothetical protein	333	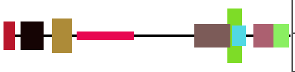		265	279	27
							265	279	27
							265	279	27
							1	13	39
							261	275	74
							222	262	30
							265	280	27
							265	279	27
							265	280	27
							21	40	40
							291	310	32
							291	312	32
							59	74	46
							58	75	46
							291	310	32
							292	310	32
							265	279	27
							26	46	40
							293	310	32
							314	330	30
							293	310	32
							265	279	27
							265	280	27
							85	151	9

								59	79	46
	21	Not clustered	PVX_183280	variable surface protein Vir12-related	443	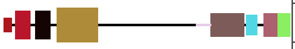		375	389	27
								376	387	27
								376	387	27
								18	40	39
								321	372	30
								375	390	27
								375	389	27
								375	390	27
								298	320	4
								49	68	40
								402	419	32
								402	422	32
								1	13	18
								402	419	32
								402	419	32
								376	387	27
								49	73	40
								403	419	32
								424	443	30
								403	419	32
								375	389	27
								375	390	27
								84	144	46
	22		PVX_004495	hypothetical protein, conserved	449	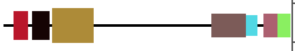		381	395	27
								382	393	27
								382	393	27
								16	38	39
								327	378	30
								381	396	27
								381	395	27
								381	396	27
								47	66	40
								408	426	32
								408	428	32
								408	426	32
								408	426	32
								382	393	27
								47	71	40
								409	426	32
								430	449	30
								409	426	32
								381	395	27
								381	396	27
								78	140	46

23	Not clustered	PVX_120845	variable surface protein Vir12-related	587	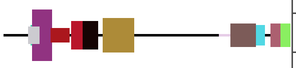		519	533	27
							520	531	27
							520	531	27
							88	97	72
							139	161	39
							465	516	30
							519	534	27
							56	102	11
							519	533	27
							519	534	27
							443	462	4
							57	67	28
							163	188	40
							546	563	32
							546	566	32
							98	133	18
							97	133	18
							61	96	72
							52	72	21
							546	563	32
							546	563	32
							520	531	27
							171	193	40
							57	70	21
							547	563	32
							568	587	30
							547	563	32
							519	533	27
							519	534	27
							203	265	46
							83	96	72
24	E	PVX_101620	variable surface protein Vir16-related	594	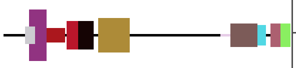		526	540	27
							527	538	27
							527	538	27
							133	155	39
							472	523	30
							526	541	27
							48	95	11
							526	540	27
							526	541	27
							450	469	4
							157	182	40
							553	570	32
							553	573	32
							90	127	18

							54	89	72
							46	65	21
							553	570	32
							553	570	32
							527	538	27
							165	187	40
							49	63	21
							554	570	32
							575	594	30
							554	570	32
							526	540	27
							526	541	27
							197	259	46
							74	89	72
25	Not clustered	PVX_115985	variable surface protein Vir, putative	574	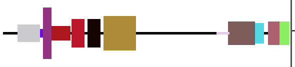		506	520	27
							507	518	27
							507	518	27
							139	161	39
							452	503	30
							506	521	27
							53	105	11
							506	520	27
							506	521	27
							429	451	4
							172	187	40
							533	551	32
							533	553	32
							97	134	18
							30	72	21
							533	551	32
							533	551	32
							507	518	27
							172	194	40
							56	69	21
							534	551	32
							555	574	30
							534	551	32
							506	520	27
							506	521	27
							203	265	46
							81	95	72
							84	95	72
26		PVX_065690	hypothetical protein	394	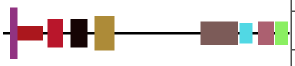		100	116	40
							326	340	27
							326	340	27

							326	340	27
							62	82	39
							272	323	30
							326	341	27
							326	340	27
							326	341	27
							352	371	32
							352	371	32
							21	55	18
							352	371	32
							353	371	32
							326	340	27
							94	116	40
							98	115	40
							354	371	32
							375	391	30
							354	371	32
							326	340	27
							127	153	46
							326	341	27
							10	18	72
							121	137	40
							336	350	27
							336	350	27
							336	350	27
							88	102	39
							282	333	30
							336	351	27
							336	350	27
							336	351	27
							362	380	32
							362	382	32
27		PVX_034690	hypothetical protein	404	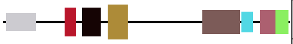		5	45	21
							362	380	32
							363	380	32
							336	350	27
							113	137	40
							119	136	40
							364	380	32
							385	400	30
							364	380	32
							336	350	27
							148	174	46
							336	351	27
28		PVX_036690	hypothetical	98	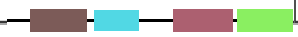		30	44	27

							31	42	27
							31	42	27
							8	27	30
							30	45	27
							30	44	27
							30	45	27
							57	74	32
							57	77	32
							57	74	32
							57	74	32
							31	42	27
							58	74	32
							79	98	30
							58	74	32
							30	44	27
							30	45	27
29		PVX_061690	hypothetical protein	273	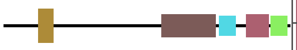		205	219	27
							205	219	27
							205	219	27
							151	202	30
							205	220	27
							205	219	27
							205	220	27
							231	249	32
							231	252	32
							231	249	32
							232	249	32
							205	219	27
							233	249	32
							254	270	30
							233	249	32
							205	219	27
							34	47	46
							205	220	27
30		PVX_027190	hypothetical protein	200	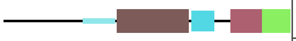		131	145	27
							131	145	27
							131	145	27
							79	128	30
							131	146	27
							131	145	27
							131	146	27
							158	178	32
							158	177	32
							158	177	32
							159	177	32

							131	145	27
							160	179	32
							180	200	30
							160	177	32
							131	145	27
							131	146	27
							55	77	6
31		PVX_049690	hypothetical protein	366	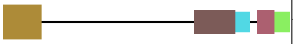		298	312	27
							299	310	27
							299	310	27
							244	295	30
							298	313	27
							298	312	27
							298	313	27
							325	343	32
							325	345	32
							325	343	32
							325	343	32
							299	310	27
							326	343	32
							347	366	30
							326	343	32
							298	312	27
							298	313	27
							1	49	46
32		PVX_037690	hypothetical protein	67	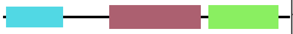		1	13	27
							1	13	27
							1	13	27
							1	14	27
							1	13	27
							1	14	27
							25	44	32
							25	46	32
							25	44	32
							26	44	32
							1	13	27
							27	44	32
							48	64	30
							27	44	32
							1	13	27
							1	14	27
33	Not clustered	PVX_094245	variable surface protein Vir12-like	584	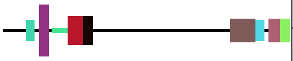		515	529	27
							515	529	27
							515	529	27
							134	162	39

							73	93	72
							463	512	30
							515	530	27
							515	529	27
							515	530	27
							542	562	32
							542	561	32
							542	561	32
							543	561	32
							515	529	27
							102	131	6
							49	64	28
							544	563	32
							564	584	30
							544	561	32
							515	529	27
							515	530	27
							165	183	40
34	E	PVX_015635	variable surface protein Vir12-like	529	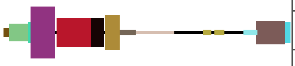		520	529	27
							520	529	27
							520	529	27
							46	68	28
							45	60	28
							245	314	4
							468	517	30
							520	529	27
							245	314	4
							520	529	27
							520	529	27
							11	43	23
							391	406	8
							370	381	8
							98	162	39
							140	150	39
							188	211	46
							12	29	23
							193	213	46
							52	94	72
							520	529	27
							215	241	5
							215	243	5
							520	529	27
							520	529	27
							256	312	4
							246	314	4

							1	9	9
							444	466	6
							196	214	46
							75	86	72
							163	183	40
35	E	PVX_106220	variable surface protein Vir12, putative	481	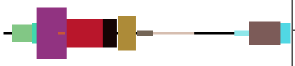		465	479	27
							465	479	27
							465	479	27
							51	75	28
							50	67	28
							250	319	4
							413	462	30
							465	474	27
							249	319	4
							465	479	27
							465	474	27
							16	48	23
							54	64	28
							103	167	39
							194	216	46
							17	31	23
							198	217	46
							56	104	72
							465	479	27
							226	244	5
							225	248	5
							465	479	27
							465	474	27
							261	317	4
							93	102	4
							251	319	4
							389	411	6
							201	219	46
							80	91	72
							168	188	40
36	E	PVX_017140	variable surface protein Vir12-like	568	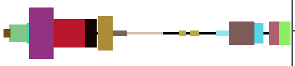		499	513	27
							499	513	27
							499	513	27
							47	61	28
							46	66	28
							244	313	4
							447	496	30
							499	514	27
							12	25	23
							243	313	4

							499	513	27
							1	8	4
							499	514	27
							13	44	23
							370	385	8
							348	360	8
							101	163	39
							526	546	32
							141	151	39
							526	545	32
							188	212	46
							1	8	4
							141	152	39
							194	213	46
							53	97	72
							526	545	32
							527	545	32
							499	513	27
							216	240	5
							216	242	5
							528	547	32
							548	568	30
							528	545	32
							499	513	27
							499	514	27
							255	311	4
							245	313	4
							1	12	9
							423	445	6
							76	87	72
							164	183	40
37	E	PVX_107235	variable surface protein Vir12-related	582	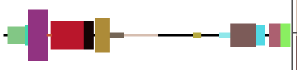		162	182	40
							514	527	27
							514	527	27
							514	527	27
							46	70	28
							45	59	28
							245	313	4
							461	510	30
							514	528	27
							245	313	4
							514	527	27
							514	528	27
							11	43	23
							387	399	8

						98	162	39
						540	560	32
						140	150	39
						540	559	32
						187	211	46
						12	29	23
						193	213	46
						51	89	72
						540	559	32
						541	559	32
						514	527	27
						215	241	5
						215	243	5
						542	561	32
						562	582	30
						542	559	32
						514	527	27
						514	528	27
						255	311	4
						88	97	4
						246	313	4
						437	459	6
						196	214	46
						75	86	72
						163	183	40
38	E	PVX_078195	variable surface protein Vir12-related	569	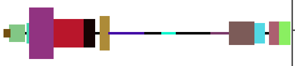	500	514	27
						500	514	27
						500	514	27
						47	71	28
						71	88	72
						412	445	4
						447	497	30
						500	515	27
						500	514	27
						500	515	27
						13	42	23
						94	161	39
						527	547	32
						139	149	39
						527	546	32
						191	209	46
						315	340	4
						52	97	72
						527	546	32
						528	546	32

							500	514	27
							529	548	32
							549	569	30
							529	546	32
							500	514	27
							210	251	4
							254	276	4
							500	515	27
							1	12	9
							75	88	72
							75	86	72
							162	182	40
39	E	PVX_013620	variable surface protein Vir12-like	537	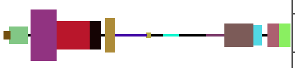		468	482	27
							468	482	27
							468	482	27
							380	413	4
							415	465	30
							468	483	27
							468	482	27
							468	483	27
							13	44	23
							267	276	8
							94	161	39
							495	515	32
							139	149	39
							495	514	32
							191	209	46
							300	325	4
							52	97	72
							495	514	32
							496	514	32
							468	482	27
							497	516	32
							517	537	30
							497	514	32
							468	482	27
							210	266	4
							468	483	27
							1	12	9
							75	86	72
							162	182	40
40	E	PVX_077695	variable surface protein Vir12-like	545	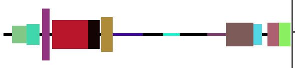		476	490	27
							476	490	27
							476	490	27
							46	67	28

							388	421	4
							423	473	30
							476	490	27
							476	490	27
							476	490	27
							19	42	23
							94	160	39
							503	523	32
							138	148	39
							503	522	32
							186	207	46
							303	320	4
							318	333	4
							503	522	32
							504	522	32
							476	490	27
							505	524	32
							525	545	30
							505	522	32
							476	490	27
							208	264	4
							476	490	27
							74	87	72
							74	85	72
							161	180	40
41	E	PVX_022685	variable surface protein Vir24-related	630	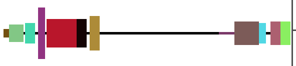		561	575	27
							561	575	27
							561	575	27
							48	69	28
							473	506	4
							510	558	30
							561	575	27
							561	575	27
							1	8	4
							561	575	27
							13	44	23
							95	162	39
							588	608	32
							140	150	39
							588	607	32
							191	209	46
							1	8	4
							588	607	32
							589	607	32
							561	575	27

							590	609	32
							610	630	30
							590	607	32
							561	575	27
							561	575	27
							76	89	72
							1	12	9
							76	87	72
							163	182	40
42	E	PVX_097525	variable surface protein Vir 12, putative	508	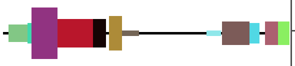		439	453	27
							439	453	27
							439	453	27
							45	69	28
							48	60	28
							389	436	30
							439	454	27
							15	25	23
							439	453	27
							439	454	27
							11	43	23
							94	161	39
							466	486	32
							139	149	39
							466	485	32
							188	209	46
							51	96	72
							466	485	32
							467	485	32
							439	453	27
							213	241	5
							213	241	5
							468	487	32
							488	508	30
							468	485	32
							439	453	27
							439	454	27
							363	385	6
							74	85	72
							162	182	40
43	L	PVX_102640	variable surface protein Vir12-related	441	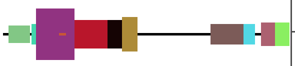		372	386	27
							372	386	27
							372	386	27
							45	70	28
							321	369	30
							372	387	27

								13	25	23
								372	386	27
								372	387	27
								11	41	23
								97	161	39
								399	419	32
								139	149	39
								399	418	32
								185	207	46
								51	110	72
								399	418	32
								400	418	32
								372	386	27
								401	420	32
								421	441	30
								401	418	32
								372	386	27
								372	387	27
								86	96	4
								74	84	72
								162	181	40
	44	E	PVX_001625	variable surface protein Vir12-related	460	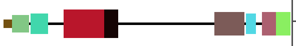		389	403	27
								389	403	27
								389	403	27
								45	70	28
								339	386	30
								389	404	27
								16	27	23
								389	403	27
								1	8	4
								389	404	27
								14	41	23
								97	162	39
								416	436	32
								140	150	39
								416	435	32
								1	8	4
								140	151	39
								416	435	32
								417	435	32
								389	403	27
								418	437	32
								438	458	30
								418	435	32
								389	403	27

							389	404	27
							1	12	9
							163	183	40
45	Not clustered	PVX_020680	variable surface protein Vir12-like	581	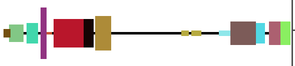		512	526	27
							512	526	27
							512	526	27
							48	67	28
							460	509	30
							512	527	27
							12	25	23
							512	526	27
							1	8	4
							512	527	27
							13	39	23
							383	398	8
							361	373	8
							102	163	39
							539	559	32
							141	151	39
							539	558	32
							188	212	46
							1	8	4
							194	213	46
							539	558	32
							540	558	32
							512	526	27
							541	560	32
							561	581	30
							541	558	32
							512	526	27
							512	527	27
							88	98	4
							1	12	9
							436	458	6
							197	215	46
							76	87	72
							164	183	40
46	L	PVX_041690	variable surface protein Vir12-related, truncated	379	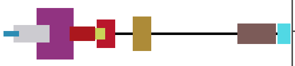		363	377	27
							363	377	27
							363	377	27
							124	146	39
							309	360	30
							363	378	27
							100	133	13
							30	82	11

							363	377	27
							363	378	27
							45	91	72
							15	60	21
							363	377	27
							363	377	27
							172	194	46
							363	378	27
							69	81	72
							1	20	7
							69	81	72
							89	120	18
							69	80	72
47	E	PVX_133260	variable surface protein Vir12, putative, truncated	268	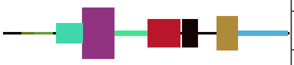		30	43	4
							136	165	39
							91	104	72
							76	104	72
							18	29	4
							220	266	5
							220	266	5
							75	90	72
							105	135	6
							50	74	28
							30	46	4
							200	219	46
							168	181	40
48	E	PVX_158260	variable surface protein Vir12, truncated, putative	287	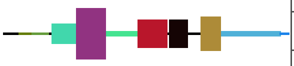		169	185	40
							30	41	4
							135	164	39
							90	103	72
							75	103	72
							17	28	4
							219	277	5
							219	277	5
							278	287	4
							74	89	72
							104	134	6
							49	73	28
							29	45	4
							199	218	46
							167	185	40
49	E	PVX_170270	variable surface protein Vir12, truncated, putative	332	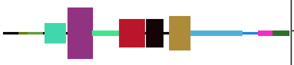		169	185	40
							30	41	4
							136	164	39
							75	103	72

							298	311	7
							19	28	4
							219	277	5
							219	277	5
							278	301	4
							144	154	39
							313	331	8
							193	215	46
							144	155	39
							104	134	6
							49	73	28
							29	45	4
							313	331	8
							76	89	72
							297	311	7
							297	311	7
							199	217	46
							167	180	40
50	Not clustered	PVX_157260	variable surface protein Vir12, truncated, putative	315	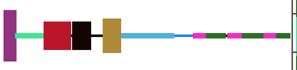		79	95	40
							45	74	39
							1	13	72
							286	299	7
							247	260	7
							129	186	5
							129	186	5
							188	211	4
							300	315	8
							223	244	8
							262	284	8
							14	44	6
							301	315	8
							223	243	8
							262	284	8
							246	260	7
							286	299	7
							208	221	7
							286	299	7
							246	260	7
							208	221	7
							109	128	46
							77	95	40
51	Not clustered	PVX_134260	variable surface protein Vir12, putative, truncated	104	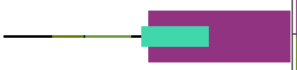		30	43	4
							76	104	72
							18	29	4
							53	101	72

						50	74	28
						30	46	4
52	Not clustered	PVX_104180	variable surface protein Vir12, putative	386	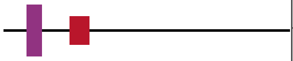	89	115	39
						34	52	72
						32	47	72
						31	48	72
53		PVX_001640	hypothetical protein	227	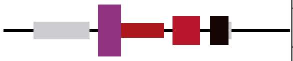	167	180	21
						134	155	39
						164	178	40
						93	126	18
						24	68	21
						53	64	21
						79	92	72
						75	93	72
						79	90	72
54	E	PVX_112630	variable surface protein Vir12, putative	650	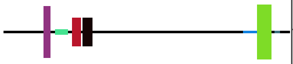	580	606	74
						543	575	4
						91	105	72
						578	606	74
						158	173	39
						615	624	4
						118	145	6
						181	199	40
55	E	PVX_110295	variable surface protein Vir22-like	553	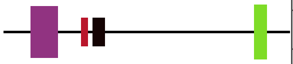	176	194	40
						484	507	74
						484	507	74
						495	508	74
						151	162	39
						54	103	72
						174	193	40
56	Not clustered	PVX_097545	variable surface protein Vir12-related	451	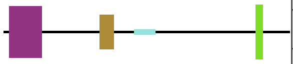	397	408	74
						33	51	72
						152	173	46
						11	60	72
						206	239	6
57	E	PVX_002485	variable surface protein Vir12-related	276	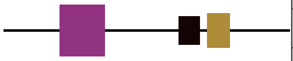	197	217	46
						55	97	72
						169	189	40
58	E	PVX_002490	variable surface protein Vir12-related	464	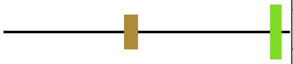	433	449	74
						433	449	74
						196	216	46
59	E	PVX_172270	variable surface protein Vir22, truncated, putative	378	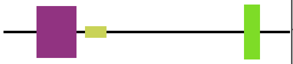	109	136	13
						319	337	74
						45	96	72
						320	328	74

							65	85	72
60	E	PVX_009595	variable surface protein Vir22, putative	392	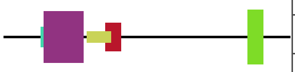		141	159	39
							51	73	28
							114	146	13
							334	354	74
							55	108	72
							335	343	74
							75	95	72
61	E	PVX_110300	variable surface protein Vir 12/22/24-like	359	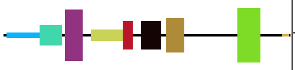		173	196	40
							293	310	74
							293	310	74
							4	45	7
							4	46	7
							350	357	4
							110	148	13
							149	159	39
							301	321	74
							149	161	39
							203	226	46
							304	313	74
							46	72	28
							78	98	72
62	E	PVX_007085	variable surface protein Vir24-related	321	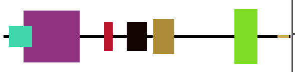		139	160	40
							259	276	74
							259	276	74
							308	319	4
							266	283	74
							113	121	39
							50	62	72
							266	283	74
							168	190	46
							24	84	72
							269	279	74
							8	32	28
63	E	PVX_088805	variable surface protein Vir22/24-like	358	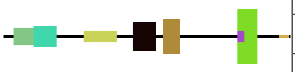		166	188	40
							293	313	74
							49	63	28
							293	313	74
							345	355	4
							13	28	23
							102	140	13
							14	40	23
							292	305	13
							199	217	46
							301	316	74

							199	220	46
							304	313	74
							162	184	40
							39	65	28
							202	219	46
64	E	PVX_081850	variable surface protein Vir22/23-related	608	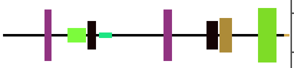		432	455	40
							180	197	40
							544	563	74
							205	230	5
							138	175	17
							544	563	74
							596	607	4
							461	482	46
							551	580	74
							463	485	46
							554	562	74
							89	101	72
							343	357	72
65	E	PVX_093720	variable surface protein Vir24-like	496	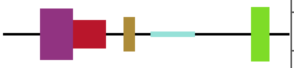		431	453	74
							66	82	28
							431	453	74
							439	456	74
							114	178	39
							155	165	39
							210	227	46
							439	459	74
							212	228	46
							66	120	72
							442	455	74
							257	330	6
							88	100	72
66	E	PVX_002480	variable surface protein Vir12/24-related	616	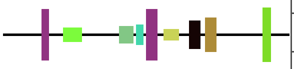		402	425	40
							130	167	17
							346	376	13
							250	280	23
							288	300	28
							558	573	74
							435	457	46
							561	569	74
							310	330	72
							86	98	72
							316	328	72
67	Not clustered	PVX_241290	variable surface protein Vir12-like	771	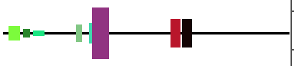		459	472	39
							84	108	5
							454	476	39

#	Cluster	ID	Description	Length	Structure	Start	End	Domain
						17	43	17
						197	212	23
						232	251	28
						269	281	72
						199	211	23
						241	282	72
						56	71	10
						265	280	72
						266	281	72
						485	506	40
68	Not clustered	PVX_086855	variable surface protein Vir24-like	721	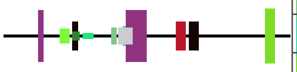	174	187	40
						468	488	40
						201	226	5
						434	456	39
						301	319	28
						143	165	17
						272	282	23
						300	316	28
						657	678	74
						307	358	72
						292	322	21
						174	189	10
						88	99	72
						331	346	72
						467	488	40
69	C	PVX_101565	variable surface protein Vir24-related	473	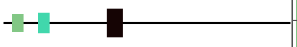	176	195	40
						57	74	28
						16	33	23
						18	27	23
						171	194	40
						176	195	40
70	E	PVX_086860	variable surface protein Vir24-related	447	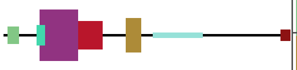	136	154	39
						78	98	72
						8	23	23
						56	64	28
						116	153	39
						142	152	39
						10	21	23
						191	213	46
						56	116	72
						52	64	28
						233	309	6
						77	91	72
						433	445	13
						75	91	72

71	E	PVX_083575	variable surface protein Vir24-related	510	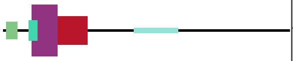	7	17	23
						98	148	39
						138	148	39
						10	23	23
						51	96	72
						47	59	28
						234	310	6
						72	86	72
72	E	PVX_097530	variable surface protein Vir22/12-related	524	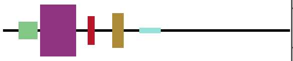	31	47	23
						33	61	23
						155	165	39
						155	166	39
						69	132	72
						251	287	6
						200	220	46
73	E	PVX_097555	variable surface protein Vir12/16-related	516	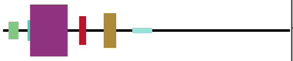	44	58	28
						11	26	23
						47	58	28
						138	148	39
						60	115	72
						50	115	72
						234	268	6
						183	202	46
74	E	PVX_124715	variable surface protein Vir5-related	528	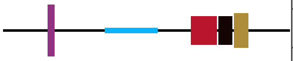	398	420	40
						188	283	7
						82	93	72
						348	393	39
						375	385	39
						429	444	46
						427	450	46
75		PVX_125726	hypothetical protein	364	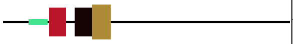	91	114	40
						59	79	39
						68	78	39
						113	135	46
						117	136	46
						34	55	6
76	Not clustered	PVX_088780	variable surface protein Vir24-related	446	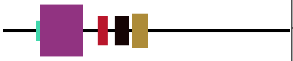	175	195	40
						149	161	39
						53	71	28
						151	161	39
						201	223	46
						59	123	72
77	Not clustered	PVX_112720	variable surface protein Vir12-related	505	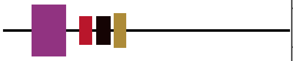	166	188	40
						141	154	39
						134	156	39

							51	64	28
							143	153	39
							196	212	46
							195	215	46
							51	109	72
							74	85	72
78	E	PVX_106725	variable surface protein Vir12, putative	469	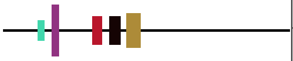		175	191	40
							146	161	39
							57	68	28
							202	225	33
							202	223	46
							80	91	72
79		PVX_124725	hypothetical protein	220	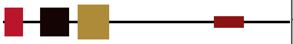		29	50	40
							2	15	39
							4	14	39
							4	15	39
							58	81	46
							29	46	40
							162	184	13
							29	47	40
80	Not clustered	PVX_088785	variable surfave protein Vir12, putative	314	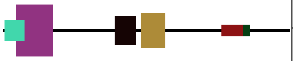		123	146	40
							35	52	72
							261	270	15
							152	170	46
							153	176	46
							15	54	72
							3	22	28
							240	261	13
81	E	PVX_003510	variable surface protein Vir24-related	453	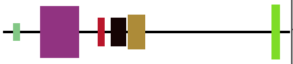		174	194	40
							424	437	74
							424	437	74
							150	160	39
							199	215	46
							16	27	23
							199	220	46
							61	120	72
							199	222	46
							174	186	10
							199	222	46
							172	191	40
							83	96	72
							84	100	72
							85	96	72
							176	194	40
82	E	PVX_104190	variable surface	426	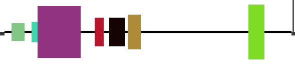		157	177	40

							364	381	74
							363	381	74
							42	61	28
							135	145	39
							12	28	23
							185	202	46
							51	111	72
							372	384	74
							186	202	46
							74	88	72
							158	177	40
83		PVX_090295	hypothetical protein	542	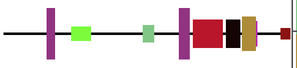		423	445	40
							130	164	17
							265	278	23
							359	412	39
							399	409	39
							452	479	33
							266	283	23
							452	475	46
							422	441	40
							524	541	13
							458	474	46
							83	96	72
							332	350	72
							335	344	72
84	E	PVX_097540	variable surface protein Vir24-related	668	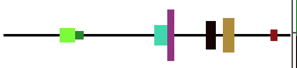		472	494	40
							354	381	28
							131	164	17
							512	533	46
							515	536	46
							169	184	10
							472	490	40
							622	637	13
							517	533	46
							384	396	72
							384	394	72
							472	491	40
85	Unavailable	PVX_096910	variable surface protein Vir23-related	506	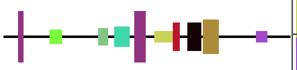		326	347	40
							81	103	17
							168	184	23
							266	300	13
							447	463	13
							301	311	39
							354	375	46
							170	182	23

					355	378	46
					196	222	28
					26	33	72
					233	248	72
					359	376	46
					231	248	72
					326	344	40
86	E	PVX_115485	variable surface protein Vir24-related	302	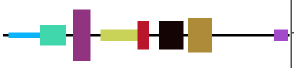	166	189	40
					7	39	7
					104	142	13
					286	300	13
					143	153	39
					143	154	39
					196	219	46
					40	66	28
					200	214	46
					74	92	72
87	E	PVX_107755	variable surface protein Vir12/24-related	657	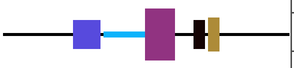	441	461	40
					233	326	7
					162	223	40
					165	220	40
					474	492	46
					472	495	46
					328	392	72
					439	459	40
					351	364	72
					477	494	46
					441	460	40
88	E	PVX_003505	variable surface protein Vir24-related	414	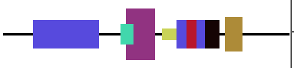	292	312	40
					203	217	72
					230	265	13
					44	137	40
					57	105	40
					252	308	40
					59	80	40
					267	278	39
					322	345	46
					179	218	72
					292	308	40
					171	187	28
					203	216	72
					327	342	46
					200	216	72
					292	308	40
89	E	PVX_119210	variant surface	540	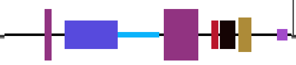	410	432	40

							180	227	7
							229	289	7
							115	211	40
							77	88	72
							515	530	13
							123	173	40
							390	400	39
							441	464	46
							300	363	72
							405	428	40
							322	335	72
							322	339	72
							324	335	72
90		PVX_094250	hypothetical protein	525	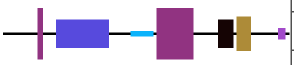		397	420	40
							234	274	7
							98	194	40
							63	73	72
							504	517	13
							430	452	46
							283	347	72
							395	416	40
							306	319	72
							306	319	72
							308	319	72
91		PVX_086865	hypothetical protein	576	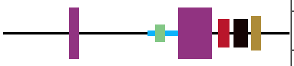		468	490	40
							433	453	39
							291	351	7
							308	324	23
							136	145	72
							443	453	39
							135	150	72
							499	517	46
							353	418	72
							465	486	40
							376	389	72
							377	393	72
							378	390	72
92		PVX_097542	hypothetical protein	560	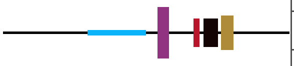		396	418	40
							167	278	7
							373	382	39
							428	450	46
							392	414	40
							305	317	72
							303	322	72
93		PVX_086895	hypothetical	553	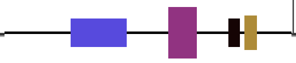		432	453	40

						129	234	40
						339	355	72
						462	484	46
						316	370	72
						339	353	72
						337	355	72
						340	351	72
94		PVX_001635	hypothetical protein, conserved	634	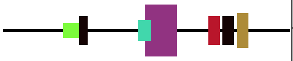	171	184	40
						487	509	40
						454	477	39
						310	334	28
						134	165	17
						463	472	39
						519	533	46
						518	540	46
						316	382	72
						171	184	10
						169	184	40
						486	505	40
						300	325	28
						340	355	72
						171	184	40
						487	506	40
95	E	PVX_003495	variable surface protein Vir22/24-related	635	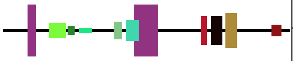	463	485	40
						169	194	5
						285	308	28
						104	139	17
						247	263	23
						439	449	39
						249	261	23
						439	450	39
						494	516	46
						292	341	72
						145	158	10
						463	474	10
						462	481	40
						275	300	28
						57	70	72
						315	329	72
						596	614	13
						314	331	72
96	Not clustered	PVX_083580	variable surface protein Vir24-related	575	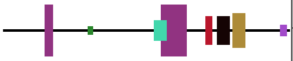	431	453	40
						344	360	72
						556	569	13
						407	417	39

							344	356	72
							462	484	46
							318	366	72
							172	181	10
							302	327	28
							86	100	72
							341	354	72
							87	99	72
							339	356	72
97		PVX_003490	hypothetical protein, conserved	612	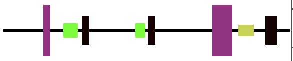		310	323	40
							560	582	40
							471	487	72
							130	157	17
							282	301	17
							502	535	13
							449	487	72
							170	183	10
							310	323	10
							170	181	40
							310	323	40
							470	484	72
							469	486	72
							86	98	72
							471	482	72
98	E	PVX_107745	variable surface protein Vir 12-like	351	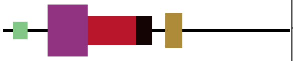		164	182	24
							140	154	39
							13	29	23
							100	164	39
							144	154	39
							199	217	46
							199	218	46
							55	103	72
							164	181	40
							78	89	72
99		PVX_107740	hypothetical protein	304	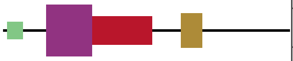		5	21	23
							92	157	39
							135	145	39
							190	205	46
							189	210	46
							47	94	72
							70	81	72
100		PVX_107735	hypothetical protein	362	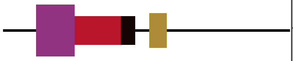		88	151	39
							131	141	39
							186	202	46
							185	206	46

							42	90	72
							150	166	40
101	E	PVX_022185	variable surface protein Vir12-related	352	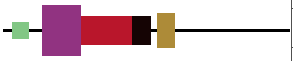		12	23	23
							11	30	23
							93	157	39
							136	142	39
							190	211	46
							48	95	72
							160	172	40
							71	82	72
							161	180	40
102	E	PVX_090290	variable surface protein Vir12, putative	288	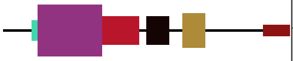		30	52	28
							119	135	39
							91	136	39
							125	131	39
							180	203	46
							35	99	72
							144	167	40
							58	72	72
							262	288	13
							59	74	72
103		PVX_004537	hypothetical protein	466	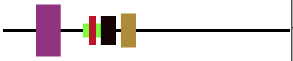		132	157	17
							141	150	39
							77	91	72
							193	210	46
							194	216	46
							55	93	72
							159	178	40
							164	183	40
104		PVX_005055	hypothetical protein	534	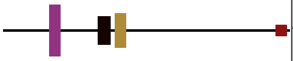		89	104	72
							90	104	72
							89	105	72
							209	225	46
							209	227	46
							90	103	72
							509	526	13
							210	226	46
							88	105	72
							178	200	40
105		PVX_090310	hypothetical protein, conserved	449	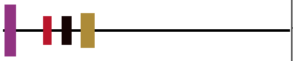		63	76	39
							4	20	72
							4	18	72
							122	140	46
							122	142	46
							93	106	40

						4	12	72
						3	17	72
						125	142	46
						4	18	72
106	E	PVX_001645	variable surface protein Vir12-related	529	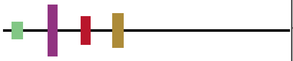	144	160	39
						83	99	72
						202	220	46
						17	36	23
						202	221	46
						205	221	46
107	Unavailable	PVX_005045	variable surface protein Vir12-related	445	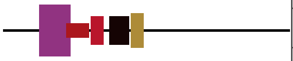	136	154	39
						78	92	72
						199	216	46
						56	104	72
						165	194	40
						204	217	46
						78	92	72
						200	216	46
						78	93	72
						99	132	18
						79	90	72
108	E	PVX_090305	variable surface protein Vir12-related	462	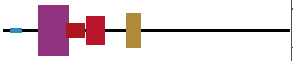	138	162	39
						135	157	39
						201	216	46
						200	216	46
						56	105	72
						78	91	72
						12	28	7
						201	221	46
						78	91	72
						103	131	18
						80	91	72
109	E	PVX_093710	variable surface protein Vir24-related	488	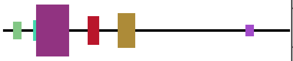	145	162	39
						52	73	28
						18	31	23
						414	425	13
						149	159	39
						82	96	72
						196	220	46
						202	223	46
						58	111	72
						82	96	72
						204	222	46
						82	96	72
						83	94	72

110	Not clustered	PVX_124712	variable surface protein Vir24-related	400	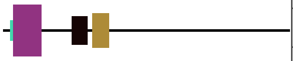		10	25	28
							37	51	72
							125	142	46
							125	146	46
							14	53	72
							37	51	72
							33	52	72
							38	49	72
							96	116	40
111	E	PVX_018660	variable surface protein Vir24-like	493	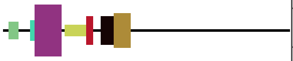		49	73	28
							79	93	72
							10	27	23
							107	143	13
							145	155	39
							192	215	46
							199	217	46
							55	99	72
							79	92	72
							79	90	72
							169	189	40
112	E	PVX_094240	variable surface protein Vir12/24-related	443	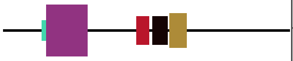		206	225	39
							62	89	28
							259	277	46
							259	282	46
							68	130	72
							92	107	72
							263	280	46
							232	254	40
113	E	PVX_001615	variable surface protein Vir12/24-related	366	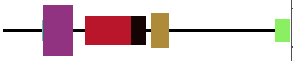		51	72	28
							106	162	39
							140	150	39
							189	208	46
							192	212	46
							53	89	72
							348	366	30
							195	212	46
							164	182	40
114	Not clustered	PVX_124720	variable surface protein Vir12-related	545	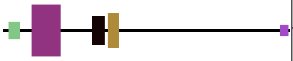		57	71	28
							80	96	72
							527	540	13
							12	31	23
							199	219	46
							57	107	72
							78	93	72
							202	219	46

							170	191	40
115	E	PVX_096920	variable surface protein Vir12-related	491	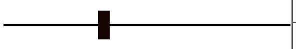		165	181	40
							163	179	40
1	Not clustered	PVX_004535	variable surface protein Vir, putative	330	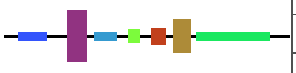		170	186	24
							73	95	72
							222	306	11
							24	48	10
							145	156	17
							105	130	11
							195	216	46
							195	216	46
							17	49	10
2		PVX_124700	hypothetical protein	255	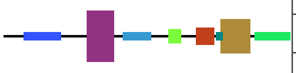		171	187	24
							74	97	72
							224	254	11
							25	49	10
							147	157	17
							189	194	9
							106	131	11
							193	219	46
							193	219	46
							189	194	9
							18	50	10
3	C	PVX_024690	variable surface protein Vir12-related	343	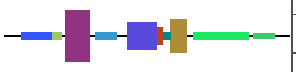		173	190	24
							75	102	72
							228	293	11
							29	57	10
							149	166	17
							148	182	40
							192	199	9
							83	95	72
							110	134	11
							200	219	46
							200	219	46
							300	323	7
							192	199	9
							59	70	10
							21	56	10
4	C	PVX_101600	variable surface protein Vir16/32-related	372	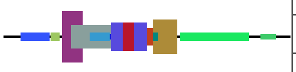		176	192	24
							78	102	72
							88	138	31
							229	317	11
							31	59	10
							151	168	17
							139	150	8

						141	185	40
						194	200	9
						155	169	39
						113	136	11
						194	224	46
						194	224	46
						333	352	7
						194	200	9
						62	72	10
						23	57	10
5	C	PVX_034190	variable surface protein Vir14-related	403	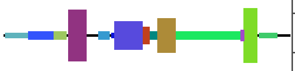	337	353	74
						189	205	24
						91	116	72
						242	332	11
						337	353	74
						3	33	8
						42	71	10
						164	181	17
						152	163	8
						333	347	13
						157	195	40
						206	214	9
						343	356	74
						133	149	11
						216	241	46
						340	356	74
						216	241	46
						360	383	7
						206	214	9
						72	87	10
						35	70	10
6	C	PVX_037190	variable surface protein Vir14/24-related	404	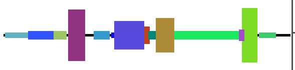	189	205	24
						91	114	72
						242	332	11
						337	353	74
						3	33	8
						42	71	10
						164	181	17
						152	163	8
						333	347	13
						157	196	40
						206	214	9
						342	356	74
						129	149	11
						216	239	46

								340	354	74
								216	239	46
								360	383	7
								206	214	9
								72	87	10
								35	70	10
	7	C	PVX_108770	variable surface protein Vir 14, putative	409	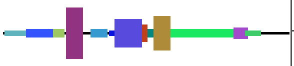		189	205	24
								90	114	72
								241	330	11
								241	331	11
								3	32	8
								40	70	10
								164	181	17
								152	163	8
								331	348	13
								161	198	40
								207	213	9
								126	149	11
								215	238	46
								215	238	46
								349	368	7
								346	368	7
								207	213	9
								72	86	10
								34	67	10
								96	110	72
	8	C	PVX_008085	variable surface protein Vir 14-related	418	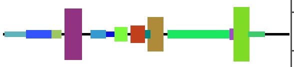		188	205	24
								90	114	72
								241	331	11
								3	32	8
								40	70	10
								164	181	17
								152	163	8
								332	346	13
								207	214	9
								129	149	11
								213	233	46
								337	358	74
								213	233	46
								359	382	7
								207	214	9
								73	84	10
								34	67	10
	9	C	PVX_103660	variable surface protein Vir16/32-	360	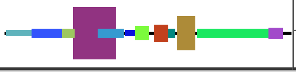		188	205	24
								91	116	72

				related					
							242	331	11
							242	332	11
							3	33	8
							41	71	10
							164	181	17
							152	163	8
							332	349	13
							206	214	9
							88	140	72
							118	149	11
							217	239	46
							217	239	46
							206	214	9
							72	87	10
							35	70	10
							95	111	72
	10	C	PVX_052690	variable surface protein Vir4-related, truncated	279	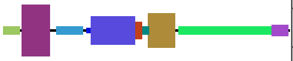	117	134	24
							19	45	72
							171	261	11
							93	110	17
							81	92	8
							262	277	13
							86	127	40
							136	141	9
							136	142	9
							52	78	11
							142	167	46
							142	167	46
							136	141	9
							136	142	9
							1	16	10
	11	Not clustered	PVX_019670	variable surface protein Vir12-related	172	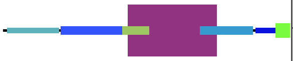	91	116	72
							3	33	8
							41	71	10
							164	172	17
							152	163	8
							75	128	72
							118	149	11
							72	87	10
							35	70	10
							95	111	72
	12	Not clustered	PVX_045690	variable surface protein Vir12-related	140	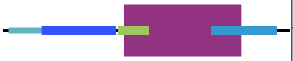	75	100	72
							3	18	8
							3	25	8
							25	55	10

							59	116	72
							102	133	11
							56	71	10
							19	54	10
13		PVX_090320	hypothetical protein	212	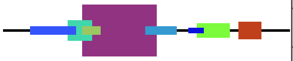		174	190	24
							76	101	72
							144	167	17
							28	56	10
							149	165	17
							137	148	8
							59	113	72
							105	128	11
							48	65	28
							59	72	10
							21	54	10
14		PVX_005065	hypothetical protein	198	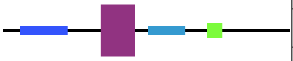		68	91	72
							19	43	10
							141	151	17
							100	125	11
							12	44	10
15	C	PVX_101615	variable surface protein Vir14-related	361	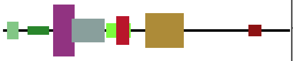		64	90	72
							130	159	17
							79	127	31
							6	19	23
							31	56	10
							72	84	72
							143	158	39
							180	227	46
							180	227	46
							31	55	10
							76	85	72
							309	324	13
16	C	PVX_124710	variable surface protein Vir4-related	313	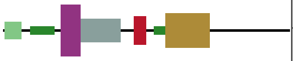		63	84	72
							73	128	31
							3	19	23
							30	55	10
							71	83	72
							143	156	39
							178	225	46
							165	177	10
							178	225	46
							30	54	10
17	C	PVX_120330	variable surface protein Vir14-related	418	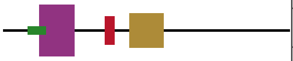		71	98	72
							37	62	10
							79	95	72

							150	161	39
							54	104	72
							185	232	46
							185	232	46
							37	62	10
18	C	PVX_004510	variable surface protein Vir4/14-related	353	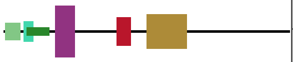		64	88	72
							3	20	23
							29	55	10
							141	156	39
							199	212	46
							178	225	46
							178	225	46
							25	37	28
							29	54	10
							75	84	72
19	C	PVX_115475	variable surface protein Vir14, putative	229	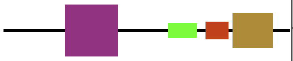		162	179	24
							70	91	72
							132	154	17
							50	87	72
							184	215	46
							184	215	46
20	C	PVX_113230	variable surface protein Vir14-related	440	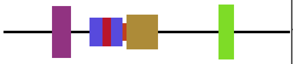		173	189	24
							75	102	72
							142	165	17
							134	182	40
							83	95	72
							330	354	74
							152	163	39
							190	236	46
							333	346	74
							190	236	46
21		PVX_094260	hypothetical protein	198	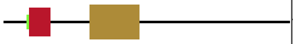		16	31	17
							18	32	39
							60	94	46
							60	94	46
22	C	PVX_095995	variable surface protein Vir16/32-related	315	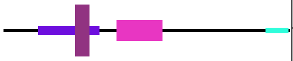		39	105	11
							289	313	8
							80	93	72
							125	174	25
23	C	PVX_004525	variable surface protein Vir16/32-related	353	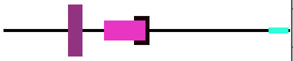		328	351	8
							162	179	40
							80	97	72
							125	161	25
							125	173	25
24	C	PVX_104185	variable surface	288	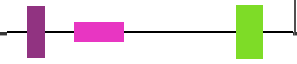		231	257	74

							21	38	72
							69	118	25
							21	38	72
25	C	PVX_127260	variable surface protein Vir16, putative, truncated	210	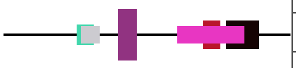		146	158	39
							54	66	28
							163	186	40
							84	96	72
							57	70	21
							85	97	72
							128	176	25
26	C	PVX_043690	variable surface protein Vir32, truncated, putative	85	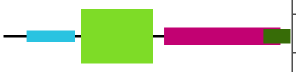		10	21	14
							30	41	74
							48	82	22
							48	75	22
							23	44	74
							77	85	17
							7	21	14
27	Not clustered	PVX_044190	variable surface protein Vir32, putative	61	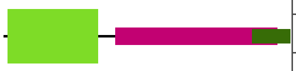		6	17	74
							24	58	22
							24	51	22
							1	20	74
							53	61	17
28	C	PVX_009090	variable surface protein Vir14-like	337	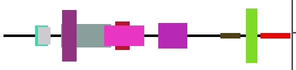		132	147	39
							285	297	74
							68	125	31
							256	278	6
							285	297	74
							38	52	28
							182	214	33
							304	337	6
							70	85	72
							286	297	74
							286	297	74
							41	55	21
							303	337	6
							70	81	72
							118	164	25
							70	85	72
29	C	PVX_072690	variable surface protein Vir4/14-related	324	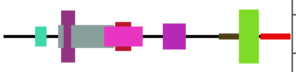		127	143	39
							274	284	74
							65	80	72
							62	124	31
							243	265	6
							274	284	74
							273	288	74

							37	47	28
							181	205	33
							291	324	6
							273	287	74
							266	284	74
							290	324	6
							69	76	72
							114	157	25
30	C	PVX_026190	variable surface protein Vir14, putative	353	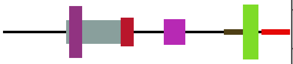		145	160	39
							301	313	74
							85	97	72
							78	144	31
							272	294	6
							301	313	74
							198	223	33
							320	353	6
							82	97	72
							296	314	74
							319	353	6
							82	94	72
							82	97	72
31	C	PVX_076695	variable surface protein Vir14/32-related	325	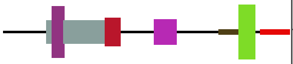		116	132	39
							273	285	74
							57	69	72
							50	115	31
							244	266	6
							273	285	74
							171	196	33
							292	325	6
							56	65	72
							268	285	74
							291	325	6
							56	67	72
							57	66	72
							57	65	72
32	C	PVX_028190	variable surface protein Vir4, putative	284	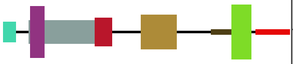		91	107	39
							234	244	74
							1	12	28
							26	90	31
							206	225	6
							234	244	74
							145	171	33
							251	284	6
							28	39	72
							137	171	46

						226	245	74
						137	171	46
						250	284	6
						28	40	72
33	C	PVX_103665	variable surface protein Vir4/32-like	290	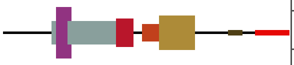	115	131	39
						142	164	24
						56	69	72
						50	114	31
						228	241	6
						142	157	24
						168	193	33
						257	290	6
						55	65	72
						159	193	46
						159	193	46
						256	290	6
						55	64	72
34	C	PVX_088800	variable surface protein Vir4-related	358	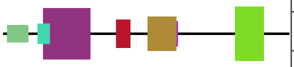	290	308	74
						290	308	74
						6	31	23
						190	217	33
						75	87	72
						298	325	74
						143	158	39
						51	109	72
						181	216	46
						298	306	74
						181	216	46
						44	58	28
						76	84	72
						75	87	72
						76	82	72
						75	90	72
						76	88	72
35	Unavailable	PVX_179275	variable surface protein Vir C1-29, pseudogene, putative	240	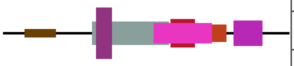	141	159	39
						168	187	24
						75	138	31
						18	44	12
						194	217	33
						79	91	72
						147	160	39
						79	90	72
						127	175	25
36	C	PVX_102125	variable surface protein Vir32,	153	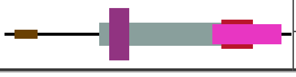	116	132	39
						51	115	31

			truncated, putative				
					[6](#)	[17](#)	[12](#)
					[56](#)	[66](#)	[72](#)
					[56](#)	[65](#)	[72](#)
					[111](#)	[147](#)	[25](#)
37	C	PVX_003485	variable surface protein Vir4, putative	329	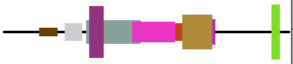		
					[190](#)	[212](#)	[24](#)
					[96](#)	[157](#)	[31](#)
					[42](#)	[61](#)	[12](#)
					[215](#)	[243](#)	[33](#)
					[100](#)	[115](#)	[72](#)
					[207](#)	[240](#)	[46](#)
					[308](#)	[317](#)	[74](#)
					[207](#)	[240](#)	[46](#)
					[72](#)	[89](#)	[21](#)
					[100](#)	[111](#)	[72](#)
					[159](#)	[182](#)	[25](#)
					[149](#)	[197](#)	[25](#)
38	C	PVX_096000	variable surface protein Vir4, putative	353	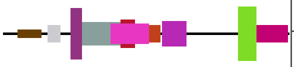		
					[146](#)	[162](#)	[39](#)
					[172](#)	[194](#)	[24](#)
					[84](#)	[144](#)	[31](#)
					[26](#)	[47](#)	[12](#)
					[201](#)	[212](#)	[33](#)
					[196](#)	[225](#)	[33](#)
					[84](#)	[97](#)	[72](#)
					[313](#)	[350](#)	[22](#)
					[290](#)	[311](#)	[74](#)
					[56](#)	[69](#)	[21](#)
					[84](#)	[95](#)	[72](#)
					[133](#)	[179](#)	[25](#)
					[18](#)	[44](#)	[12](#)
					[84](#)	[97](#)	[72](#)
39	C	PVX_005580	variable surface protein Vir4, putative	297	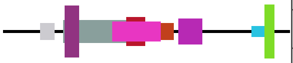		
					[128](#)	[146](#)	[39](#)
					[155](#)	[176](#)	[24](#)
					[63](#)	[127](#)	[31](#)
					[155](#)	[169](#)	[24](#)
					[182](#)	[206](#)	[33](#)
					[257](#)	[269](#)	[14](#)
					[64](#)	[77](#)	[72](#)
					[134](#)	[146](#)	[39](#)
					[271](#)	[280](#)	[74](#)
					[40](#)	[53](#)	[21](#)
					[258](#)	[269](#)	[14](#)
					[66](#)	[78](#)	[72](#)
					[114](#)	[163](#)	[25](#)
40	C	PVX_105700	variable surface protein Vir32/4,	340	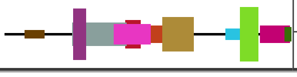		
					[144](#)	[161](#)	[39](#)
					[171](#)	[193](#)	[24](#)

#	Strand	ID	Description	Length	Architecture	Start	End	Domain
			putative			81	143	31
						24	47	12
						197	224	33
						267	278	14
						84	94	72
						287	298	74
						303	337	22
						188	223	46
						306	329	22
						280	300	74
						188	223	46
						332	340	17
						262	278	14
						84	94	72
						82	96	72
						130	173	25
41	C	PVX_051690	variable surface protein Vir32, putative	334	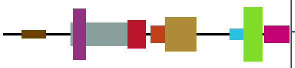	146	163	39
						173	194	24
						80	145	31
						23	49	12
						205	215	33
						200	225	33
						266	279	14
						84	95	72
						288	299	74
						304	332	22
						152	166	39
						190	224	46
						307	333	22
						281	302	74
						190	224	46
						264	280	14
						84	96	72
						82	93	72
42	C	PVX_061190	variable surface protein Vir32, putative	342	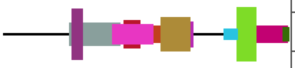	145	163	39
						172	193	24
						80	139	31
						202	214	33
						197	226	33
						268	278	14
						83	95	72
						303	339	22
						151	163	39
						189	223	46
						305	332	22

							280	301	74
							189	223	46
							334	342	17
							264	279	14
							83	94	72
							131	178	25
43	C	PVX_021680	variable surface protein Vir4, putative	355	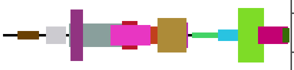		148	166	39
							175	196	24
							56	71	21
							83	147	31
							236	265	6
							236	268	6
							202	228	33
							299	322	74
							316	352	22
							192	226	46
							318	345	22
							292	313	74
							192	226	46
							347	355	17
							54	77	21
							267	291	14
							85	98	72
							134	182	25
							18	43	12
44	C	PVX_006580	variable surface protein Vir14, putative	351	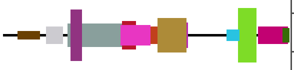		146	162	39
							173	194	24
							54	73	21
							80	145	31
							173	187	24
							200	226	33
							277	287	14
							296	307	74
							313	348	22
							191	224	46
							314	341	22
							289	310	74
							191	224	46
							343	351	17
							57	73	21
							274	288	14
							84	95	72
							144	180	25
							19	45	12
45	C	PVX_029190	variable surface	335	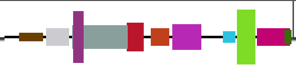		144	162	39

#	Class	ID	Description	Length	Domains	Start	End	Domain
						171	192	24
						50	74	21
						79	143	31
						171	184	24
						196	228	33
						279	290	74
						296	332	22
						298	324	22
						272	293	74
						327	335	17
						55	69	21
						256	268	14
						82	91	72
						17	44	12
46	C	PVX_175270	variable surface protein Vir14, truncated, putative	332	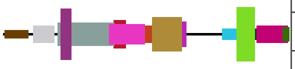	129	142	39
						156	178	24
						35	59	21
						64	128	31
						282	302	15
						156	170	24
						182	212	33
						255	269	14
						67	79	72
						294	329	22
						173	207	46
						297	323	22
						271	291	74
						173	207	46
						324	332	17
						256	270	14
						67	78	72
						123	163	25
						2	29	12
47	C	PVX_184285	variable surface protein Vir4, putative	348	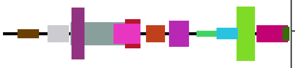	148	166	39
						175	196	24
						83	147	31
						236	259	6
						236	260	6
						175	189	24
						204	215	33
						202	225	33
						84	97	72
						309	345	22
						311	338	22
						285	306	74

								340	348	17
								54	79	21
								260	284	14
								85	99	72
								134	167	25
								18	43	12
	48	C	PVX_050190	variable surface protein Vir4, putative	340	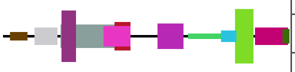		133	151	39
								68	132	31
								220	258	6
								189	200	33
								184	213	33
								71	86	72
								300	337	22
								303	330	22
								277	297	74
								332	340	17
								39	64	21
								259	276	14
								70	84	72
								119	151	25
								9	28	12
	49	C	PVX_016140	variable surface protein Vir4, putative	324	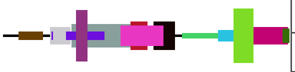		145	163	39
								172	193	24
								55	76	21
								79	144	31
								56	114	11
								204	242	6
								246	259	14
								83	95	72
								284	321	22
								287	314	22
								261	282	74
								316	324	17
								171	193	40
								57	70	21
								243	260	14
								83	94	72
								133	180	25
								19	45	12
	50	C	PVX_004520	variable surface protein Vir4, putative	363	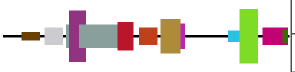		147	165	39
								174	196	24
								85	104	72
								81	144	31
								25	47	12
								200	230	33

						85	96	72
						330	360	22
						201	224	46
						333	349	22
						301	322	74
						355	361	17
						53	76	21
						286	299	14
						85	96	72
51	Unavailable	PVX_159260	variable surface protein Vir14, pseudogene, putative	541	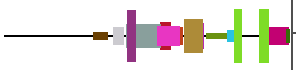	297	315	39
						208	227	21
						232	296	31
						392	420	6
						390	420	6
						324	338	24
						382	420	6
						349	378	33
						424	436	14
						235	247	72
						501	538	22
						341	375	46
						504	531	22
						438	447	74
						484	499	74
						341	375	46
						391	420	6
						533	541	17
						390	420	6
						235	246	72
						292	331	25
						169	196	12
52	C	PVX_030190	variable surface protein Vir14, truncated, putative	325	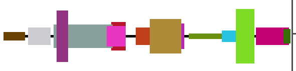	123	138	39
						150	172	24
						29	52	21
						57	122	31
						226	246	6
						225	246	6
						150	164	24
						210	246	6
						180	191	33
						175	204	33
						248	262	14
						287	322	22
						167	200	46
						290	316	22

							Start	End	Domain
							264	284	74
							167	200	46
							225	246	6
							317	325	17
							32	45	21
							225	246	6
							61	72	72
							118	138	25
							1	23	12
53	C	PVX_010610	variable surface protein Vir14, putative	315	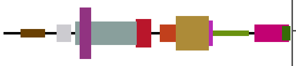		146	162	39
							173	194	24
							59	73	21
							80	145	31
							241	269	6
							239	269	6
							173	187	24
							231	269	6
							203	214	33
							199	229	33
							84	96	72
							277	312	22
							190	225	46
							280	306	22
							190	225	46
							240	269	6
							307	315	17
							239	269	6
							84	95	72
							20	45	12
54	C	PVX_169270	variable surface protein Vir14, putative	346	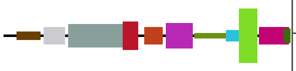		144	162	39
							171	192	24
							50	73	21
							78	143	31
							239	267	6
							239	267	6
							171	186	24
							231	267	6
							201	213	33
							196	227	33
							269	283	14
							308	343	22
							311	337	22
							285	305	74
							239	267	6
							338	346	17

							239	267	6
							17	44	12
55	C	PVX_149260	variable surface prtoein Vir14, truncated, putative	270	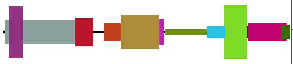		68	84	39
							95	116	24
							2	67	31
							163	191	6
							161	191	6
							220	241	15
							95	109	24
							153	191	6
							125	136	33
							121	151	33
							193	207	14
							6	18	72
							232	267	22
							112	147	46
							235	261	22
							209	229	74
							112	147	46
							162	191	6
							262	269	17
							194	208	14
							161	191	6
							6	17	72
56	Unavailable	PVX_052190	variable surface protein Vir4, pseudogene, putative	388	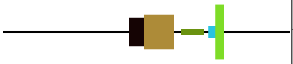		174	195	24
							250	270	6
							241	270	6
							174	188	24
							241	270	6
							172	189	40
							204	215	33
							200	225	33
							279	286	14
							191	229	46
							288	297	74
							191	229	46
							241	270	6
							241	270	6
57	Unavailable	PVX_066690	variable surface protein Vir16, pseudogene, putative	370	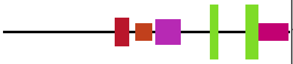		145	162	39
							172	191	24
							197	229	33
							331	367	22
							268	277	74
							314	329	74
58	C	PVX_027690	variable surface	315	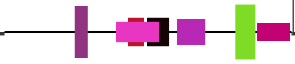		137	152	39

							157	180	40
							189	220	33
							78	90	72
							278	312	22
							255	275	74
							123	169	25
59	C	PVX_101560	variable surface protein Vir4-related	302			119	137	39
							146	163	24
							146	161	24
							179	197	33
							50	67	72
							100	153	25
60	Unavailable	PVX_046190	variable surface protein Vir32, putative; Truncated due to end of contig.	353			142	161	24
							168	180	33
							166	191	33
							240	247	14
							249	258	74
61	C	PVX_096945	variable surface protein Vir4-related	314			25	38	72
							21	82	31
							133	159	33
							20	32	72
							21	29	21
							24	32	72
62	Not clustered	PVX_086875	variable surface protein Vir27-related	292			265	285	9
							265	285	9
							134	161	12
							266	285	9
63		PVX_005575	hypothetical protein	138					
1	Not clustered	PVX_056190	variable surface protein Vir6-related	407			123	165	40
							123	151	40
							10	110	21
							370	400	29
2	Unavailable	PVX_014125	variable surface protein Vir6-like, pseudogene	523			182	224	40
							183	199	40
							398	428	29
3		PVX_060190	hypothetical protein	84			1	16	29
4	Not clustered	PVX_070190	variable surface protein Vir28, truncated, putative	182			83	114	29
5	Not clustered	PVX_106210	variable surface protein Vir6, putative	434			79	89	72
							10	109	21
							356	386	29
6	G	PVX_050690	variable surface	405			46	59	21

							350	376	74
							46	59	21
							20	43	7
							46	59	21
							94	134	40
							94	123	40
							21	45	7
							46	59	21
							16	80	21
							378	401	21
							318	349	29
7	G	PVX_059190	variable surface protein Vir6, putative	438	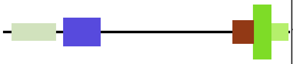		46	59	21
							383	409	74
							46	59	21
							20	42	7
							46	59	21
							96	148	40
							94	123	40
							21	45	7
							46	59	21
							15	80	21
							411	434	21
							351	382	29
8	Not clustered	PVX_079195	variable surface protein Vir6-like	427	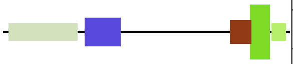		78	91	21
							370	396	74
							78	92	21
							53	64	7
							78	92	21
							123	173	40
							369	388	74
							123	152	40
							52	64	7
							78	92	21
							10	110	21
							401	420	21
							338	369	29
9	G	PVX_007080	variable surface protein Vir 28, putative	451	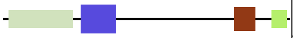		75	87	21
							75	87	21
							49	67	7
							75	87	21
							122	177	40
							122	149	40
							49	66	7
							75	87	21
							10	109	21

							422	445	21
							365	395	29
10	Not clustered	PVX_028690	variable surface protein Vir6, putative	464	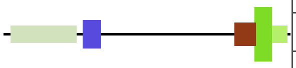		86	95	21
							81	95	21
							407	433	74
							86	95	21
							86	95	21
							128	156	40
							86	95	21
							86	94	21
							12	116	21
							435	458	21
							375	406	29
11	G	PVX_103670	variable surface protein Vir6-like	459	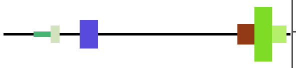		79	89	21
							76	87	21
							402	428	74
							79	89	21
							50	71	7
							79	89	21
							123	150	40
							50	74	7
							79	89	21
							78	87	21
							430	452	21
							375	401	29
12	G	PVX_007585	variable surface protein Vir28, putative	447	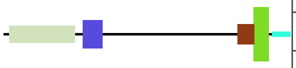		79	91	21
							420	446	8
							419	446	8
							76	87	21
							391	413	74
							79	91	21
							50	72	7
							79	91	21
							390	405	74
							125	152	40
							50	74	7
							79	91	21
							77	87	21
							11	111	21
							365	389	29
13	Not clustered	PVX_063690	variable surface protein Vir6-like	456	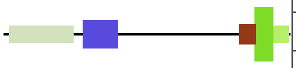		401	427	74
							126	182	40
							11	111	21
							429	452	21
							375	400	29

14	Not clustered	PVX_121345	variable surface protein Vir6, putative	449	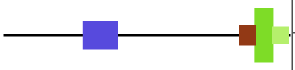		394	420	74
							125	179	40
							422	445	21
							370	393	29
15	G	PVX_026690	variable surface protein Vir6, putative	460	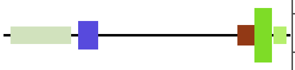		403	429	74
							121	151	40
							121	147	40
							75	84	21
							12	108	21
							434	452	21
							376	401	29
16	G	PVX_071190	variable surface protein Vir28-related	437	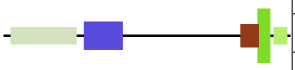		388	406	74
							124	180	40
							124	152	40
							78	84	6
							11	110	21
							411	431	21
							362	387	29
17		PVX_108255	hypothetical protein	442	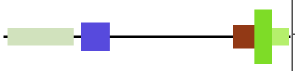		387	413	74
							46	66	7
							120	162	40
							120	148	40
							46	69	7
							7	107	21
							415	438	21
							355	386	29
18	Not clustered	PVX_029690	variable surface protein Vir6, putative	399	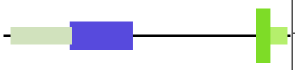		100	179	40
							352	370	74
							93	134	40
							93	122	40
							10	94	21
							372	395	21
19	G	PVX_035190	variable surface protein Vir6, truncated, putative	410	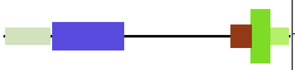		71	172	40
							34	44	21
							355	381	74
							354	368	74
							77	105	40
							4	66	21
							383	406	21
							324	354	29
20	Not clustered	PVX_073190	variable surface protein Vir6-like	492	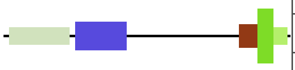		124	210	40
							78	85	21
							436	462	74
							123	150	40
							10	111	21

							464	486	21
							404	435	29
21	Not clustered	PVX_104695	variable surface protein Vir6-like	435	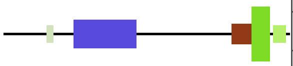		108	202	40
							67	76	21
							378	404	74
							377	390	74
							110	138	40
							409	428	21
							346	376	29
22	Not clustered	PVX_024185	variable surface protein Vir6-like	427	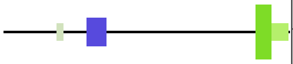		81	89	21
							377	398	74
							125	153	40
							400	423	21
23	Not clustered	PVX_039190	variable surface protein Vir6, putative	438	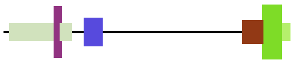		77	89	72
							79	87	72
							397	423	74
							396	410	74
							123	151	40
							77	84	21
							10	105	21
							425	438	21
							77	84	72
							365	396	29
24	G	PVX_014630	variable surface protein Vir6, putative	464	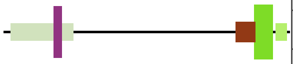		82	93	72
							408	434	74
							407	420	74
							13	113	21
							441	458	21
							376	406	29
							82	93	72
25	Not clustered	PVX_033690	variable surface protein Vir6, truncated, putative	105	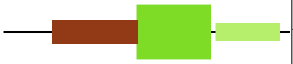		50	76	74
							49	63	74
							78	101	21
							18	49	29
26	Not clustered	PVX_030690	variable surface protein Vir6, putative	186	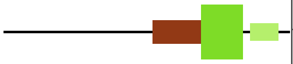		129	155	74
							129	147	74
							160	178	21
							97	128	29
27	Not clustered	PVX_031190	variable surface protein Vir6, putative	319	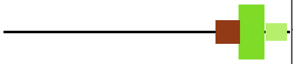		264	290	74
							263	277	74
							292	315	21
							237	263	29
28	Not clustered	PVX_040190	variable surface protein Vir6, truncated, putative	266	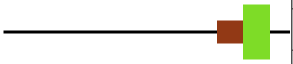		224	247	74
							223	243	74
							199	222	29

29	G	PVX_047190	variable surface protein Vir28-like	325	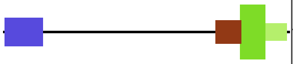		270	296	74
							2	45	40
							269	283	74
							2	19	40
							298	321	21
							241	269	29
30	Not clustered	PVX_057190	variable surface protein Vir6, truncated, putative	118	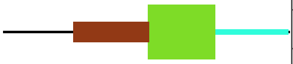		110	116	8
							86	117	8
							61	87	74
							60	74	74
							29	60	29
31	Not clustered	PVX_018655	variable surface protein Vir28-like	515	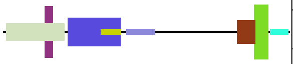		76	89	72
							184	208	6
							123	210	40
							479	510	8
							78	86	72
							224	250	6
							250	272	6
							74	83	21
							453	476	74
							118	169	40
							177	210	6
							453	476	74
							119	148	40
							7	110	21
							421	451	29
32	Not clustered	PVX_032190	variable surface protein Vir28-related	497	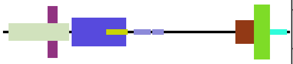		79	92	72
							188	212	6
							121	212	40
							461	492	8
							81	89	72
							227	255	6
							259	276	6
							435	460	74
							122	177	40
							181	214	6
							122	151	40
							10	113	21
							403	433	29
33	Not clustered	PVX_012115	variable surface protein Vir 28/6-related	506	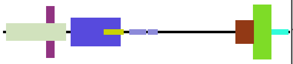		78	89	72
							185	210	6
							122	207	40
							470	501	8
							78	87	72
							224	252	6

							257	273	6
							443	469	74
							178	211	6
							442	471	74
							120	148	40
							7	110	21
							412	442	29
34		PVX_102130	hypothetical protein	456			79	91	72
							188	213	6
							126	210	40
							252	277	6
							121	178	40
							181	214	6
							122	151	40
							10	113	21
							401	431	29
35	Not clustered	PVX_048690	variable surface protein Vir7-like	408			324	345	9
							189	214	6
							324	345	9
							394	406	8
							358	379	15
							119	170	40
							189	214	6
							325	345	9
							358	370	15
							119	145	40
							9	112	21
36		PVX_102645	hypothetical protein	92			67	89	8
							39	59	74
37	Not clustered	PVX_112115	variable surface protein Vir6, putative	320			82	90	72
							84	93	72
							258	287	74
							260	271	74
							20	115	21
							82	89	72
38	Unavailable	PVX_174270	variable surface protein Vir6-like, pseudogene	475			77	89	21
							52	70	7
							52	74	7
							78	87	21
1	H	PVX_112125	variable surface protein Vir18, putative	423			120	155	10
							164	238	21
							50	71	10
							20	47	28
2		PVX_106215	hypothetical protein	328			170	230	21
							47	67	10

#	Cluster	Gene ID	Description	Length	Architecture	Color	Start	End	Domain
							16	52	28
3	Not clustered	PVX_006080	variable surface protein Vir18-related	577	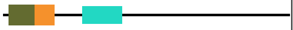		64	101	25
							161	238	21
							14	61	28
4	Unavailable	PVX_013625	variable surface protein Vir18, putative	381	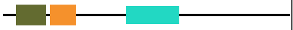		64	96	25
							164	234	21
							19	57	28
5	Not clustered	PVX_015640	variable surface protein Vir18-related	427	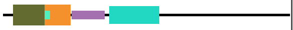		63	99	25
							103	151	10
							160	231	21
							49	70	10
							16	60	28
6		PVX_107230	hypothetical protein	416	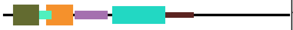		235	275	7
							63	100	25
							106	151	10
							160	234	21
							47	70	10
							16	51	28
7	Not clustered	PVX_045190	variable surface protein Vir18-related	130	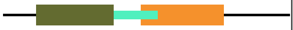		63	100	25
							46	70	10
							15	50	28
8	Not clustered	PVX_120335	variable surface protein Vir18-related	485	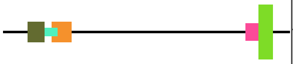		418	434	23
							412	433	23
							433	454	74
							84	115	25
							69	91	10
							43	70	28
9	Not clustered	PVX_093705	variable surface protein Vir18, putative	318	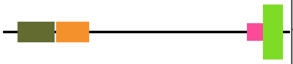		271	292	23
							289	310	74
							59	95	25
							16	56	28
10	Not clustered	PVX_121855	variable surface protein Vir18, putative	402	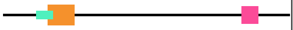		335	356	23
							63	99	25
							47	70	10
11		PVX_035690	hypothetical protein	298	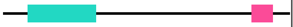		258	280	23
							268	280	23
							26	96	21
12	Not clustered	PVX_060690	variable surface protein Vir18-related	656	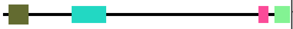		620	654	21
							584	605	23
							159	236	21
							16	57	28
13	H	PVX_068190	variable surface protein Vir18, putative	511	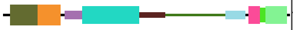		456	468	19
							437	456	23
							289	396	4
							245	286	7

							469	505	21
							240	288	7
							111	147	10
							443	455	23
							63	100	25
							289	397	4
							249	286	7
							289	397	4
							398	430	12
							168	241	21
							13	60	28
							147	236	21
							143	236	21
14	H	PVX_019165	variable surface protein Vir18, putative	503	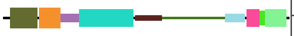		448	460	19
							429	448	23
							280	388	4
							236	277	7
							461	497	21
							232	279	7
							103	132	10
							435	447	23
							63	100	25
							280	389	4
							239	277	7
							280	389	4
							392	425	12
							159	219	21
							13	60	28
							134	227	21
15	H	PVX_104700	variable surface protein Vir18, putative	475	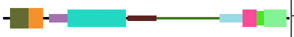		420	432	19
							397	419	23
							397	420	23
							255	357	4
							433	469	21
							207	254	7
							77	125	10
							406	419	23
							33	66	25
							255	357	4
							255	357	4
							359	395	12
							135	202	21
							13	42	28
							125	202	21
							109	202	21

16	H	PVX_021180	variable surface protein Vir18, putative	467	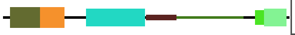		413	424	19
							283	389	4
							425	460	21
							234	282	7
							63	100	25
							283	390	4
							283	390	4
							13	60	28
							137	230	21
17	H	PVX_020675	variable surface protein Vir18, putative	495	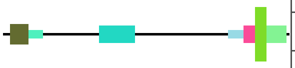		441	452	19
							417	440	23
							453	488	21
							426	439	23
							437	454	74
							389	415	12
							166	226	21
							45	69	10
							14	44	28
18	H	PVX_011110	variable surface protein Vir18-like	475	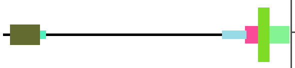		426	438	19
							403	426	23
							439	475	21
							413	425	23
							423	441	74
							365	402	12
							46	70	10
							14	60	28
19	H	PVX_083585	variale surface protein Vir18-related	548	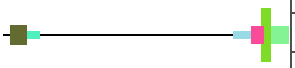		498	510	19
							475	498	23
							512	547	21
							511	547	21
							494	511	74
							485	497	23
							442	474	12
							47	71	10
							16	44	28
20	H	PVX_101625	variable surface protein Vir18-like	527	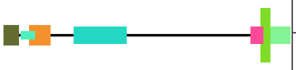		477	489	19
							454	477	23
							490	526	21
							473	489	74
							464	476	23
							49	85	25
							149	224	21
							34	56	10
							2	27	28
							130	202	21

21	H	PVX_017650	variable surface protein Vir18, putative	502	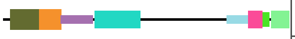		453	465	19
							430	453	23
							470	502	21
							440	452	23
							64	101	25
							102	158	10
							393	429	12
							163	239	21
							15	61	28
22	H	PVX_016640	variable surface protein Vir18-like	483	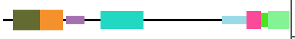		434	446	19
							411	434	23
							447	483	21
							421	433	23
							64	100	25
							107	136	10
							371	410	12
							166	235	21
							17	61	28
23	H	PVX_018150	variable surface protein Vir18, putative	510	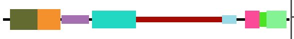		456	467	19
							432	455	23
							470	503	21
							441	454	23
							238	388	5
							63	100	25
							106	151	10
							391	415	12
							159	237	21
							13	60	28
24	H	PVX_065190	variable surface protein Vir18, truncated, putative	442	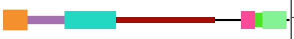		387	399	19
							368	387	23
							401	436	21
							373	386	23
							175	326	5
							1	37	25
							38	93	10
							96	174	21
25	H	PVX_010100	variable surface protein Vir18, putative	478	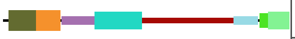		429	441	19
							443	478	21
							102	151	10
							232	383	5
							231	383	5
							57	94	25
							99	149	10
							385	424	12
							153	231	21

							11	54	28
26	Not clustered	PVX_015130	variable surface protein Vir18-like	459	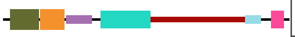		431	448	23
							237	388	5
							60	97	25
							103	141	10
							390	413	12
							158	236	21
							12	57	28
27	H	PVX_068690	variable surface protein Vir18, putative	529	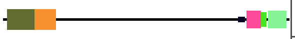		474	485	19
							451	474	23
							491	522	21
							461	473	23
							60	96	25
							435	446	5
							10	57	28
28	H	PVX_013120	variable surface protein Vir18, putative	475	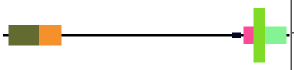		421	432	19
							400	420	23
							433	468	21
							406	419	23
							417	434	74
							60	96	25
							381	392	5
							11	57	28
29	H	PVX_017145	variable surface protein Vir18, putative	478	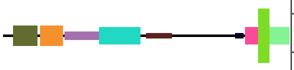		430	441	19
							406	429	23
							241	280	7
							442	478	21
							108	160	10
							415	428	23
							426	443	74
							63	100	25
							105	137	10
							239	282	7
							389	401	5
							162	228	21
							18	56	28
30	H	PVX_062690	variable surface protein Vir18, putative	462	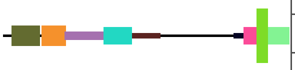		414	425	19
							390	413	23
							212	252	7
							426	462	21
							108	160	10
							399	412	23
							410	427	74
							63	100	25
							101	133	10

							210	253	7
							373	386	5
							163	207	21
							15	60	28
	31	H	PVX_241295	variable surface protein Vir18, putative	489	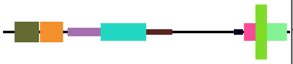	435	446	19
							411	434	23
							245	288	7
							447	482	21
							112	165	10
							420	433	23
							431	448	74
							64	101	25
							247	287	7
							394	407	5
							167	243	21
							20	61	28
	32	H	PVX_103150	variable surface protein Vir18, putative	488	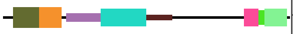	433	445	19
							410	432	23
							410	433	23
							244	287	7
							446	482	21
							111	164	10
							419	432	23
							63	100	25
							108	154	10
							246	286	7
							166	243	21
							18	60	28
	33	H	PVX_055190	variable surface protein Vir18, truncated, putative	378	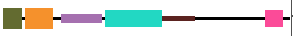	346	368	23
							210	253	7
							77	130	10
							355	368	23
							29	66	25
							212	252	7
							135	208	21
							1	24	28
	34	Not clustered	PVX_066190	variable surface protein Vir18-like	463	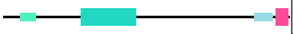	441	459	23
							451	460	23
							407	434	12
							29	53	10
							126	214	21
	1	D	PVX_074695	variable surface protein Vir10, putative	257	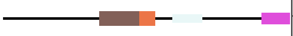	232	257	14
							122	135	20
							122	135	20
							90	121	18

							87	109	18
							152	178	11
	2	D	PVX_109785	variable surface protein Vir35, putative	261		231	258	14
							130	157	20
							130	150	20
							96	129	18
							94	116	18
							162	183	11
	3	D	PVX_103160	variable surface protein Vir10-like	254		225	252	14
							122	135	20
							122	135	20
							89	120	18
							88	109	18
							152	178	11
	4	D	PVX_182275	variable surface protein Vir11, truncated, putative	151		124	151	14
							17	44	20
							17	42	20
							1	16	18
							47	73	11
	5	D	PVX_019665	variable surface protein Vir11, putative	282		255	282	14
							130	157	20
							130	154	20
							95	129	18
							160	186	11
	6	D	PVX_178275	variable surface protein Vir11, putative	267		235	262	14
							128	155	20
							128	151	20
							93	127	18
							158	181	11
	7	D	PVX_146260	variable surface protein Vir11, putative	239		218	236	14
							129	156	20
							129	151	20
							94	128	18
							159	182	11
	8	D	PVX_041190	variable surface protein Vir35, truncated, putative	144		114	141	14
							13	40	20
							13	33	20
							45	69	11
	9	D	PVX_086850	variable surface protein Vir35, putative	245		215	242	14
							113	139	20
							113	136	20
							78	111	18
	10	D	PVX_109780	variable surface protein Vir35, putative	266		239	266	14
							114	140	20
							114	136	20

							82	110	18
							82	99	18
							227	243	7
							144	168	11
							85	96	4
11	D	PVX_108760	variable surface protein Vir35, putative	261	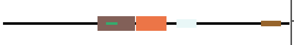		122	148	20
							125	145	20
							87	120	18
							236	252	7
							158	176	11
							94	104	4
12	D	PVX_067190	variable surface protein Vir35, putative	235	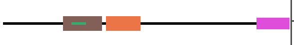		208	234	14
							85	112	20
							85	107	20
							51	80	18
							50	70	18
							57	68	4
13	Unavailable	PVX_109778	variable surface protein Vir, putative	240	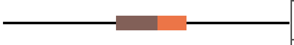		130	153	20
							130	153	20
							95	129	18
14	D	PVX_040690	variable surface protein Vir35, putative	166	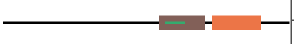		122	149	20
							122	146	20
							91	117	18
							94	105	4
15	D	PVX_143260	variable surface protein Vir35, putative	248	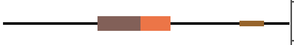		119	145	20
							119	141	20
							86	118	18
							83	106	18
							205	225	7
16	D	PVX_017645	variable surface protein Vir35, putative	230	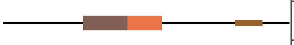		101	127	20
							101	122	20
							68	100	18
							65	88	18
							187	207	7
17	D	PVX_103145	variable surface protein Vir10, putative	168	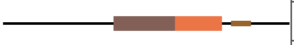		101	128	20
							101	126	20
							68	100	18
							65	88	18
							134	145	7
18	D	PVX_047690	variable surface protein Vir10, truncated, putative	267	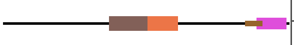		237	264	14
							135	162	20
							135	155	20
							101	134	18
							99	122	18
							226	241	7

19	D	PVX_038690	variable surface protein Vir35-like	199	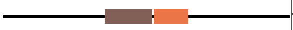		105	128	20
							105	122	20
							73	103	18
							71	90	18
20	D	PVX_102635	variable surface protein Vir10-related	198	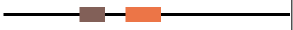		85	108	20
							85	102	20
							53	70	18
21	Not clustered	PVX_058190	variable surface protein Vir10/35, truncated, putative	123			94	121	14
							1	15	11
1	Not clustered	PVX_096005	variable surface protein Vir15-related	367	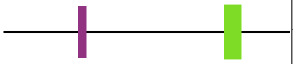		97	104	72
							96	105	72
							98	106	72
							284	303	74
2	Not clustered	PVX_121355	variable surface protein Vir15-like	222	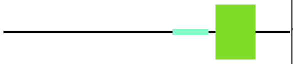		132	158	5
							165	195	74
3	J	PVX_057690	variable surface protein Vir33, putative	301	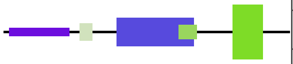		80	92	21
							120	199	40
							80	92	21
							80	92	21
							7	69	11
							122	179	40
							241	271	74
							80	92	21
							184	202	17
4	J	PVX_109795	variable surface protein Vir33, putative	301	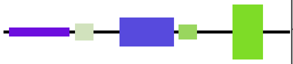		77	93	21
							76	94	21
							77	94	21
							7	69	11
							122	179	40
							241	271	74
							77	94	21
							184	202	17
5	J	PVX_070690	variable surface protein Vir33, truncated, putative	260	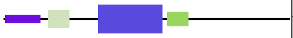		42	58	21
							42	54	21
							41	59	21
							42	59	21
							2	33	11
							87	144	40
							42	59	21
							149	167	17
6	J	PVX_067690	variable surface protein Vir1-like	266	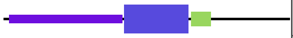		6	110	11
							112	171	40
							175	192	17
7	J	PVX_105710	variable surface	245	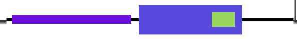		6	106	11

						120	200	40
						113	171	40
						121	143	40
						176	194	17
8	J	PVX_000020	variable surface protein Vir9-like	234	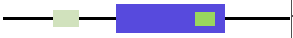	99	181	40
						41	61	21
						93	150	40
						157	173	17
9	J	PVX_096930	variable surface protein Vir1-related, truncated	248	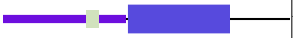	1	106	11
						112	195	40
						73	82	21
						108	167	40
10	Not clustered	PVX_106720	variable surface protein Vir33 (truncated), putative	220	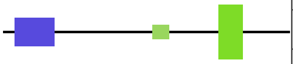	166	182	74
						166	182	74
						168	183	74
						9	39	40
						168	181	74
						171	183	74
						115	127	17
11	J	PVX_096935	variable surface protein Vir9-related	304	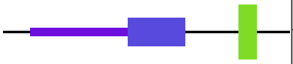	250	266	74
						250	266	74
						29	131	11
						133	192	40
						254	269	74
						256	266	74
12	Unavailable	PVX_096925	variable surface protein Vir15-like	289	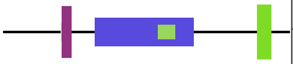	257	269	74
						257	269	74
						93	191	40
						98	153	40
						60	68	72
						257	269	74
						258	269	74
						59	68	21
						60	68	72
						157	172	17
13	J	PVX_167265	variable surface protein Vir15, truncated, putative	278	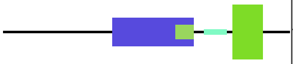	195	216	5
						108	184	40
						223	248	74
						107	162	40
						223	251	74
						168	184	17
14	J	PVX_010605	variable surface protein Vir15, putative	286	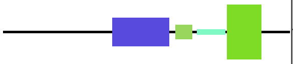	194	221	5
						228	253	74
						110	165	40
						227	257	74

							224	244	74
							172	188	17
15	Not clustered	PVX_186290	variable surface protein Vir15-related, truncated	133	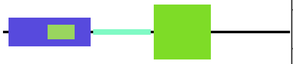		42	68	5
							3	40	40
							75	96	74
							70	88	74
							21	33	17
16	Unavailable	PVX_168270	variable surface protein Vir15, pseudogene, putative	343	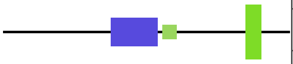		290	308	74
							129	184	40
							191	207	17
17	J	PVX_107750	variable surface protein Vir2/15-like	301	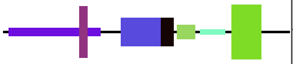		207	232	5
							8	102	11
							7	65	11
							124	178	40
							240	270	74
							167	178	40
							183	201	17
							80	88	72
18		PVX_105705	hypothetical protein	191	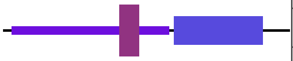		6	110	11
							78	87	21
							114	172	40
							78	90	72
							78	86	21
							78	86	72
							78	84	72
							78	88	72
							78	87	72
19	Unavailable	PVX_185285	variable surface protein Vir2, truncated, putative	345	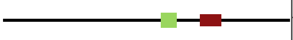		238	262	13
							191	208	17
1	Not clustered	PVX_032690	variable surface protein Vir7, putative	156	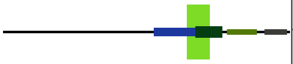		100	112	74
							82	103	9
							100	112	74
							82	104	9
							142	154	8
							83	104	9
							105	119	15
							122	138	6
2	Not clustered	PVX_054690	variable surface protein Vir29, truncated, putative	51			37	49	8
							2	26	15
							1	15	15
							16	35	6
3	I	PVX_150260	variable surface protein Vir27, truncated,	361	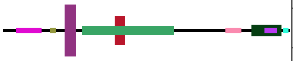		60	66	8
							327	350	15
							314	350	15

			putative				135	152	12
							166	184	6
							18	41	8
							330	343	6
							314	326	15
							78	91	72
							280	299	6
							142	152	39
							352	359	8
							28	48	8
							100	214	12
							100	214	12
4	I	PVX_053190	variable surface protein Vir27, putative	344	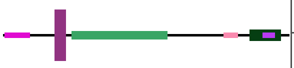		310	333	15
							297	333	15
							118	135	12
							149	167	6
							2	25	8
							313	326	6
							297	309	15
							62	74	72
							265	282	6
							12	32	8
							83	197	12
							83	197	12
5	I	PVX_064690	variable surface protein Vir27, putative	363	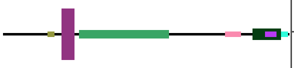		57	65	8
							316	351	15
							131	147	12
							333	345	6
							316	328	15
							76	89	72
							282	301	6
							353	361	8
							98	210	12
							98	210	12
							78	90	72
							78	89	72
6	I	PVX_102630	variable surface protein Vir27, putative	345	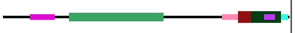		34	61	8
							298	334	15
							113	131	12
							37	57	8
							314	327	6
							264	282	6
							264	283	6
							335	343	8
							44	62	8

							80	192	12
							283	297	13
							80	192	12
7	Not clustered	PVX_055690	variable surface protein Vir27, truncated, putative	192	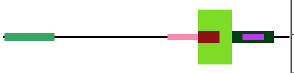		131	153	74
							131	153	74
							154	181	15
							145	181	15
							161	174	6
							111	130	6
							141	153	74
							1	34	12
							131	144	13
							1	34	12
8	I	PVX_002495	variable surface protein Vir7-like	324	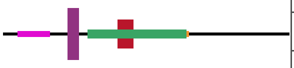		130	146	39
							17	53	8
							76	85	72
							184	210	6
							136	147	39
							184	210	6
							97	206	12
							97	206	12
							74	85	72
9	Not clustered	PVX_048190	variable surface protein Vir27, truncated, putative	117	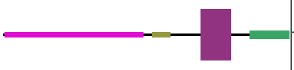		61	68	8
							1	57	8
							19	42	8
							81	92	72
							29	50	8
							101	115	12
							101	117	12
							81	93	72
10	I	PVX_177275	variable surface protein Vir7, putative	328	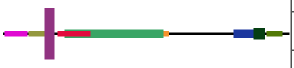		30	46	8
							3	27	8
							265	285	9
							265	286	9
							102	131	12
							265	286	9
							287	300	15
							49	59	72
							132	152	6
							156	190	6
							156	190	6
							71	183	12
							63	100	5
							71	183	12
							63	100	5

								303	319	6
	11	I	PVX_064190	variable surface protein Vir7, putative	364	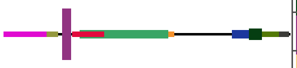		56	68	8
								1	54	8
								290	311	9
								290	312	9
								350	362	8
								129	159	12
								291	312	9
								313	327	15
								76	85	72
								160	180	6
								181	215	6
								181	215	6
								97	208	12
								89	127	5
								97	208	12
								89	127	5
								329	348	6
	12	I	PVX_072190	variable surface protein Vir7, putative	362	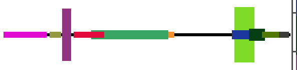		58	72	8
								1	54	8
								288	309	9
								292	316	74
								288	310	9
								348	360	8
								312	329	15
								128	158	12
								289	310	9
								311	325	15
								75	85	72
								159	179	6
								180	214	6
								180	214	6
								111	207	12
								89	126	5
								115	207	12
								89	126	5
								327	346	6
	13	I	PVX_014625	variable surface protein Vir7, putative	375	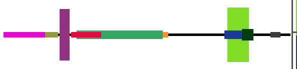		56	71	8
								1	54	8
								289	310	9
								293	319	74
								289	311	9
								349	360	8
								128	156	12
								290	311	9

							312	325	15
							76	85	72
							159	179	6
							180	214	6
							180	214	6
							97	207	12
							89	126	5
							97	207	12
							74	83	72
							89	126	5
14	Unavailable	PVX_248300	variable surface protein Vir29, pseudogene, putative	400	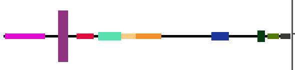		3	57	8
							290	311	9
							290	312	9
							386	398	8
							133	163	12
							164	182	6
							292	312	9
							355	363	15
							77	88	72
							184	218	6
							184	218	6
							103	125	5
							103	125	5
							369	382	6
15	I	PVX_023685	variable surface protein Vir7, putative	292	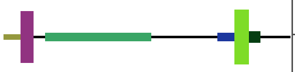		236	248	74
							1	17	8
							218	239	9
							218	239	9
							242	260	15
							75	104	12
							105	123	6
							219	239	9
							241	254	15
							20	30	72
							236	249	74
							43	149	12
							43	149	12
							18	29	72
16	I	PVX_069190	variable surface protein Vir27-like, truncated	343	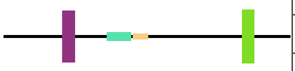		286	298	74
							124	151	12
							155	173	6
							71	82	72
							286	298	74
							287	299	74
							71	85	72

17	Not clustered	PVX_039690	variable surface protein Vir27, truncated, putative	66			19	55	15
							35	48	6
							56	64	8
1	B	PVX_086350	variable surface protein Vir21, putative	397	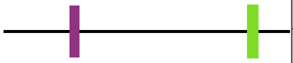		341	351	74
							339	352	74
							342	350	74
							93	105	72
2	Not clustered	PVX_108775	variable surface protein Vir26, truncated, putative	62	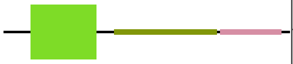		24	46	8
							6	18	74
							7	20	74
							47	60	7
3	B	PVX_088790	variable surface protein Vir21-like	467	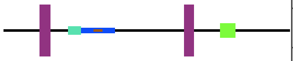		127	181	7
							353	376	17
							296	309	72
							149	161	4
							107	126	12
							298	305	72
							60	75	72
							296	310	72
							64	74	72
4	B	PVX_096915	variable usrface prootein Vir21-related	295	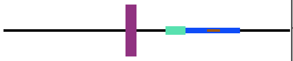		188	242	7
							210	222	4
							168	187	12
							126	137	72
							126	136	72
							126	135	72
5	B	PVX_110305	variable surface protein Vir21, putative	267	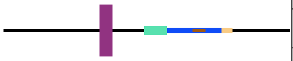		152	203	7
							152	207	7
							204	213	6
							177	187	4
							132	151	12
							90	100	72
							90	101	72
							90	101	72
							90	101	72
6	B	PVX_115480	variable surface protein Vir21, putative	276	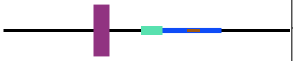		153	209	7
							178	188	4
							133	152	12
							90	102	72
							88	100	72
							91	102	72
7	B	PVX_180275	variable surface protein Vir26-like, truncated	236	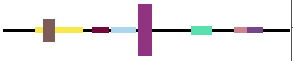		201	213	6
							27	66	7
							155	172	12
							191	200	6

								201	213	6
								34	42	30
								89	109	5
								111	122	72
								111	122	72
								111	122	72
								74	87	8
	8	B	PVX_075195	variable surface protein Vir26, putative	302	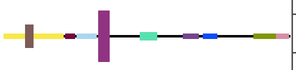		264	286	8
								211	224	7
								189	204	6
								1	62	7
								144	161	12
								288	300	7
								189	204	6
								23	31	30
								78	98	5
								100	111	72
								100	111	72
								100	111	72
								65	75	8
	9	B	PVX_103680	variable surface protein Vir21, putative	361	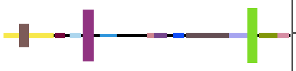		121	141	4
								230	283	6
								323	345	8
								213	226	7
								191	205	6
								1	62	7
								308	318	74
								346	359	7
								285	306	6
								181	190	6
								191	205	6
								21	31	30
								84	97	5
								101	113	72
								102	111	72
								65	76	8
	10	B	PVX_025690	variable surface protein Vir21, putative	351	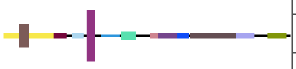		121	141	4
								229	283	6
								323	345	8
								212	225	7
								189	211	6
								1	62	7
								144	161	12
								286	306	6
								180	188	6

							190	211	6
							21	31	30
							85	98	5
							102	111	72
							63	76	8
11	B	PVX_033190	variable surface protein Vir21, putative	357	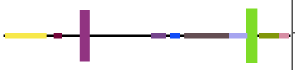		225	279	6
							319	341	8
							208	219	7
							185	201	6
							3	53	7
							303	315	74
							344	354	7
							281	302	6
							187	201	6
							96	107	72
							96	107	72
							64	72	8
12	B	PVX_058690	variable surface protein Vir21, truncated, putative	299	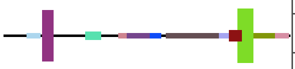		169	223	6
							245	258	74
							261	283	8
							245	257	74
							152	164	7
							129	151	6
							246	259	74
							86	101	12
							284	297	7
							225	234	6
							225	245	6
							121	128	6
							130	151	6
							24	38	5
							235	248	13
							41	51	72
							42	50	72
13	B	PVX_062190	variable surface protein Vir21, truncated, putative	326	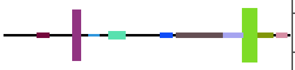		97	109	4
							196	248	6
							288	305	8
							271	288	74
							178	191	7
							272	284	74
							120	137	12
							311	322	7
							250	271	6
							79	87	72
							39	52	8

14	B	PVX_164265	variable surface protein Vir21, putative	362	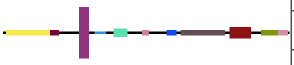		117	129	4
							225	279	6
							326	346	8
							208	219	7
							4	58	7
							140	157	12
							348	359	7
							176	184	6
							96	107	72
							287	313	13
							96	107	72
							96	108	72
							61	69	8
15	Not clustered	PVX_059690	variable surface protein Vir26, putative	88	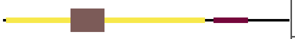		1	62	7
							21	31	30
							65	75	8
1	K	PVX_053690	variable surface protein Vir30, putative	309	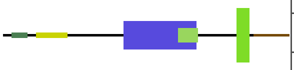		10	26	5
							271	309	4
							278	306	4
							130	208	40
							37	68	6
							189	210	17
							253	265	74
							252	264	74
							195	207	17
2	K	PVX_042690	variable surface protein Vir30, putative	337	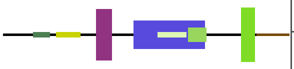		36	54	5
							299	337	4
							305	334	4
							155	237	40
							63	90	6
							218	238	17
							111	127	72
							281	295	74
							183	214	5
							280	292	74
3	Unavailable	PVX_163265	variable surface protein Vir30, pseudogene, putative	345	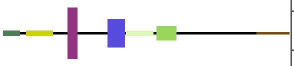		1	19	5
							307	345	4
							314	342	4
							78	88	72
							28	59	6
							185	207	17
							78	89	72
							126	146	40
							150	181	5
4	K	PVX_145260	variable surface	305	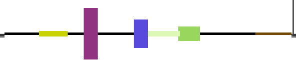		267	305	4

						273	302	4
						38	67	6
						186	207	17
						84	99	72
						138	152	40
						153	185	5
5	K	PVX_031690	variable surface protein Vir30, putative	268	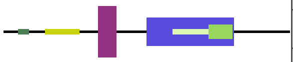	14	24	5
						134	215	40
						40	71	6
						192	214	17
						89	105	72
						137	158	40
						159	191	5
6	K	PVX_056690	variable surface protein Vir30, truncated, putative	190	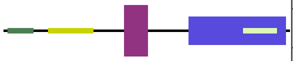	3	19	5
						123	187	40
						30	59	6
						80	95	72
						159	181	5
7	Not clustered	PVX_021685	variable surface protein Vir5, putative	426	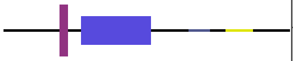	117	218	40
						85	95	72
						275	305	4
						332	369	4
8	Not clustered	PVX_093715	variable surface protein Vir5-like	499	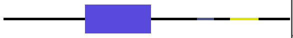	144	256	40
						338	366	4
						395	443	4
9	Unavailable	PVX_156260	variable surface proteinVir5, pseudogene, putative; truncated due to end of contig at 3'. 5' interrupted.	213		14	44	4
						71	108	4
10	Not clustered	PVX_101570	variable surface protein Vir5-related	386	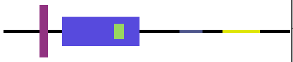	51	59	72
						81	182	40
						239	267	4
						50	59	72
						296	344	4
						150	162	17
11	Not clustered	PVX_009600	variable surface protein Vir5-related	259	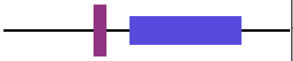	115	214	40
						82	92	72
12		PVX_005565	hypothetical protein	209	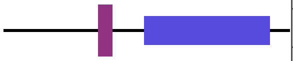	103	194	40
						70	79	72
						70	79	72
13		PVX_086885	hypothetical protein	266	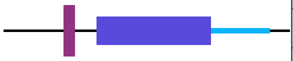	159	247	7
						87	192	40
						56	66	72

14		PVX_115990	hypothetical protein	271	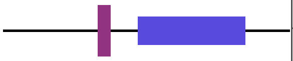		128	228	40
							90	101	72
							135	190	40
							131	159	40
15	Not clustered	PVX_001610	variable surface protein Vir25-related	338	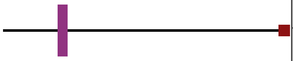		65	75	72
							325	337	13
1	A	PVX_161260	variable surface protein Vir3, truncated, putative	209	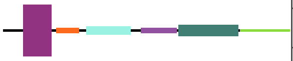		128	171	13
							21	30	72
							23	30	72
							101	126	8
							61	93	11
							173	209	4
							39	55	7
							15	35	72
							21	28	72
2	A	PVX_160260	variable surface protein Vir3, truncated, putative	166	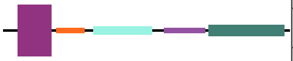		119	162	13
							13	22	72
							15	23	72
							93	117	8
							53	86	11
							31	47	7
							9	28	72
							13	21	72
							13	20	72
3	Unavailable	PVX_139260	variable surface protein Vir3, truncated,putative	362	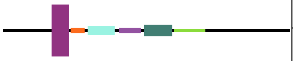		179	213	13
							153	173	8
							148	173	8
							108	140	11
							216	254	4
							86	102	7
							62	82	72
							68	77	72
4	Unavailable	PVX_155260	variable surface rptoein Vir3, pseudogene, putative; No 5' found.	369	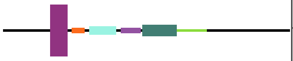		179	222	13
							157	177	8
							153	177	8
							112	144	11
							224	261	4
							89	105	7
							61	83	72
							68	76	72
5	Unavailable	PVX_186285	variable surface protein Vir3, truncated, pseudogene	306	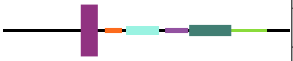		199	242	13
							177	188	8
							173	197	8
							133	166	11
							244	281	4

						110	126	7
						83	100	72
						89	98	72
						89	96	72
						89	98	72
6	A	PVX_015135	variable surface protein Vir17, truncated, putative	383	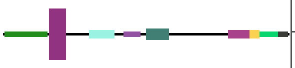	192	221	13
						343	366	6
						368	381	8
						343	367	6
						343	366	6
						330	342	9
						328	342	9
						3	57	6
						162	182	8
						302	329	9
						116	148	11
						3	58	6
						62	83	72
						343	367	6
						3	58	6
						69	78	72
7	A	PVX_011610	variable surface protein Vir17, putative	362	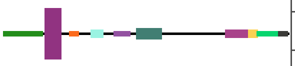	170	200	13
						322	345	6
						347	360	8
						322	346	6
						322	345	6
						309	321	9
						307	321	9
						1	45	6
						140	160	8
						281	308	9
						112	126	11
						1	49	6
						85	95	7
						54	73	72
						322	346	6
						1	48	6
						59	68	72
8	A	PVX_141260	variable surface protein Vir17, putative	385	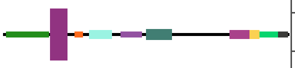	193	227	13
						345	368	6
						370	383	8
						345	369	6
						345	368	6
						333	344	9
						330	344	9

							5	59	6
							158	185	8
							305	331	9
							117	146	11
							5	61	6
							97	107	7
							64	86	72
							345	369	6
							5	60	6
							71	80	72
9	A	PVX_018155	variable surface protein Vir17, putative	393	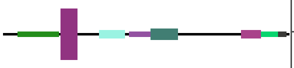		203	238	13
							354	376	6
							378	389	8
							354	376	6
							354	376	6
							21	75	6
							174	201	8
							327	352	9
							133	166	11
							21	75	6
							80	102	72
							354	376	6
							21	75	6
							87	96	72
10	A	PVX_019160	variable surface protein Vir17, putative	394	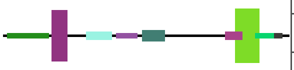		321	341	74
							192	221	13
							348	371	6
							373	384	8
							321	341	74
							348	371	6
							348	371	6
							333	347	9
							329	341	74
							6	60	6
							157	184	8
							307	334	9
							116	149	11
							330	352	74
							6	62	6
							329	341	74
							67	87	72
							348	371	6
							6	61	6
11	Unavailable	PVX_181275	variable surface protein Vir17,	485	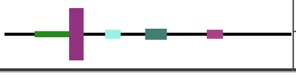		238	273	13
							51	105	6

			pseudogene, putative				342	367	9
							171	194	11
							51	107	6
							110	132	72
							51	105	6
							117	126	72
12	Not clustered	PVX_153260	variable surface protein Vir17, truncated, putative	67			27	50	6
							52	65	8
							27	51	6
							27	50	6
							12	26	9
							27	51	6
13	Unavailable	PVX_171270	variable surface protein Vir17, pseudogene, putative	253			19	56	13
							199	213	9
							136	150	9
							152	161	9
14	Not clustered	PVX_144260	variable surface protein Vir17, putative, pseudogene	430			314	339	9
15	A	PVX_112645	variable surface protein Vir17-like	321			208	227	13
1	Not clustered	PVX_096980	variable surface protein Vir, putative	396			163	179	4
							281	295	74
							284	291	74
2	Not clustered	PVX_096970	variable surface protein Vir8-related	491			166	184	4
							285	304	74
							287	296	74
3	Not clustered	PVX_176270	variable surface protein Vir8	339			60	70	28
							281	300	74
							279	287	74
4		PVX_096975	hypothetical protein	556			172	183	4
							281	302	74
5		PVX_096965	hypothetical protein	285			119	136	4
6	Not clustered	PVX_096985	variable surface protein Vir, putative	387			260	276	74
							146	165	4
							268	292	74
							268	284	74
7	Not clustered	PVX_120840	variable surface protein Vir8-related	324			163	174	4
							145	208	40
							50	59	28
8		PVX_123205	CAF1 ribonuclease domain containing protein	2024					

9		PVX_092630	hypothetical protein, conserved	2712					
10		PVX_119620	hypothetical protein, conserved	1384					
11		PVX_183275	hypothetical protein	391					
12	Not clustered	PVX_086890	variable surface protein Vir8-like	271					
1	Not clustered	PVX_173270	variable surface protein Vir23, putative	346	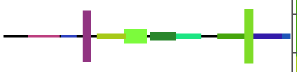		336	345	5
							70	87	4
							146	172	17
							31	67	4
							207	238	5
							258	290	6
							275	290	6
							275	293	6
							113	145	7
							113	147	7
							178	206	10
							291	301	74
							302	334	5
							181	199	10
							96	104	72
2	Not clustered	PVX_011615	variable surface protein vir23, putative	351	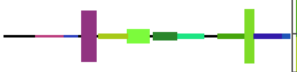		341	351	5
							75	91	4
							152	178	17
							40	72	4
							212	244	5
							263	295	6
							280	295	6
							280	298	6
							117	151	7
							117	152	7
							183	211	10
							296	306	74
							307	339	5
							186	204	10
							96	112	72
							101	113	72
3	Not clustered	PVX_051190	variable surface protein Vir23, truncated, putative	286	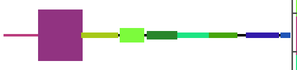		276	286	5
							36	53	4
							116	139	17
							1	33	4
							173	205	5
							206	230	6
							205	230	6

						215	233	6
						35	78	72
						78	113	7
						144	172	10
						242	274	5
						147	165	10
						57	73	72
						62	74	72
4	Unavailable	PVX_103675	variable surface protein Vir23, pseudogene, putative	462	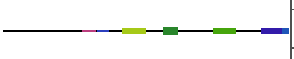	452	461	5
						154	169	4
						129	149	4
						340	372	6
						357	375	6
						194	229	7
						260	281	10
						418	450	5
						263	274	10
5	Not clustered	PVX_069690	variable surface protein Vir23, putative	327	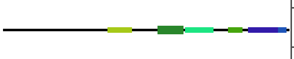	315	323	5
						208	239	5
						258	272	6
						120	147	7
						178	206	10
						281	313	5
						181	199	10
6	Unavailable	PVX_005047	variable surface protein Vir22/23-related	527	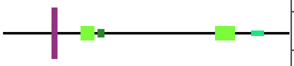	458	478	5
						144	167	17
						391	425	17
						174	186	10
						90	100	72
7	Not clustered	PVX_086880	variable surface protein Vir23-like	268	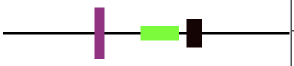	129	164	17
						173	186	10
						172	186	40
						87	94	72
8	Not clustered	PVX_096940	variable surface protein Vir24-related	292	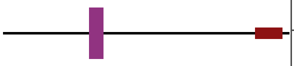	89	99	72
						257	284	13
						89	101	72
						90	99	72
9	Not clustered	PVX_093725	variable surface protein Vir24-related	392	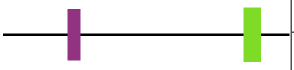	332	344	74
						90	105	72
						330	344	74
						334	343	74
						92	101	72
						333	351	74
						90	98	72
						90	102	72

						90	104	72
1	Not clustered	PVX_131260	variable surface protein Vir12, truncated, putative	278	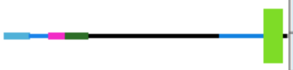	255	272	74
						211	253	4
						49	58	7
						45	58	7
						254	272	74
						1	24	5
						1	24	5
						25	48	4
						59	82	8
						60	82	8
						44	58	7
						44	58	7
2	Not clustered	PVX_043190	variable surface protein Vir12, truncated, putative	319	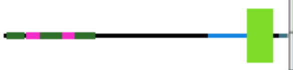	273	300	74
						229	271	4
						65	77	7
						26	39	7
						272	300	74
						40	63	8
						79	101	8
						2	22	8
						308	317	4
						41	63	8
						80	101	8
						2	22	8
						25	39	7
						65	77	7
						25	39	7
						65	77	7
3	Not clustered	PVX_130260	variable surface protein Vir12, truncated, putative	108	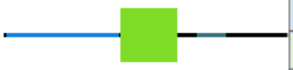	45	65	74
						1	43	4
						44	65	74
						73	84	4
4	Not clustered	PVX_129260	variable surface protein Vir12, putative	59	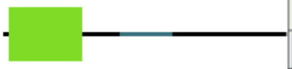	1	16	74
						1	16	74
						24	35	4
						8	16	74
