# Supplementary material for: A new computational approach redefines the subtelomeric vir superfamily of Plasmodium vivax
Source: BMC Genomics. 2013 Jan 16;14:8. doi: 10.1186/1471-2164-14-8 (PMC3566924; doi:10.1186/1471-2164-14-8)
Supplement: Additional file 7 — Comparison with OrthoMCL5. Results of the comparison between vir , Pvpir (sub)families and OrthoMCL5 groups. [file 1471-2164-14-8-S7.pdf]

Comparison with OrthoMCL5

Description: newly defined *vir* subfamilies, as well as the new *Pvpir* families were compared with OrthoMCL5 groups. Venn diagrams show the number of genes in common, and those specific for each (sub)family and OrthoMCL5 groups. OrthoMCL5 groups are indicated with their OrthomCL5 id.

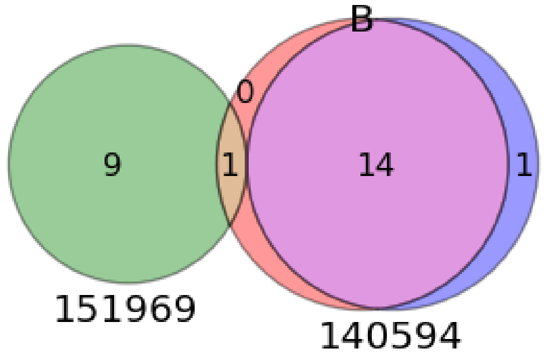

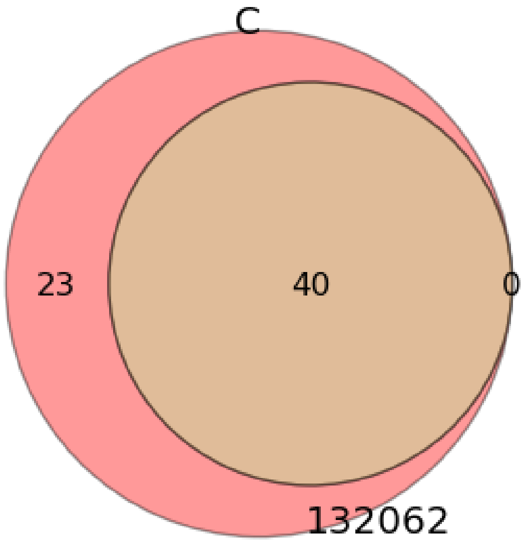
C
23
40
0
132062

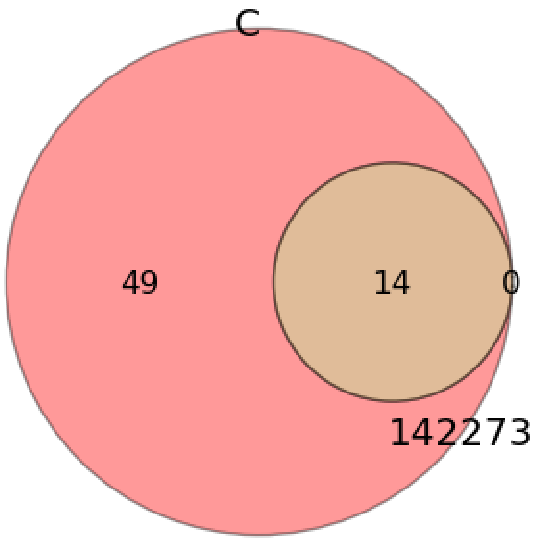
C
49
14
0
142273

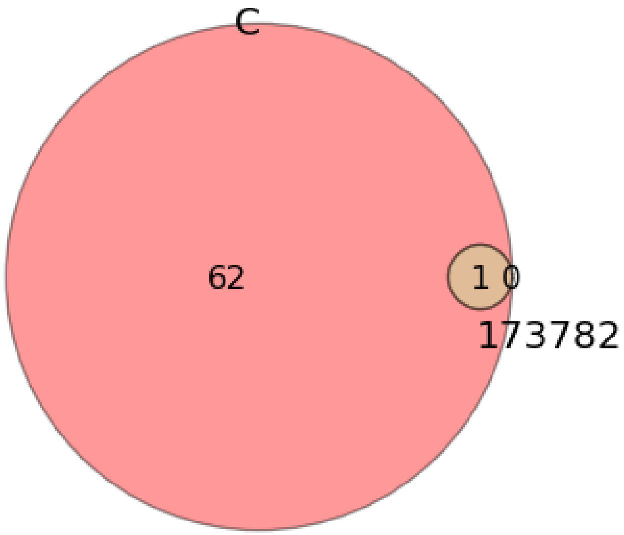
C
62
1 0
173782

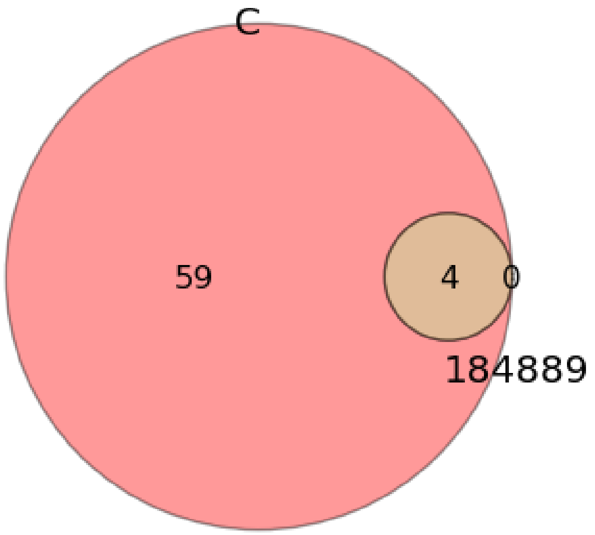
C
59
4
0
184889

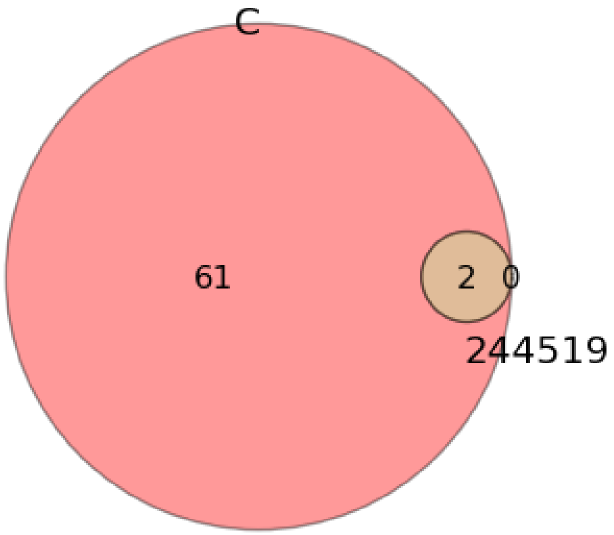
C
61
2
0
244519

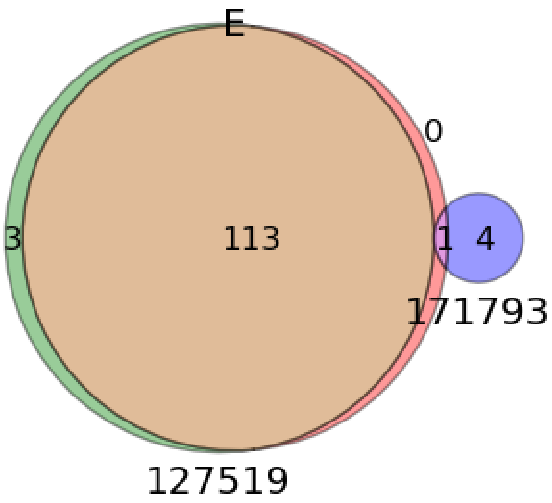
E
0
3
113
1
4
171793
127519

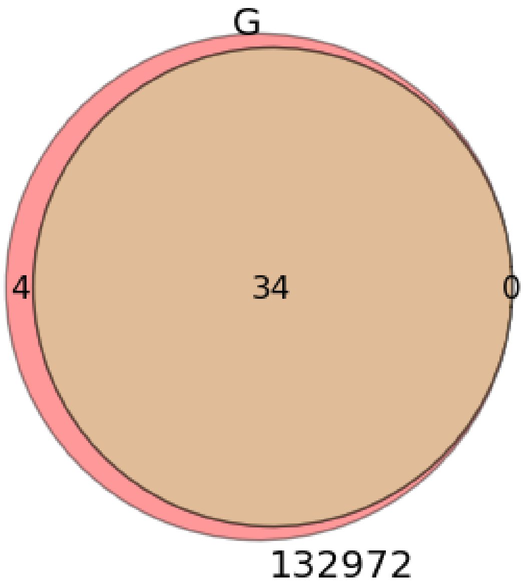
G
4
34
0
132972

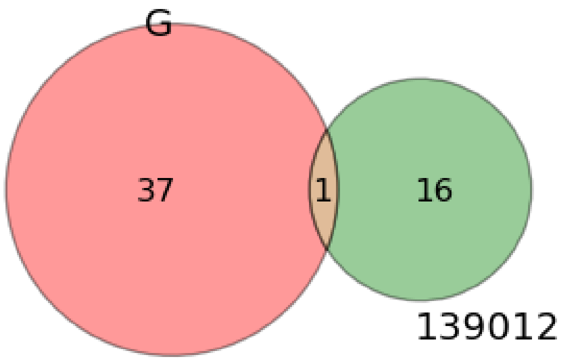
G
37
1
16
139012

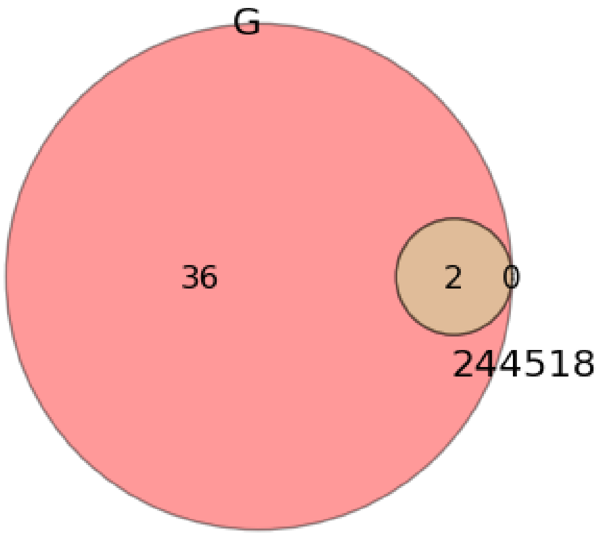
G
36
2
0
244518

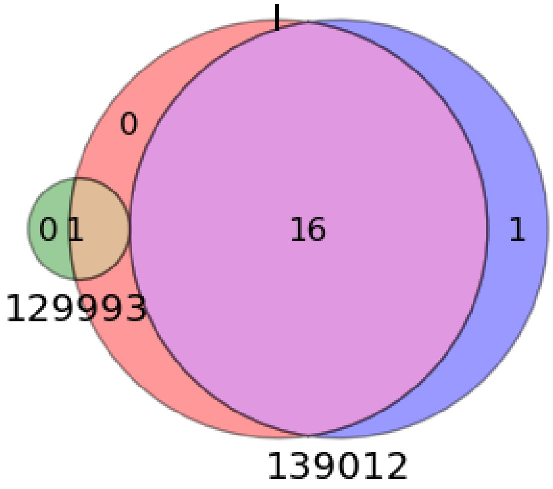
I
0
0
1
16
1
129993
139012

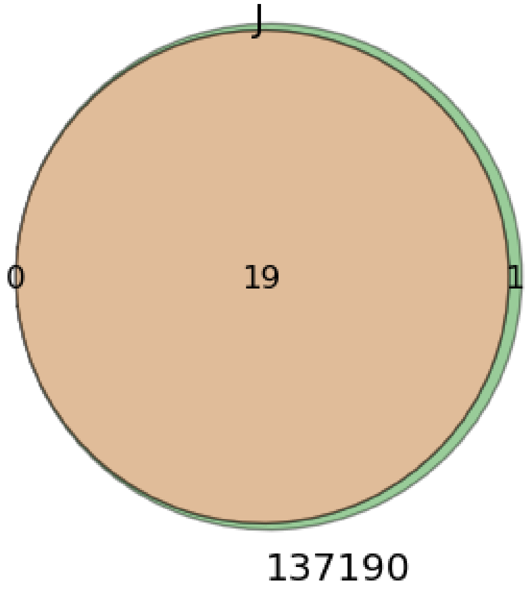
J
0
19
1
137190

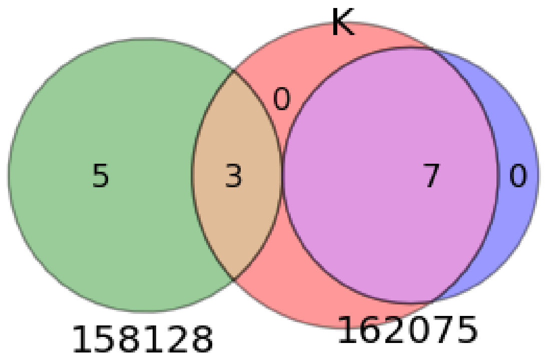
K
0
5
3
7
0
158128
162075

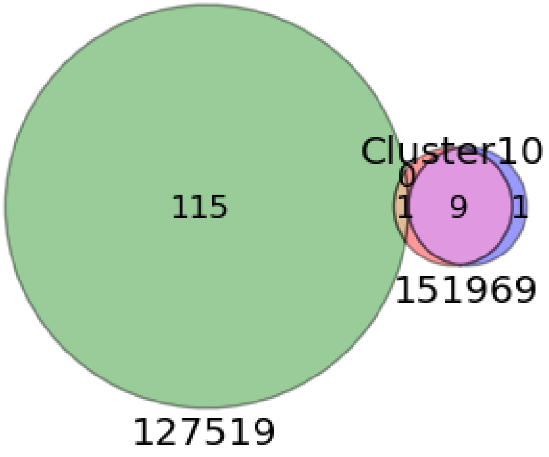
Cluster10
0
115
1
9
1
151969
127519

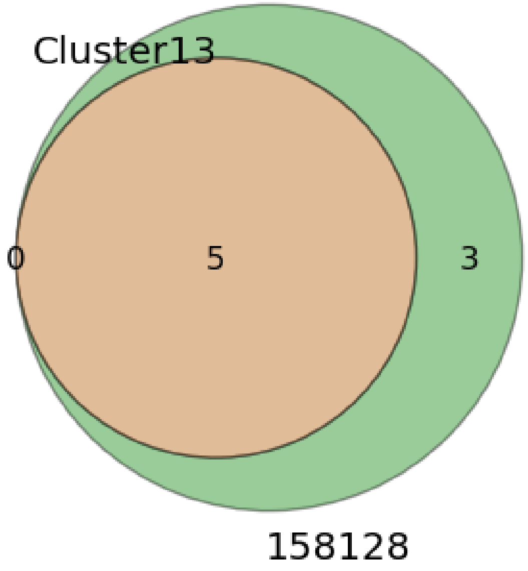
Cluster13
0
5
3
158128

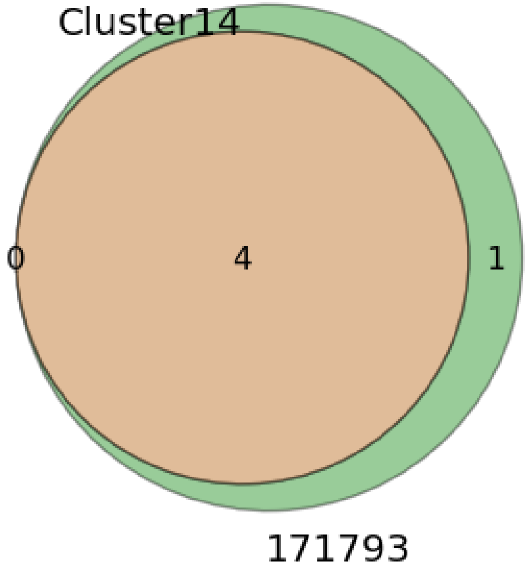
Cluster14
0
4
1
171793

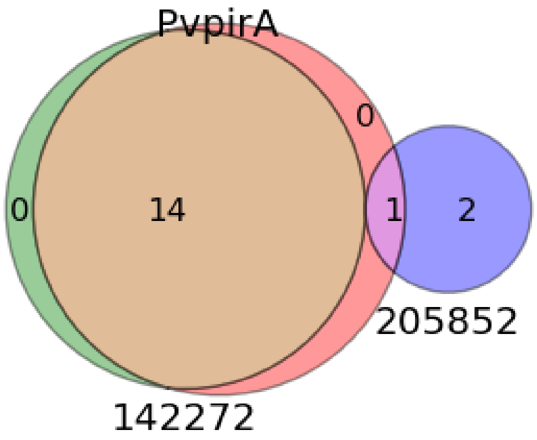
PvpirA
0
0
14
1
2
205852
142272

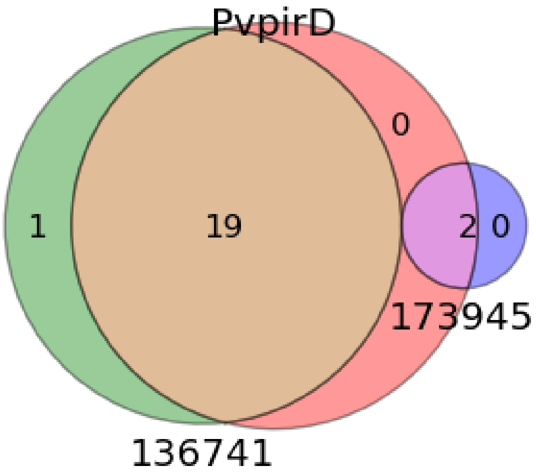
PvpirD
0
1
19
2
0
173945
136741

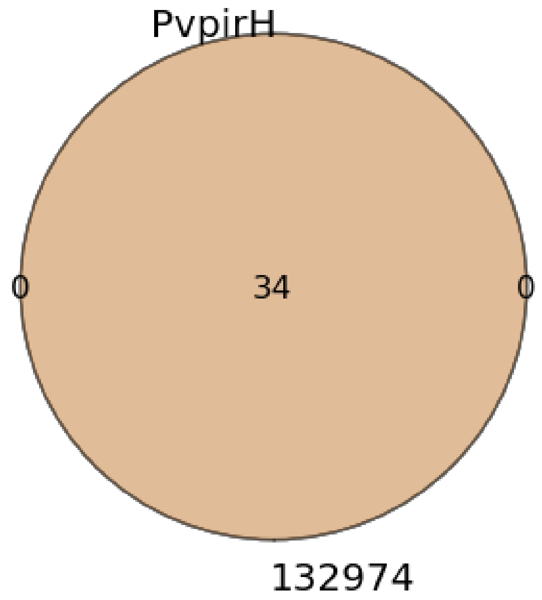
PvpirH
0
34
0
132974

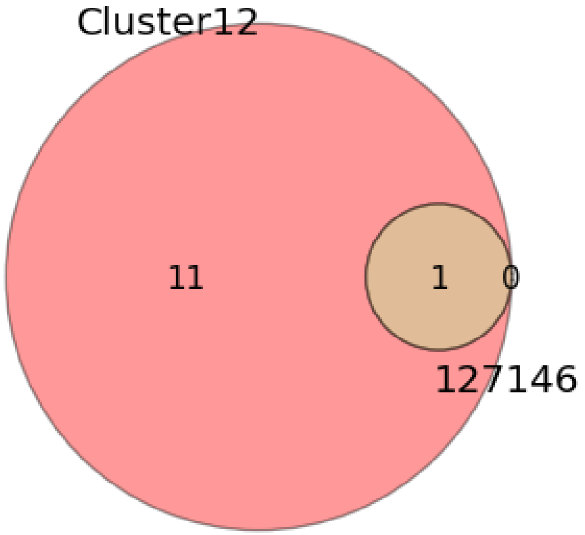
Cluster12
11
1
0
127146

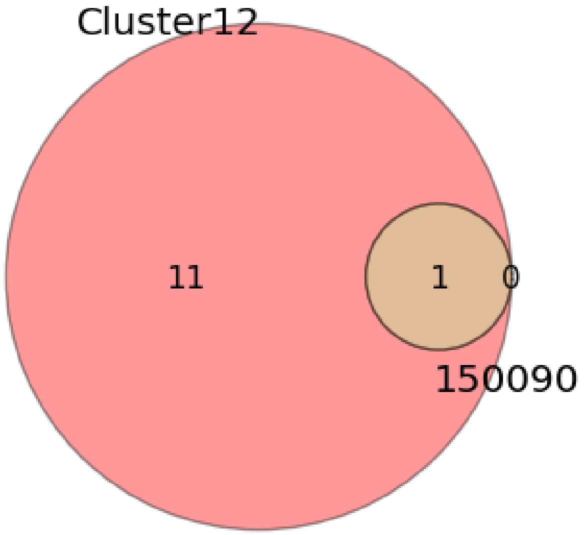
Cluster12
11
1
0
150090

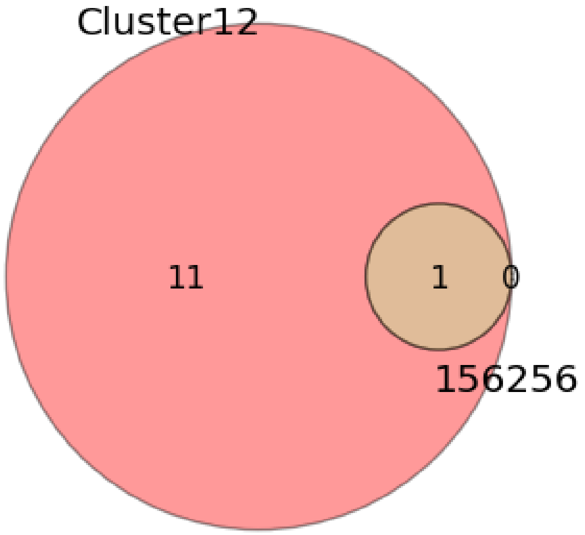
Cluster12
11
1
0
156256

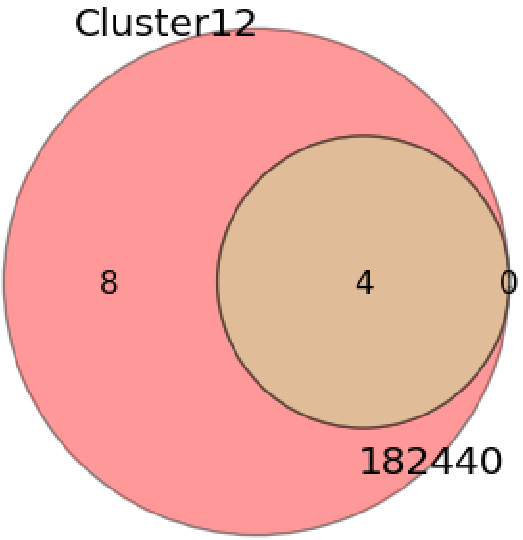
Cluster12
8
4
0
182440

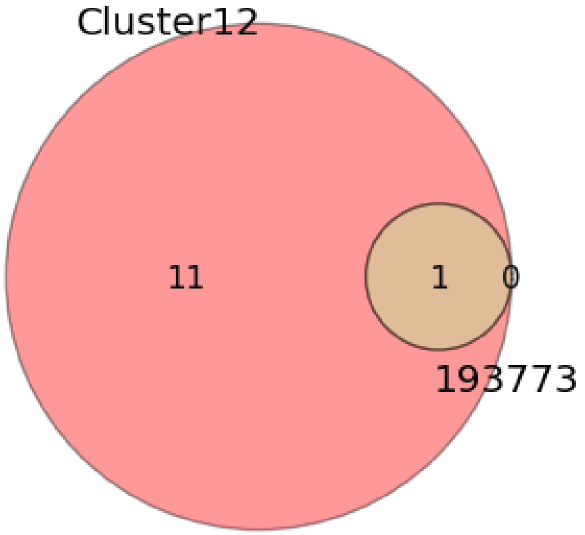
Cluster12
11
1
0
193773

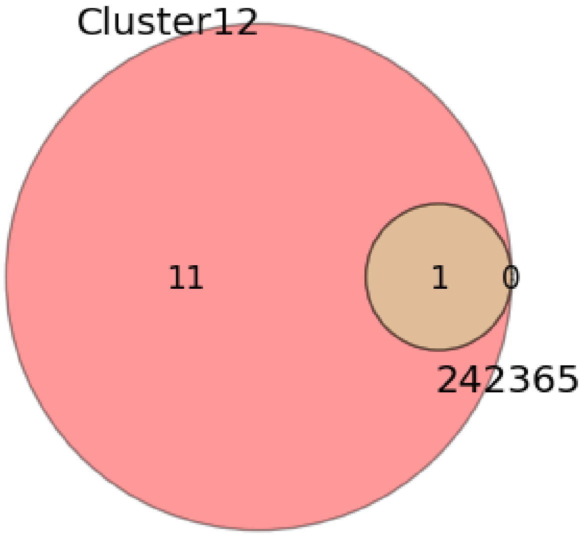
Cluster12
11
1
0
242365
